# The regulation of protein phosphatase 4 by FBXO42 is required for cancer cell survival

**DOI:** 10.1038/s44319-026-00828-y

**Published:** 2026-07-14

**Authors:** Stephan H Spangenberg, Nicholas Garaffo, Enya Marie Catherine Alcindor, Benjamin Lusk, Vijaya Pandey, Adam D Longhurst, Emma Bolech, Becky Xu Hua Fu, Luke A Gilbert, James A Wohlschlegel, David P Toczyski

**Affiliations:** 1 Helen Diller Family Comprehensive Cancer Center, University of California, San Francisco, San Francisco, CA USA; 2 Department of Biochemistry and Biophysics, Helen Diller Family Comprehensive Cancer Center, University of California, San Francisco, San Francisco, CA USA; 3 Department of Biological and Biomedical Sciences, Harvard Medical School, Boston, MA USA; 4 Broad Institute of MIT and Harvard, Cambridge, MA USA; 5 Department of Biological Chemistry, David Geffen School of Medicine, University of California, Los Angeles, CA USA; 6 Arc Institute, Palo Alto, CA USA; 7 Acrobat Genomics, San Carlos, CA USA; 8 Department of Urology, University of California, San Francisco, San Francisco, CA USA

**Keywords:** Cancer, Post-translational Modifications & Proteolysis, Signal Transduction

## Abstract

FBXO42 is a poorly characterized F-box protein essential in 15% of cancer cell lines from diverse lineages. High-throughput approaches indicate that FBXO42 function correlates with that of coiled-coil protein CCDC6, and that the two proteins interact physically, but the relationship between them is not understood. We show that FBXO42 ubiquitinates PPP4C (protein phosphatase 4 catalytic subunit) and negatively regulates its expression. FBXO42 binds PPP4C independently of CCDC6. Similarly, we show that CCDC6 physically interacts with PPP4C independently of FBXO42, and identify a PPP4C binding site within CCDC6. However, mutation of CCDC6 does not reduce PPP4C ubiquitination, suggesting that FBXO42 and CCDC6 bind and regulate PPP4C through separate mechanisms. Viability of an FBXO42- and CCDC6-dependent glioblastoma cell line is rescued when PPP4C is knocked down along with FBXO42 or CCDC6, suggesting that aberrant activation of PPP4C is a driver of cell death when FBXO42 or CCDC6 activity is compromised. These findings tie together previous data suggesting that FBXO42 functions with CCDC6 by providing mechanistic insight into their independent regulation of PP4.

## Synopsis

The E3 ubiquitin ligase FBXO42 and the coiled-coiled domain-containing protein CCDC6 both negatively regulate PPP4C, and this target is critical for their essential role in some tumor cell lines. Despite this common function, FBXO42 and CCDC6 bind and regulate PPP4C through separate mechanisms.

- FBXO42 ubiquitinates PPP4C, negatively regulating its expression. CCDC6 binds to and negatively regulates PPP4C independently of FBXO42.
- In a cancer cell line where FBXO42 and CCDC6 are essential, aberrant PPP4C activity contribute to cell death following FBXO42 or CCDC6 depletion.
- CCDC6 binds to PPP4C through a repeated EXXXN motif.

## Abstract (web summary)

The E3 ubiquitin ligase FBXO42 and the coiled-coiled domain-containing protein CCDC6 both negatively regulate PPP4C, and this target is critical for their essential role in some tumor cell lines. Despite this common function, FBXO42 and CCDC6 bind and regulate PPP4C through separate mechanisms.

## Graphical abstract

**Figure:**
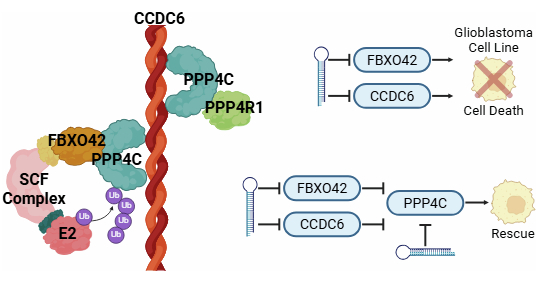

**Figure:**
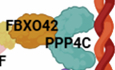

## Introduction

FBXO42 is a substrate receptor for the SCF, an E3 ubiquitin ligase composed of the RING finger protein RBX1, the cullin CUL1, and an adaptor protein SKP1, which binds F-box proteins that serve as substrate receptors (Bai et al, 1996; Bennett et al, 2010; Craig and Tyers, 1999; Deshaies and Joazeiro, 2009; Frescas and Pagano, 2008; Grim et al, 2008; Jin et al, 2004; Kipreos and Pagano, 2000; Liwocha et al, 2024; Rape et al, 2006; Skaar et al, 2009; Skowyra et al, 1997; Tyers and Willems, 1999; Zheng et al, 2002). FBXO42 is essential in approximately 15% of cancer cell lines, including cancer types from diverse lineages, but is not essential in non-cancerous cells such as RPE1 (Hoellerbauer et al, 2024; Toledo et al, 2015; Tsherniak et al, 2017; Zhou et al, 2024).

It was previously shown that FBXO42 is important for mitosis and correct assembly of the mitotic spindle, although the mechanism of this regulation is unknown (Hoellerbauer et al, 2024; Hundley et al, 2021). FBXO42 has also been implicated in acquired resistance to MEK1/2 inhibitors (Nagler et al, 2020); Notch signaling (Jiang et al, 2022); correct synaptonemal complex assembly and the unfolded protein response in Drosophila (Barbosa et al, 2020; Santos et al, 2025). In a study of neuroblastoma cells, it was found that depletion of FBXO42 led to cell cycle arrest and death via activation of p53 (Zhou et al, 2024). Conversely, in a study of glioblastoma cell lines where FBXO42 is essential, it was found that knockout of p53 was not sufficient to rescue arrest and death of FBXO42 knockout cells (Hoellerbauer et al, 2024). This suggests that FBXO42 knockout causes cell death or arrest, at least partially, through p53-independent mechanisms. However, none of these results explain the essential role of FBXO42 in a subset of cancer cell lines.

The DepMap cancer dependency database (https://depmap.org/portal) shows that FBXO42 has a very high co-dependency correlation with the coiled-coil domain protein CCDC6 (Tsherniak et al, 2017). FBXO42 and CCDC6 have been suggested to function together in regulating USP28 and p53 (Lü et al, 2024), and they physically interact based on high-throughput proteomics analyses (Huttlin et al, 2021; Huttlin et al, 2017; Oughtred et al, 2021). CCDC6 plays a role in the DNA damage response (Morra et al, 2022), and commonly forms an oncogenic fusion protein with RET (Cerrato et al, 2018; Li et al, 2019). However, the function of CCDC6 is not well-understood.

In this study, we investigated the function of FBXO42 and CCDC6 in cancer cell survival. We identified PPP4C, the catalytic subunit of protein phosphatase 4 (PP4), as a ubiquitination substrate of FBXO42. PP4 is involved in numerous aspects of cellular physiology which have been previously linked to FBXO42, including mitotic spindle assembly, p53 signaling, and DNA damage repair (Martin-Granados et al, 2008; Park and Lee, 2020; Shaltiel et al, 2014; Toyo-oka et al, 2008; Voss et al, 2013). We show that knockdown of PPP4C partially rescued cell death/arrest caused by FBXO42 or CCDC6 knockdown in the glioblastoma cell line LN-18, suggesting that PPP4C is the critical target of these proteins. Our data suggest that CCDC6 and FBXO42 downregulate PPP4C activity via distinct mechanisms, since knockdown of CCDC6 or FBXO42 negatively affects growth in cells in which the other has already been deleted. We also identify binding sites and critical residues in CCDC6 that participate in PPP4C interactions. In total, this study shows that FBXO42 and CCDC6 both act independently to inhibit PPP4C, and that this is important for survival in a subset of tumor cell lines.

## Results and discussion

### Immunoprecipitation-mass spectrometry based proteomics identifies FBXO42 interactors

Multiple recent studies have highlighted the importance of FBXO42 in mitosis and cancer (Barbosa et al, 2020; Hoellerbauer et al, 2024; Hundley et al, 2021; Jiang et al, 2022; Lü et al, 2024; Nagler et al, 2020; Zhou et al, 2024). However, a detailed understanding of its function, substrates, and contribution to cancer cell survival was lacking. Furthermore, the literature suggests that FBXO42 may work with CCDC6 in some fashion (Hoellerbauer et al, 2024; Lü et al, 2024), but the nature and function of their relationship were not well-understood.

**Figure 1:**
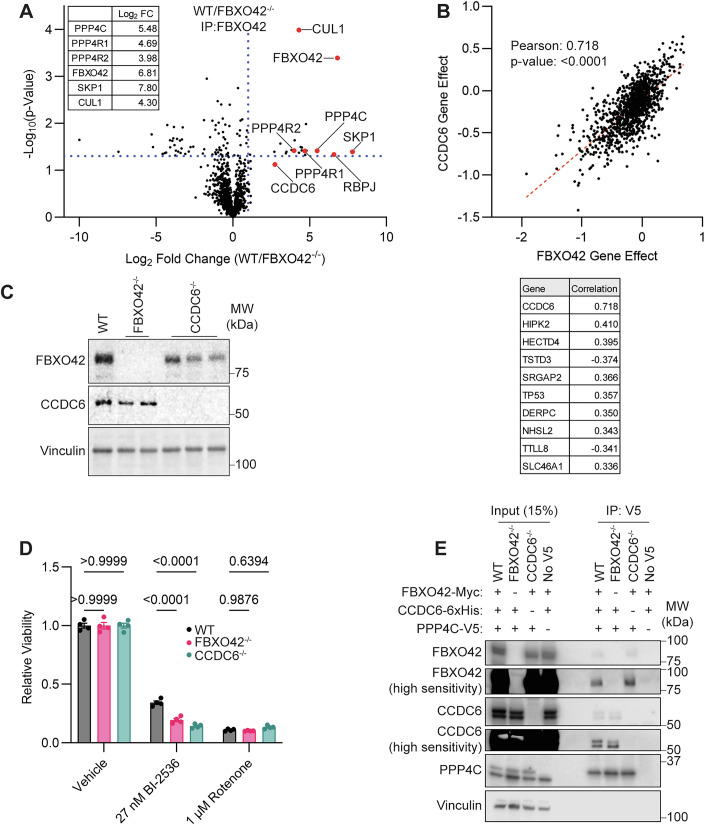
FBXO42 and CCDC6 bind PPP4C. (**A**) Immunoprecipitation-mass spectrometry (IP-MS) analysis of proteins interacting with anti-FBXO42 antibodies in HAP1 cells. Enrichment of proteins in WT cells compared to FBXO42^−/−^ cells is shown. Fold change values for PP4 complex and SCF E3 ubiquitin ligase complex members are in the inset table. Two biological replicates for each condition. Mann–Whitney test. (**B**) DepMap data comparing the CRISPR essentiality scores of FBXO42 and CCDC6 in the cell lines in the DepMap database. Top: Each dot is a cell line in the DepMap database. Pearson correlation coefficient and *P* value are displayed. Bottom: Table shows top 10 co-dependencies with Pearson correlations from DepMap Public 25Q3. (**C**) Western blots of clonal RPE1 cell lines showing expression of FBXO42 and CCDC6. (**D**) Presto Blue-based viability assays of clonal RPE1 cell lines with the indicated genotypes. Cells were treated with BI-2536 (PLK1 inhibitor, 27 nM) or rotenone (electron transport chain inhibitor, 1 µM). Each point represents a well of cells cultured in parallel. *N* = 4 for each condition, bars show mean ± SEM. Two-way ANOVA. *P* value of individual comparisons, with Dunnett’s multiple hypothesis test, is displayed. Representative of two independent experiments. (**E**) Co-immunoprecipitation (IP) western blotting performed on lysates from RPE1 cells. WT cells express FBXO42-Myc and CCDC6-6xHis, and PPP4C-V5 (except in the “No V5” lane). FBXO42^−/−^ cells express CCDC6-6xHis and PPP4C-V5. CCDC6^−/−^ express FBXO42-Myc and PPP4C-V5. Immunoblots were performed with antibodies specific to the proteins indicated. 15% input compared to IP or immunoprecipitation with a V5 epitope tag targeting antibody is shown. High sensitivity panels show exposures of the same membranes developed with high sensitivity ECL reagent. Source data are available online for this figure.

**Figure EV1:**
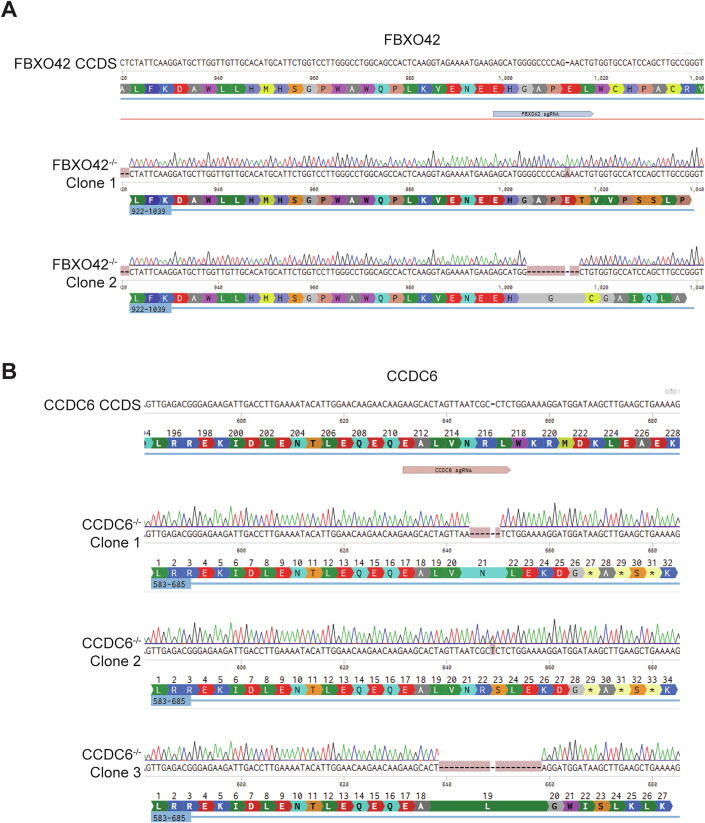
Sequences of FBXO42 and CCDC6 knockout clones. Sanger sequencing results are shown for (**A**). FBXO42 and (**B**). CCDC6 knockout clones, RPE1 cells. Consensus coding sequences (CCDS) are shown for each gene, as is the expected binding site of the sgRNA used to generate knockouts. WT and mutated sequences are aligned with amino acid translations. Each knockout cell line carries a mutation generating a frame shift.

Since FBXO42 is a SCF E3 ligase substrate adaptor, it is likely that its functions in cell survival and mitosis are mediated by a substrate that FBXO42 binds and ubiquitinates. To identify substrates of FBXO42, we performed immunoprecipitation of endogenous FBXO42 on lysates from HAP1 cells followed by mass spectrometry proteomics (IP-MS) (Fig. 1A). Wild-type cells were compared with FBXO42^−/−^ cells. As expected, cullin-RING ligase complex members CUL1 and SKP1, and the suggested substrate RBPJ (Jiang et al, 2022), were identified as FBXO42 interactors. We identified protein phosphatase complex 4 (PP4) members PPP4C, PPP4R1, and PPP4R2 (Fig. 1A), as seen in previously published high-throughput immunoprecipitation mass spectrometry analysis (Huttlin et al, 2021; Jiang et al, 2022; Oughtred et al, 2021). CCDC6 was also identified as an FBXO42 interactor, consistent with previous reports (Huttlin et al, 2021; Huttlin et al, 2017). CCDC6 exhibits a high co-dependency correlation with FBXO42 in terms of cell line essentiality (Fig. 1B), which is commonly observed for genes which serve a similar cellular function or are in the same pathway. Both FBXO42^−/−^ and CCDC6^−/−^ RPE1 cells (Figs. 1C and EV1) had increased sensitivity to the PLK1 inhibitor BI-2536 compared to WT cells (Fig. 1D), consistent with previous results for FBXO42 in HAP1 cells (Hundley et al, 2021). In contrast, they did not differ from wild-type cells in sensitivity to rotenone, which kills cells via an unrelated mechanism. The increased sensitivity of CCDC6^−/−^ cells is consistent with the hypothesis that FBXO42 and CCDC6 play similar functional roles in cells.

We analyzed FBXO42-CCDC6-PPP4C interactions by co-immunoprecipitation western blot in RPE1 cells expressing epitope-tagged alleles of PPP4C, FBXO42, and CCDC6. We immunoprecipitated V5-tagged PPP4C and observed that PPP4C interacts with both FBXO42 and CCDC6 (Fig. 1E). PPP4C-FBXO42 interactions were maintained in CCDC6^−/−^ cells, and PPP4C-CCDC6 interactions were maintained in FBXO42^−/−^ cells (Fig. 1E). This indicates that FBXO42 and CCDC6 are each able to bind PPP4C individually, independently of the complex.

**Figure EV2:**
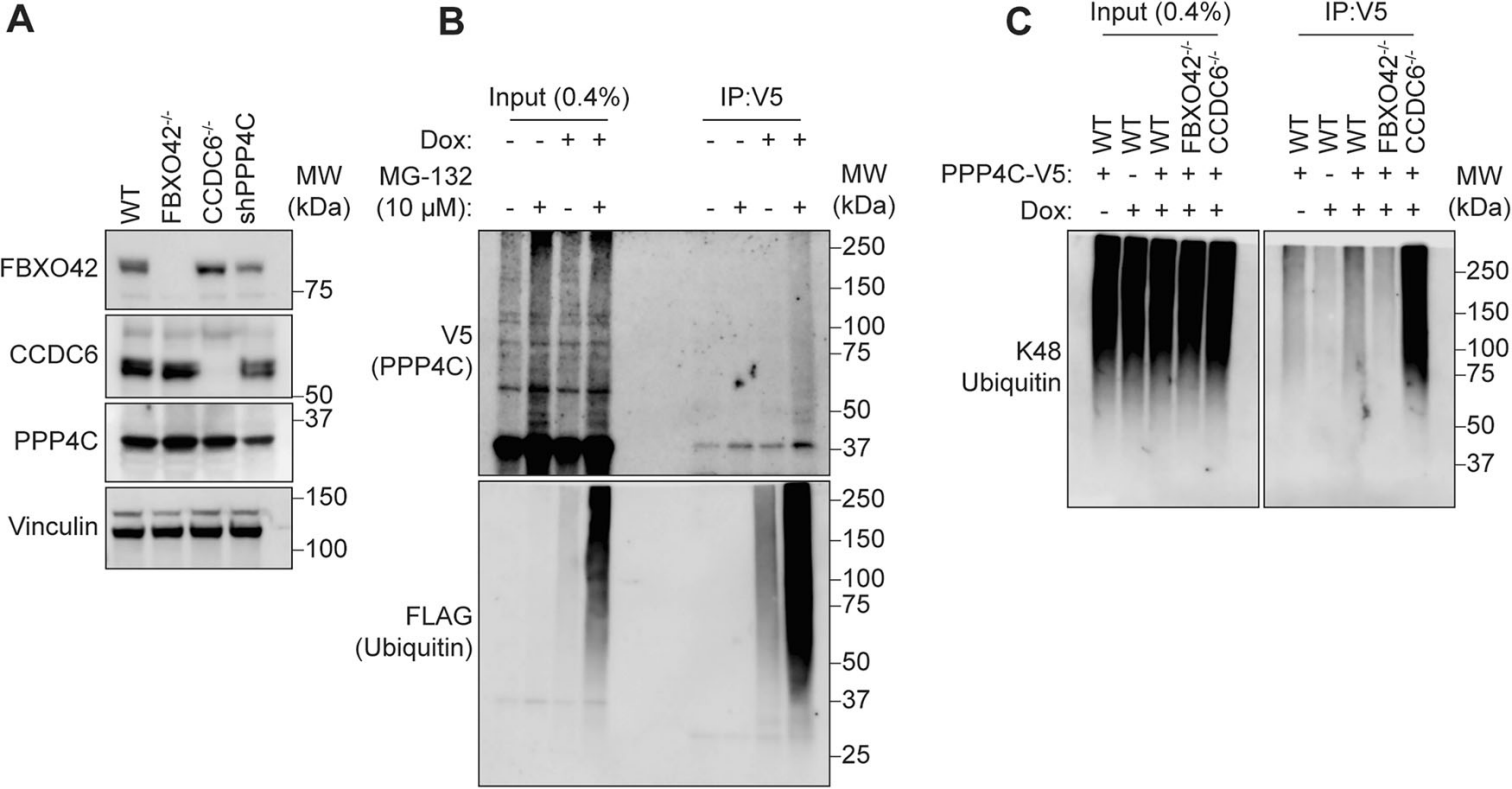
Interactions of FBXO42, CCDC6, and PPP4C continued. (**A**) Western blot showing expression of FBXO42, CCDC6, PPP4C, or vinculin (loading control) in the RPE1 cell lines used in Fig. 2A–C. (**B**) Denatured immunoprecipitation western blot performed as in Fig. 3B. FLAG-Ubiquitin expression was induced by treatment with 2 µg/ml doxycycline (Dox) for 24 h where indicated. Cells were treated with MG-132 at 10 µM for 4 h pre-harvest where indicated. (**C**) Western blot with an antibody specific for K48 linked ubiquitin. Same experiment as Fig. 3D.

**Figure 2:**
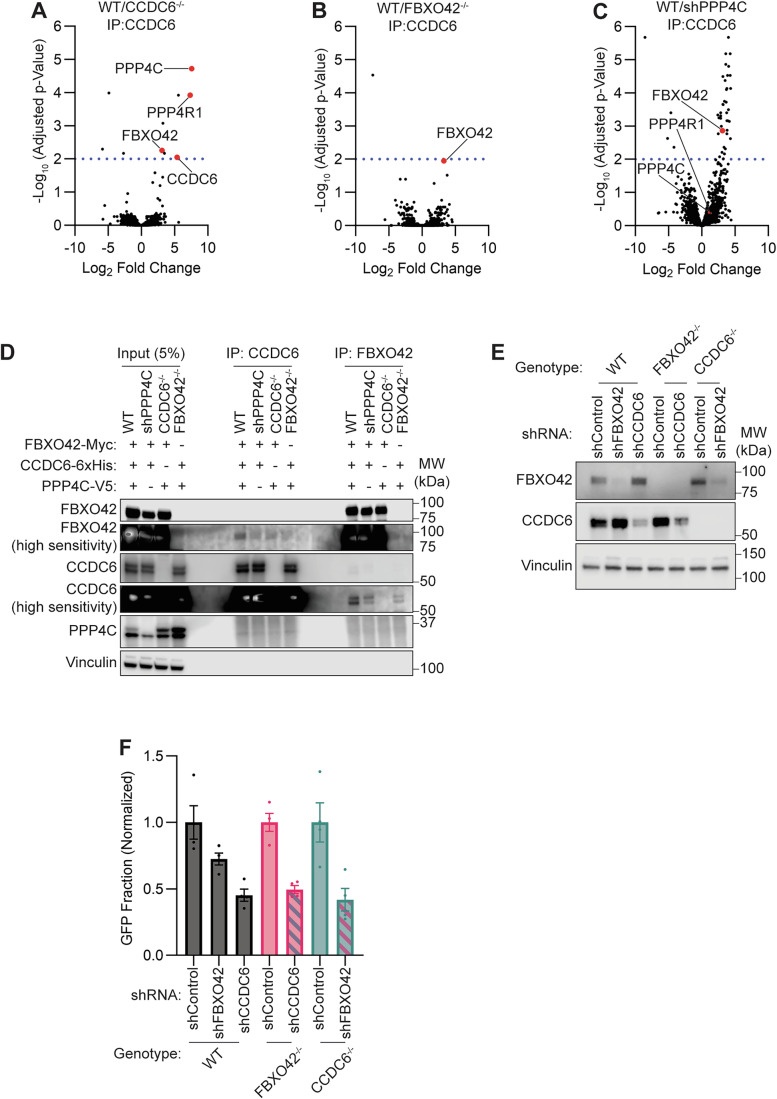
FBXO42 and CCDC6 bind PPP4C independently. (**A**–**C**) IP-MS analysis. Lysates from RPE1 cells were immunoprecipitated with an anti-CCDC6 antibody. Comparison of lysates from WT cells to (**A**). CCDC6^−/−^ cells. (**B**) FBXO42^−/−^ cells. (**C**) PPP4C knockdown cells. PPP4C was knocked down by lentivirus transduction with two shRNAs targeting PPP4C 3 days before cells were harvested. Proteins are expressed at endogenous levels. Two biological replicates, Mann–Whitney test. (**D**) Co-IP western blot. Similar to Fig. 1E, WT cells express FBXO42-Myc and CCDC6-6xHis, and PPP4C-V5 (except in the “No V5” lane). FBXO42^−/−^ cells express FBXO42-Myc and PPP4C-V5. CCDC6^−/−^ express FBXO42-Myc and PPP4C-V5. PPP4C is knocked down using shRNA. Immunoprecipitation of CCDC6 or FBXO42 performed with antibodies against those proteins (not epitope tags). 5% input compared to IP. High sensitivity panels show exposures of the same membranes developed with high sensitivity ECL reagent. Representative of three independent experiments. (**E**) Western blot showing expression of FBXO42 and CCDC6 in RPE1 cells. Clonal cell lines that are either WT, FBXO42^−/−^, or CCDC6^−/−^, were transduced with shRNA constructs targeting FBXO42, CCDC6, or a scramble control. Vinculin is included as a loading control. These cells are used in (**F**). (**F**) GFP, flow cytometry based competitive growth assay comparing the growth of RPE1 cells with FBXO42 and CCDC6 depleted and controls, in various combinations. All shRNA constructs express GFP as a marker. Growth competition was with WT RPE1 cells, which did not express GFP. Analyzed after 12 days of competitive growth. The fraction of GFP+ cells was measured and normalized to the fraction of GFP+ cells at time zero. Within each genotype (WT, FBXO42^−/−^, CCDC6^−/−^) the shFBXO42 and shCCDC6 conditions are normalized to the shScramble control. Each point represents replicate cells cultured in parallel. *N* = 4, bars represent mean ± SEM. Representative of three independent experiments. Source data are available online for this figure.

Since both FBXO42 and CCDC6 bound PPP4C, we explored whether the FBXO42-CCDC6 interaction was mediated by their simultaneous binding to PPP4C. We performed IP-MS analysis, using an antibody against endogenous CCDC6, with lysates from RPE1 cells that were wild-type, FBXO42^−/−^, CCDC6^−/−^, or had PPP4C knocked down using simultaneous transduction of two shRNAs (shPPP4C) (Fig. EV2A). Complete knockouts of PPP4C could not be generated because PPP4C is essential in most, if not all, cells. Comparing WT cells to CCDC6^−/−^ control cells (Fig. 2A), we observed enrichment of PPP4C, PPP4R1, and FBXO42, consistent with CCDC6 interacting with these proteins. Comparing FBXO42^−/−^ cells to WT cells (Fig. 2B), there was no significant change in CCDC6 interactions other than with FBXO42 itself, consistent with PPP4C co-IPing with CCDC6 in both WT and FBXO42^−/−^ cells. This confirmed that FBXO42 has little or no effect on CCDC6 interactions with the PP4 complex or any other proteins (Fig. 2B). Comparing WT cells to shPPP4C cells, we observed that FBXO42 was enriched 8.63-fold (*P* value 1.38 × 10^−3^) in WT cells over the shPPP4C cells, indicating that CCDC6-FBXO42 interactions were decreased when the levels of PPP4C were reduced (Fig. 2C). We then examined whether the FBXO42-CCDC6 interaction depended upon PPP4C using co-immunoprecipitation western blotting. We immunoprecipitated either FBXO42 or CCDC6, and saw that in both cases, knockdown of PPP4C decreased the FBXO42-CCDC6 co-immunoprecipitation to background levels (Fig. 2D). This, combined with the finding that FBXO42 and CCDC6 bind PPP4C independently of each other (Fig. 1E), suggests that FBXO42 and CCDC6 may not bind directly to each other, but rather co-immunoprecipitate because they both bind to PPP4C.

It has been proposed that FBXO42 is an upstream regulator of CCDC6 (Lü et al, 2024). To investigate the relationship between FBXO42 and CCDC6, we knocked down CCDC6 using shRNA in FBXO42^−/−^ cells, and vice versa (Fig. 2E). We evaluated the growth rate of these cells using competitive growth assays. Knockdown of FBXO42 or CCDC6 reduced cell growth. Knockdown of CCDC6 in FBXO42^−/−^ cells further reduced cell growth rates, and vice versa (Fig. 2F). This shows that FBXO42 and CCDC6 have independent, non-overlapping functions. If the correlation between FBXO42 and CCDC6 (Fig. 1B) could be fully explained by FBXO42 acting as an upstream regulator of CCDC6 (or vice versa), then knockdown of CCDC6 in FBXO42^−/−^ cells should have minimal effects on cell growth rate because the knockout of FBXO42 and knockdown of CCDC6 would be functionally redundant.

### FBXO42 is required for PPP4C ubiquitination

**Figure 3:**
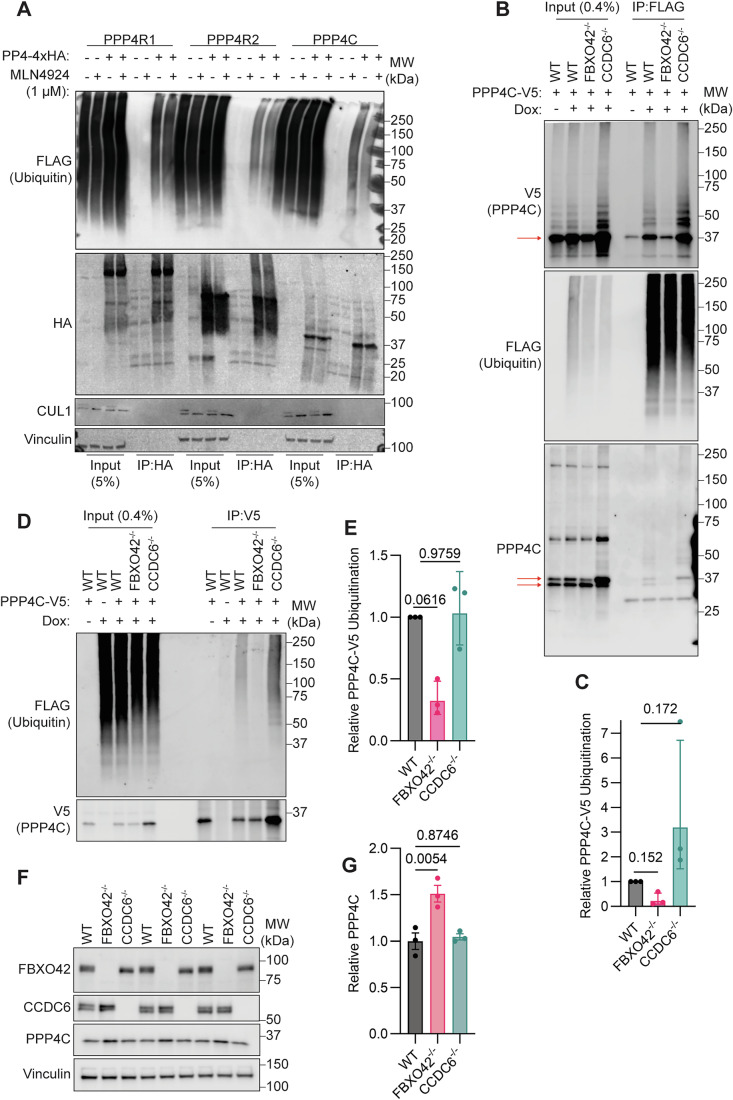
FBXO42 ubiquitinates PPP4C and suppresses its expression. (**A**) IP western blotting of lysates from RPE1 cells. All cells express FLAG-tagged ubiquitin from a doxycycline (dox) induced promoter, induced with 1 µg/ml dox 24 h before harvest. MLN4924 (pan-cullin-RING ligase inhibitor) added at 1 µM to indicated cells 24 h before harvest. In total, 10 µM MG-132 was added to cells 4 h before harvest. Cells express PPP4R1-4xHA, PPP4R2-4xHA, or PPP4C-4xHA as indicated. 5% Input compared to IP. IP was performed with beads with cross-linked anti-HA antibodies. CUL1 blot shows double bands in vehicle treated and a single band in MLN4924 treated lanes, consistent with inhibition of CUL1 neddylation. (**B**) IP western blotting of lysates from RPE1 cells. All cells express FLAG-tagged ubiquitin from a dox induced promoter, induced with 2 µg/ml dox 24 h before harvest where indicated. All cells express PPP4C-V5. All cells were treated with MG-132 at 10 µM for 4 h before harvest. Protein lysates were denatured before immunoprecipitation to remove non-covalent interactions. 0.4% input compared to IP. IP was performed with anti-FLAG antibodies crosslinked to beads. Red arrows indicate the bands corresponding to the molecular weight of PPP4C. Note in the PPP4C blot two bands can be seen for PPP4C, the higher one corresponding to the V5 tagged version, the lower one corresponding to endogenously expressed PPP4C. Representative of three independent experiments. (**C**) Quantification of the polyubiquitin smears in the V5 blot, elution from (**B**). Each point is a separate, replicate experiment and western blot. Values are normalized to the PPP4C-V5 expression level (input) and the total amount of immunoprecipitated FLAG-Ubiquitin (elution) for each sample. *P* values shown are from a one-way ANOVA performed on log_2_-transformed fold change values, adjusted for repeated measures, and with Dunnett’s multiple hypothesis test. *N* = 3, biological replicates, bars represent geometric mean ± geometric SD. Values normalized to WT. See “Methods” for more normalization details. (**D**) IP western blot of lysates from RPE1 cells. All cells express FLAG-tagged ubiquitin from a dox induced promoter, induced with 2 µg/ml dox 24 h before harvest. In total, 10 µM MG-132 was added to cells 4 h before harvest. Protein lysates were denatured before immunoprecipitation to remove non-covalent interactions. Indicated cells express V5-tagged PPP4C. 0.4% Input compared to IP. IP was performed with a V5 antibody crosslinked to beads. Representative of three independent experiments. (**E**) Quantification of FLAG-tagged polyubiquitin smears from Fig. 3D. Each point is a separate, replicate experiment and western blot. Ubiquitin smear signal was normalized to the amount of V5 eluted, and to the FLAG input. *P* values shown are from a one-way ANOVA performed on log_2_-transformed fold change values, adjusted for repeated measures, and with Dunnett’s multiple hypothesis test. *N* = 3, biological replicates, bars represent geometric mean ±  geometric SD. Values normalized to WT. See “Methods” for more normalization details. (**F**) Western blot showing PPP4C levels in WT, FBXO42^−/−^, or CCDC6^−/−^ RPE1 cells. Three replicates are cells collected independently at three different passages. (**G**) Quantification of (**F**). Three replicates are cells collected independently at three different passages. *N* = 3, biological replicates, bars represent geometric mean ± SEM. PPP4C expression normalized to vinculin and then to the WT control. One-way ANOVA, with Dunnett’s multiple hypothesis test. Source data are available online for this figure.

To determine if FBXO42 ubiquitinates PP4 subunits, we generated a doxycycline (dox) induced, FLAG-tagged ubiquitin construct, and 4xHA-tagged PPP4C, PPP4R1, and PPP4R2 constructs and introduced these into RPE1 cells. While the levels of endogenous PPP4C are consistently elevated in FBXO42 mutants, we observed wide variance in the expression of heterologously expressed, V5-tagged PPP4C (Figs. 1–3). Experimental data was normalized to account for this in each relevant experiment, see Methods for normalization details. To examine PPP4C ubiquitination directly, we immunoprecipitated HA-tagged PP4 subunits and blotted for FLAG (ubiquitin) or HA. Cells were treated with a vehicle control or with MLN4924, which broadly inhibits cullin RING ligases (Baek et al, 2020; Brownell et al, 2010; Soucy et al, 2009). While significant polyubiquitination smears were observed for all three proteins, it appeared that the smear associated with PPP4C was slightly reduced with MLN4924 treatment (Fig. 3A). Since this suggested that PPP4C is ubiquitinated by a cullin RING ligase (CRL), we focused our investigation on the role of FBXO42 in PPP4C ubiquitination. However, further investigation is warranted into ubiquitination of PPP4R1 and PPP4R2, by CRLs or other E3 ligases. We immunoprecipitated FLAG-tagged ubiquitin and blotted for V5-tagged PPP4C and observed a 2.9-fold reduction in ubiquitinated PPP4C in FBXO42^−/−^ cells, even when normalized to PPP4C expression and FLAG-Ubiquitin pull-down efficiency (Fig. 3B,C).

We performed the reciprocal experiment and immunoprecipitated V5-tagged PPP4C and blotted for FLAG-Ubiquitin. PPP4C showed a high-molecular weight smear, typical of ubiquitinated proteins. Consistent with the FLAG-ubiquitin immunoprecipitation, FBXO42^−/−^ cells displayed reduced ubiquitination of PPP4C (Fig. 3D,E). In contrast, mutation of CCDC6 did not decrease PPP4C ubiquitination. The ubiquitination smear was only detectable in cells treated with MG-132, a proteasome inhibitor (Fig. EV2B). This suggests that the ubiquitination of PPP4C is degradative. Using linkage-specific antibodies, we found that K48 ubiquitin linkages, typically a degradative ubiquitination signal, were enriched when V5-tagged PPP4C was immunoprecipitated (Fig. EV2C). We observed that PPP4C levels were consistently elevated in FBXO42^−/−^ cells, but not CCDC6^−/−^ cells (Fig. 3F,G). Notably, this difference was consistently observed for endogenously expressed PPP4C, but was not for overexpressed tagged PPP4C, which varies in levels (Figs. 1E, 2D and 3B,D). These findings are consistent with the hypothesis that FBXO42 ubiquitinates PPP4C, leading to its degradation. PPP4C is not a highly unstable protein (Mathieson et al, 2018), suggesting that FBXO42 may only target a subset of it, or may target bulk levels in a subset of cells.

### Knockdown of PPP4C rescues sensitivity to FBXO42/CCDC6 knockdown in a glioblastoma cell line

**Figure 4:**
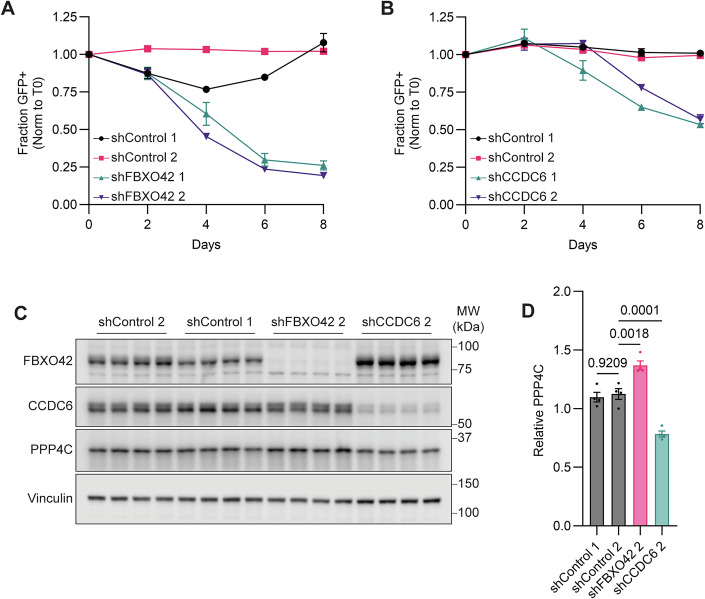
Depletion of FBXO42 or CCDC6 causes proliferation deficits in a glioblastoma cell line. (**A**) GFP, flow cytometry-based, competitive growth assay. LN-18 cells transduced using lentivirus with shRNA constructs against non-targeting sequences, or FBXO42. Each point represents a replicate well of cells cultured in parallel. *N* = 2, each point represents the midpoint, bars illustrate the range of the two replicates (not an error bar). Representative of three independent experiments. (**B**) As (**A**), but with shRNAs targeting CCDC6. (**C**) Western blot showing expression of indicated proteins in LN-18 cells transduced with a scramble control, or shRNA against FBXO42 or CCDC6. Four replicate, parallel transductions were performed for each of the two control shRNAs, the anti-FBXO42 shRNA (the same construct as “shFBXO42 2” from Fig. 4A), and the anti-CCDC6 shRNA (the same construct as “shCCDC6 2” from Fig. 4B). Each lane for an shRNA is from one of the four replicate transductions. (**D**) Quantification of PPP4C protein levels from (**C**). PPP4C expression normalized to vinculin and then to the shControl 1 control. One-way ANOVA, with Dunnett’s multiple hypothesis test. *N* = 4, bars represent mean ± SEM. Each point represents cells from a separate, replicate transduction. Source data are available online for this figure.

**Figure EV3:**
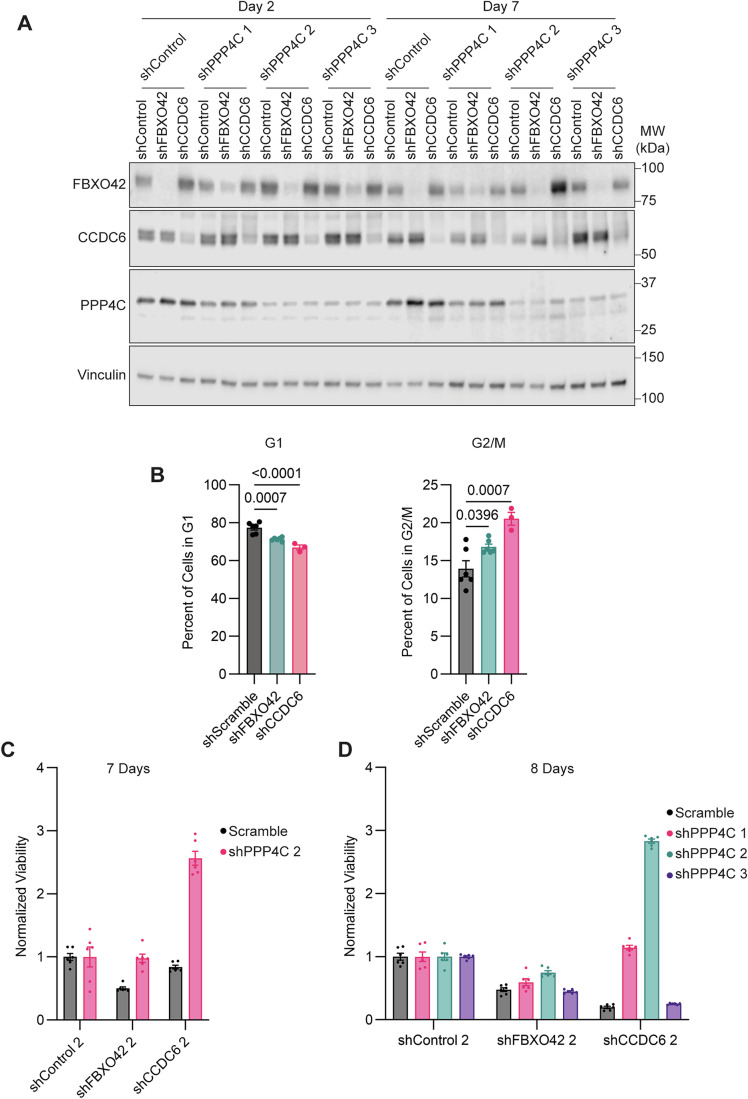
The effects of FBXO42 and CCDC6 on PPP4C cell cycle and proliferation in LN-18 cells. (**A**) Western blot analysis of lysates from LN-18 cells transduced with shRNA against PPP4C or a scramble control. Cells were also, simultaneously, transduced with shRNA targeting FBXO42, CCDC6, or a scramble control. Days post transduction are indicated. (**B**) Flow cytometry-based cell cycle analysis of LN-18 cells transduced with shRNA constructs targeting FBXO42, CCDC6, or a scramble control. Cells were transduced, selected by FACS sorting on GFP, and analyzed 10 days later. Propidium iodine staining was used to assess DNA content. Each point indicates replicate, parallel cell cultures. *N* = 6, bar represents mean ±  SEM. *P* values from two-way ANOVA with Dunnett’s multiple hypothesis test. (**C**) A replicate experiment performed similarly to that in Fig. 5A, except that only one anti-PPP4C shRNA (“shPPP4C.2”) was used and cells were analyzed at 7 days post transduction, not six. Each point indicates wells of replicate, parallel cell cultures. *N* = 6, bar represents mean ± SEM. (**D**) A replicate experiment performed similarly to that in Fig. 5A, except that cells were analyzed at 8 days post transduction instead of six. Each point indicates wells of replicate, parallel cell cultures. *N* = 6, bar represents mean ± SEM.

**Figure 5:**
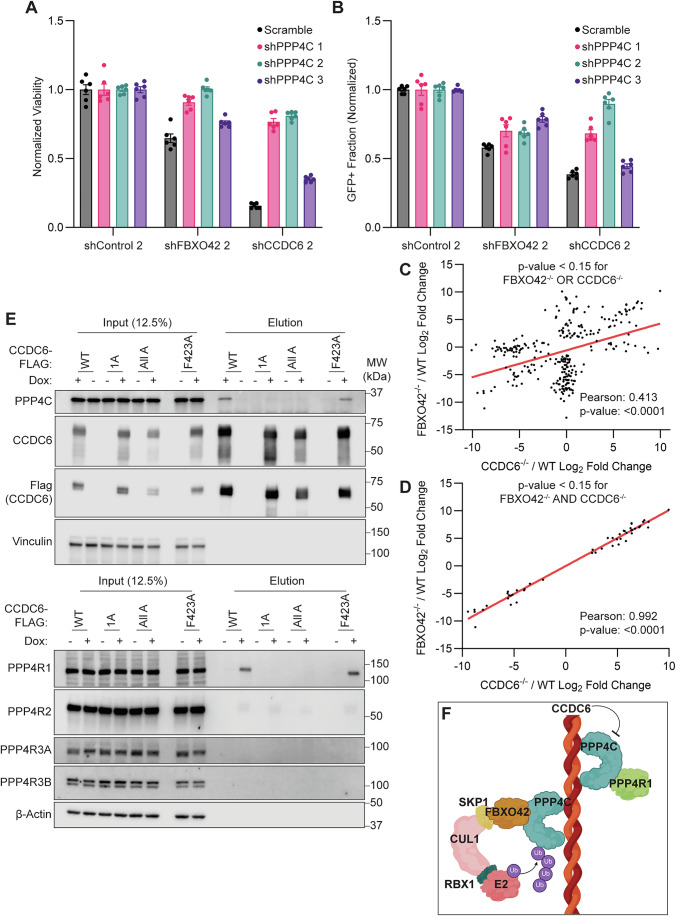
PPP4C knockdown rescues proliferation deficits in FBXO42 or CCDC6 depleted cells. (**A**) Presto Blue based viability assay. LN-18 cells were transduced with shRNA against FBXO42, CCDC6, or a scramble control using lentivirus. Cells were additionally transduced with shRNA against one of three shRNA sequences targeting PPP4C, or a scramble control. Equal numbers of cells were seeded, and viability was assessed 6 days after transduction. Viability was normalized to the scramble control paired with the FBXO42 or CCDC6 constructs. Each point represents a replicate well of cells cultured in parallel. *N* = 5, bar represents mean ± SEM. Additional replicates shown in Fig. EV3C,D. (**B**). GFP-based competitive growth assay analysis of LN-18 cells treated as in (**A**). Fraction of GFP-expressing cells are shown, normalized to time zero, and to the Scramble-GFP control. Analyzed on day 6 post-transduction. Representative of 3 independent experiments. *N* = 5, bar represents mean ±  SEM. (**C**) Phosphoproteomics results. RPE1 cells, *x* axis is CCDC6^−/−^ cells compared to WT cells, *y* axis is FBXO42^−/−^ cells compared to WT cells. Phosphopeptides with an adjusted *P* value below 0.15 for either comparisons are shown. Linear regression line is shown. See methods for analysis and statistics details. Each point is a phosphopeptide. Analysis performed from two biological replicates. Pearson’s correlation coefficient and *P* value are shown. (**D**) As (**C**), but only phosphopeptides with an adjusted *P* value < 0.15 for both comparisons are shown and used for linear regression and correlation analysis. (**E**) Co-immunoprecipitation western blot. In-cell test of Alphafold predictions shown in Fig. EV4A–G. RPE1 cells, all with endogenous CCDC6 knocked out. Dox induced, 3xFLAG-His tagged CCDC6 constructs were stably introduced. WT is wild-type CCDC6. “1 A” and “All A” have mutations to residues in the predicted PPP4C-CCDC6 binding sites that have a full or partial version of the motif EXXXNXXXK. “1A” has the asparagine residue of each of the four binding sites mutates to an alanine. “All A” has every residue in the motif mutated to alanine for all four potential binding sites. F423A has just that one residue mutated. PPP4C and PPP4R1 are endogenously expressed. Cells were treated with 2 µg/ml dox where indicated. Lysates were immunoprecipitated using an anti-FLAG antibody crosslinked to beads. Elutions are compared with 12.5% input. Representative of three separate, replicate experiments. (**F**) Schematic representing our hypothesized model of FBXO42-PPP4C-CCDC6 interaction. FBXO42 acts as a substrate adapter for PPP4C ubiquitination. Separately, CCDC6 binds PPP4C and PPP4R1. Source data are available online for this figure.

We hypothesized that FBXO42 and CCDC6 negatively regulate the activity of PP4, and that the loss of this regulation is responsible for their essentiality in some cell lines, such as the glioblastoma line LN-18. CCDC6 has been previously reported to directly interact with PP4 and inhibit its activity, including in direct, in-vitro phosphatase assays (Merolla et al, 2012). We analyzed the viability and growth of LN-18 cells transduced with shRNA against FBXO42 or CCDC6 using a flow-cytometry-based competitive growth assay. As expected, we observed that FBXO42 or CCDC6 knockdown reduced cell fitness compared to scramble controls (Fig. 4A,B). We analyzed the cell cycle position of freely cycling LN-18 cells with FBXO42 or CCDC6 knocked down, and observed that in both cases, G2/M phases were increased and G1 was decreased (Fig. EV3B). This may indicate that loss of FBXO42 or CCDC6 in LN-18 cells causes arrested or delayed mitosis, consistent with observations in other cell types (Hoellerbauer et al, 2024; Hundley et al, 2021). Knockdown of FBXO42 increased protein levels of PPP4C (Figs. 4C,D and EV3A), as we had observed in RPE1 cells. To test if increased levels or activity of PPP4C contributed to the reduced proliferation of FBXO42 or CCDC6 knockdown cells, we simultaneously knocked down PPP4C and either FBXO42 or CCDC6 (Fig. EV3A). The shRNA against FBXO42, CCDC6, or scramble control were expressed in a vector with a GFP marker, and each of the three anti-PPP4C shRNA, and their scramble control, were expressed in a GFP-negative vector with a puromycin resistance marker (Puro). We were unable to generate stable PPP4C knockout clones, since it is an essential gene. Cell viability was measured using Presto Blue. Growth for the Puro vector shRNAs were normalized to that of cells with the GFP scramble control (Fig. 5A). In parallel experiments, we compared the growth of the FBXO42 or CCDC6 knockdown with or without PPP4C knockdown in a competitive growth assay (Fig. 5B). In both assays, we found that knockdown of PPP4C partially rescued the effects of either FBXO42 or CCDC6 knockdown, suggesting that these proteins negatively regulate PP4 activity in cells. Data from additional replicates are provided in Fig. EV3C,D. Given that overactivation of PP4 can inhibit cell proliferation (Huang et al, 2016), we hypothesize that overactive PP4 causes cell death in FBXO42 and CCDC6 knockout cells. Cell lines such as LN-18 may be selectively dependent upon FBXO42 and CCDC6 because they have intrinsic mitotic defects or increased reliance on a kinase opposed by PP4. Interestingly, knockdown of PPP4C was observed to slightly increase FBXO42 expression (Fig. EV3A). The possibility that partial rescue of FBXO42 expression contributes to rescued viability in cells with both FBXO42 and PPP4C knocked down (Fig. 5A,B) cannot be excluded. These findings mirror those from a recent study that found that depletion of PP4 rescued another FBXO42 depletion-associated phenotype, in this case sensitivity to DNA damage (Yang et al, 2026). This study was performed in cells where FBXO42 was depleted at the genetic level, making rescue of the phenotype by increased FBXO42 expression unlikely.

We performed mass-spectrometry-based phosphoproteomics analysis of lysates from WT, FBXO42^−/−^, and CCDC6^−/−^ RPE1 cells. When FBXO42^−/−^ and CCDC6^−/−^ were each compared to WT cells, we found a high degree of correlation in the differentially phosphorylated peptides between the two conditions (Pearson’s correlation 0.5037, *P* value < 0.0001) (Fig. 5C,D; Dataset EV1). This indicates that FBXO42 and CCDC6 have a high degree of overlap in their regulation of the phospho-proteome. Phosphopeptides were identified that were de-enriched/enriched in either just FBXO42^−/−^ (clustered on the *y* axis), just CCDC6^−/−^ cells (clustered on the *x* axis), or both (on the positive diagonal) (Fig. 5C). When filtering for phosphopeptides that have statistically significant (*P* value <0.15) values in both FBXO42^−/−^ and CCDC6^−/−^ cells, a smaller subset of proteins is highlighted (Fig. 5D). Together, these data indicate that while FBXO42 and CCDC6 have some overlap in their downstream regulatory effects, their functions are not completely redundant. This is consistent with the finding that depletion of both proteins has an additive effect in inhibiting cell proliferation (Fig. 2F), and that FBXO42 appears to associate with both PPP4R1 and PPP4R2, whereas CCDC6 only associates with PPP4R1 (Figs. 1A and 5E). It is important to note that this phosphoproteomics analysis cannot distinguish direct effects of PP4 inhibition from downstream effects.

### CCDC6 residues mediating PPP4C interactions

CCDC6 binds to PP4 (Figs. 1E and 2A) and (Ueki et al, 2019), and reduces its activity (Morra et al, 2022), and we show that this interaction is independent of FBXO42. While CCDC6 has been reported to associate with PP4 through the PPP4R3 subunits (Ueki et al, 2019), we find that PPP4R1 (which forms heterodimers with PPP4C) co-immunoprecipitates with CCDC6 (Fig. 2A). Since PPP4R1 and PPP4R3 subunits are in separate PP4 complexes, CCDC6 must recognize the PP4 complexes by at least one PPP4R3-independent mechanism.

**Figure EV4:**
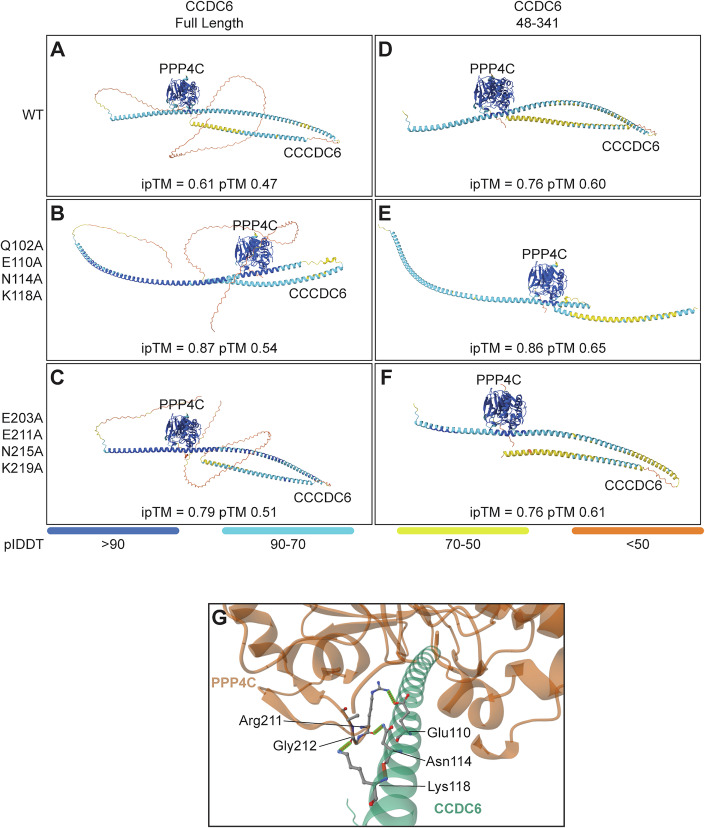
Alphafold predictions of possible CCDC6-PPP4C interactions. (**A**–**F**) Alphafold 3 predictions of CCDC6-PPP4C interactions. ipTM and pTM values shown. Protein representations colored by pIDDT values. (**A**–**C**) Uses full length CCDC6, (**D**–**F**) uses CCDC6 with its terminal disordered regions truncated, residues 48–341. (**A**,** D**) Use WT CCDC6. In (**B**, **E**, **C**,** F**), the indicated residues on CDC6 are mutated to alanine to ablate the two different predicted binding sites. Alphafold predictions are theoretical and should be interpreted carefully. (**G**) Alphafold prediction of PPP4C-CCDC6 binding. PPP4C is in orange, CCDC6 is in blue. Hydrogen bonds between the two peptides are highlighted. Amino acids predicted to participate in hydrogen bonding are indicated. Alphafold predictions are theoretical and should be interpreted carefully.

We investigated the mechanism of the CCDC6-PPP4C interactions. We used Alphafold 3 (Abramson et al, 2024; Jumper et al, 2021) to predict possible interactions between FBXO42, CCDC6, and PPP4C. While Alphafold was not able to predict a complex of PPP4C and FBXO42 with adequate confidence, it did predict an interaction between PPP4C and CCDC6 (Fig. EV4A–F). Alphafold identified that CCDC6 has two predicted binding sites for PPP4C with reasonably high confidence. The first predicted binding site showed hydrogen bonds between PPP4C and the CCDC6 residues Glu110, Asn114, Lys118 (Fig. EV4A,C,D,F,G). When these residues are mutated to alanine, Alphafold predicts a second binding site, with hydrogen bonds between PPP4C and CCDC6 residues, Glu211, Asn215, and Lys219 (Fig. EV4B,E). Notably, both binding sites contain the motif EXXXNXXXK. It is possible that this motif mediates PPP4C-CCDC6 interactions. CCDC6 has predicted disordered regions from residues 1–47 and most of 342–474 (the C-terminal) (Consortium, 2024). We repeated Alphafold predictions with just the CCDC6 residues 48–341 (Fig. EV4D–F). This generally increased the ipTM values of the predictions, and PPP4C still was predicted to interact with CCDC6 at the same residues.

In addition to these two EXXXNXXXK motifs in CCDC6, we identified two other sites with partial versions of this motif: E139 and N142, and E334 and N338. In Alphafold 3 predictions, mutation of the N residue to A consistently caused a major drop in ipTM. To test these predictions, we generated a Flag-tagged CCDC6 allele where only the asparagine residue of these four potential binding sites were all mutated to A (“1A” variant, N114A, N143A, N215A, and N338A). We also generated a CCDC6 allele where every residue predicted to be a part of these four binding sites was mutated to alanine (“All A”, Q102A, E110A, N114A, K118A, E139A, N143A, E203A, E211A, N215A, K219A, E326A, E334A, N338A). These constructs were expressed in RPE1 with endogenous CCDC6 knocked out. PPP4C was expressed at endogenous levels. Using a co-IP, we found that both CCDC6 mutants no longer bound PPP4C (Fig. 5E). We also expressed a variant of CCDC6 with the mutation F423A, which has been previously reported to disrupt CCDC6-PP4 interactions (Ueki et al, 2019). However, we did not observe a difference in the CCDC6-PPP4C interaction when this mutation was introduced. Moreover, we blotted for PPP4R1, the regulatory PP4 regulatory subunit we previously found to interact with CCDC6 via immunoprecipitation proteomics (Fig. 2A). We found that, similar to PPP4C, PPP4R1 bound WT and F423A CCDC6 but not the “1A” or “All A” mutants (Fig. 5E). We did not observe binding to PPP4R2, PPP4R3A, or PPP4R3B (Fig. 5E). This suggests that CCDC6 may specifically bind the PPP4C-PPP4R1 heterodimer, or may do so under certain cell states. Notably, this is distinct from the interactions of CCDC6 and PP4 described in (Ueki et al, 2019), which appears to be between CCDC6 and the PPP4C-PPP4R2-PPP4R3A/PPP4R3B hetero trimers. We did not detect interactions between CCDC6 and PPP4R2, PPP4R3A, or PPP4R3B (Figs. 2A and 5E), but we cannot rule them out.

In summary, we present data showing that CCDC6 and FBXO42 inhibit PPP4C in different ways: FBXO42 by targeting PPP4C for turnover, and CCDC6 by an as of yet not understood, but previously characterized, mechanism (Morra et al, 2022) (Fig. 5F). It has been reported that CCDC6 directly binds PPP4C and reduces its catalytic activity, but the exact means by which it does this is not understood (Merolla et al, 2012). Notably, according to proteomics studies in HeLa cells, CCDC6 and PPP4C are present in the cell in similar copy numbers, consistent with direct inhibition, whereas FBXO42 is about 100-fold less abundant, as expected for a catalytic role (Bekker-Jensen et al, 2017). It will be important for future studies to determine what portion of cellular PPP4C interacts with CCDC6 at a given point in time, what regulates this interaction, and with which PP4 holoenzymes CCDC6 interacts.

Significantly, a parallel study published concurrently with this one found that FBXO42 binds to and negatively regulates PPP4C (Yang et al, 2026). The authors of that study also found that FBXO42 ubiquitinates PPP4C, and that PPP4C levels are increased in FBXO42 depleted cells, but, in contrast to our conclusions, the authors conclude that ubiquitination of PPP4C is primarily non-degradative and serves to modulate PP4 catalytic activity (Yang et al, 2026).

The differential association with regulatory subunits, and the existence of some CCDC6- and FBXO42-specific alterations in the phosphoproteome both highlight that, while there is significant overlap in FBXO42 and CCDC6 function, they are not redundant and may be regulated through different mechanisms. For example, they could function in different cellular compartments, as reportedly FBXO42 is mostly nuclear, CCDC6 is cytoplasmic but travels to the nucleus, and PP4 localizes more to the nucleus but is clearly detected in the cytosol (Ueki et al, 2019; Uhlén et al, 2015; Wu et al, 2022). It is also possible that they bind different PP4 holoenzymes. We observed FBXO42 immunoprecipitating PPP4R2 and PPP4R1 (Fig. 1A), but CCDC6 only immunoprecipitating PPP4R1 (Figs. 2A and 5E). PPP4R2 is reported to be nuclear whereas PPP4R1 was shown to be cytoplasmic (Thul et al, 2017). Conversely, other studies have found CCDC6 interacting with PPP4R2, PPP4R3A, and PPP4R3B (Huttlin et al, 2021; Huttlin et al, 2017; Lü et al, 2024; Oughtred et al, 2021). It has been reported that CCDC6 translocates to the nucleus in response to stimuli such as DNA damage or serum starvation (Celetti et al, 2004; Merolla et al, 2007). Since these stimuli were not applied to the RPE1 cells we used to study CCDC6 interactions, it is possible that CCDC6 was largely cytoplasmic at the time and as a result associated more with PPP4C-PPP4R1.

FBXO42 has been shown to regulate synaptonemal complex assembly during meiosis in Drosophila by downregulating protein levels of PP2A-B56 (Barbosa et al, 2020). PP2A-B56 is a regulatory subunit of PP2A. PP2A and PP4 are closely related and evolutionarily conserved (Chen et al, 2017), so it is plausible that FBXO42 could regulate both complexes in both organisms. While reducing PPP4C expression partially rescued cell fitness in FBXO42 knockdown cells, this does not rule out the contribution of other FBXO42 substrates. Furthermore, it is unknown which PP4 substrates contribute to the phenotype of FBXO42^−/−^ cells, and whether it is the same substrate affecting mitosis. It has been shown that PP4 regulates microtubule organization via dephosphorylation of NDEL1 (Toyo-oka et al, 2008). We speculate that perturbation of this regulatory mechanism could account for the dysregulation of mitotic spindle assembly observed in FBXO42 knockout cells. Further study is also needed to elucidate the regulation of PPP4C targeting by FBXO42 and the mechanism of CCDC6 regulation of PP4. Answering these questions may reveal why FBXO42 and CCDC6 are essential in only a subset of cancer cells, which could guide the development of new, targeted cancer therapies (Fischer et al, 2014; Senft et al, 2018).

## Methods

**Table:** Reagents and tools table

Reagent/resource	Reference or source	Identifier or catalog number
**Experimental models**		
hTERT-RPE1	A gift from Jussi Taipale	RRID: CVCL_4388
LN-18		RRID: CVCL_0392
HAP1		RRID: CVCL_Y019
HEK293T		RRID: CVCL_0063
**Recombinant DNA**		
psPAX2	A gift from Didier Trono (from Addgene).	Addgene#12260
pMD2.G	A gift from Didier Trono (from Addgene).	Addgene#12259
pLKO.1-shFBXO42-3xFLAG-EGFP #1	This study	shRNA target sequence: CCATCAGTGTTATCATGGTTT
pLKO.1-shFBXO42-3xFLAG-EGFP #2	This study	shRNA target sequence: TAGATGATGCAACTATCTTAA
pLKO.1-shCCDC6-3xFLAG-EGFP #1	This study	shRNA target sequence: GAGTTCAAGCAGGCCTATATC
pLKO.1-shCCDC6-3xFLAG-EGFP #2	This study	shRNA target sequence: GCCTACTCCTTCGCAACATTC
pLKO.1-shControl-3xFLAG-EGFP #1	This study	Target sequence: CAACAAGATGAAGAGCACCAA
pLKO.1-shControl-3xFLAG-EGFP #2	This study	Target sequence: CCTAAGGTTAAGTCGCCCTCG
pLKO.1-PPP4C-Puro	Sigma-Aldrich (MISSION shRNA Collection)	TRC IDs of the constructs. shPPP4C 1: TRCN0000002761. shPPP4C 2: TRCN0000002763. shPPP4C 3: TRCN0000010737
pLKO.1-Scrambled	A gift from Anthony Leung (from Addgene)	Addgene#136035
pLVX-TetOne-Flag-Ubiquitin-EGFP	This study	
PLenti6.3-PPP4R1-HA-Blast	This study	
PLenti6.3-PPP4R2-HA-Blast		
PLenti6.3-PPP4C-HA-Blast	This study	
PLenti6.3-PPP4C-V5-Blast	This study	
pLVX-TetOne-CCDC6-His- TurboRFP	This study	
pLVX-TetOne-FBXO42-Myc- TurboRFP	This study	
pLVX-TetOne-CCDC6-Flag-Puro	This study	
pLVX-TetOne-CCDC6(“All A” mutant)-Flag-Puro	This study	
pLVX-TetOne-CCDC6 (“1 A” mutant) -Flag-Puro	This study	
pLVX-TetOne-CCDC6-F423A-Flag-Puro	This study	
**Antibodies**		
FBXO42 mouse monoclonal antibody, clone OTI2G7 (formerly 2G7)	OriGene Technologies	Cat#: TA800210
FBXO42 polyclonal ABclonal antibody	ABclonal	Cat#: A14898
CCDC6 mouse monoclonal antibody (Q-23)	Santa Cruz Biotechnology	Cat#: sc-100309
CCDC6 rabbit polyclonal antibody	Proteintech	Cat#: 13717-1-AP
CCDC6 antibody	Sigma-Aldrich	Cat#: HPA019051-100UL
PPP4C mouse monoclonal antibody, clone OTI2H7	OriGene Technologies	Cat#: TA810219
PPP4C rabbit polyclonal antibody	Proteintech	Cat#: 10262-1-AP
Vinculin mouse monoclonal antibody, clone hVIN-1	Sigma-Aldrich	Cat#: V9131
Dykddddk Tag (D6W5B) rabbit monoclonal antibody	Cell Signaling Technology	Cat#: 14793
V5-Tag (D3H8Q) rabbit monoclonal antibody	Cell Signaling Technology	Cat#: 13202
Cul1 rabbit polyclonal antibody	Cell Signaling Technology	Cat#: 4995
HA-Tag rabbit monoclonal antibody [C29F4]	Cell Signaling Technology	Cat#: 3724
HA-Tag (6E2) mouse monoclonal antibody	Cell Signaling Technology	Cat#: 2367
PPP4R1 rabbit polyclonal antibody	Bethyl Laboratories	Cat#: A300-836A
K48 linkage Specific Polyubiquitin (D9D5) Rabbit monoclonal antibody	Cell Signaling Technology	Cat#: 8081S
PPP4R2 rabbit polyclonal antibody	Bethyl Laboratories	Cat#: A300-838A
PPP4R3 Alpha rabbit polyclonal antibody	Bethyl Laboratories	Cat#: A300-839A-T
PPP4R3 Beta rabbit polyclonal antibody	Bethyl Laboratories	Cat#: A300-842A-T
β-Actin mouse monoclonal antibody	Sigma-Aldrich	Cat#: A5441
**Oligonucleotides and other sequence-based reagents**		
TrueGuide sgRNA for CCDC6. DNA target sequence: GCTCTCCAGAAAATTGATGC.	Thermo Fisher Scientific	Cat#: A35534
sgRNA sequences (sequence of DNA targeted) for FBXO42: AGCATGGGGCCCCAGAACTG.	Thermo Fisher Scientific	Cat#: A35534
DNA primers used for Sanger sequencing and ICE Analysis. For FBXO42, PCR Forward: GGCCCCTGTAAGTTTTCCCA, PCR Reverse: CCATGTGCTAAGCCAGTGGTA. Sanger Sequencing: CTTTCAAGACCAGACAGGAGAG.	Integrated DNA Technologies	
DNA Primers used for Sanger sequencing and ICE Analysis. CCDC6: PCR Forward: AGCAAAGAATAAAGAGGTGCCC, PCR Reverse: ACTAGTACACCGAACTCCCC. Sanger Sequencing: GGCCACATGTTTGCAGTATTC	Integrated DNA Technologies	
**Chemicals, enzymes and other reagents**		
Truecut™ Cas9 Protein v2	Thermo Fisher Scientific	Cat#: A36498
QuickExtract DNA Extraction Solution	Epicentre	Cat#: QE0905T
BI 2536	MedChem Express	Cat#: HY-50698
Rotenone	ApexBio	Cat#: B5462
PrestoBlue™ Cell Viability Reagent	Thermo Fisher Scientific	Cat#: A13262
Protease Inhibitor tablet (Complete, Mini, EDTA-Free)	Sigma-Aldrich	Cat#: 4693159001
Phosstop™- phosphatase inhibitor tablets	Roche	Cat#: 04906837001
4–20% polyacrylamide Bio-Rad Criterion TGX Midi gels	Bio-Rad	Cat#: 5671095
4–15% polyacrylamide Bio-Rad Criterion TGX Midi gels	Bio-Rad	Cat#: 5671085
Pierce™ ECL Western Blotting Substrate	Thermo Fisher Scientific	Cat#: 32209
Supersignal West Femto Maximum Sensitivity Chemiluminescent Substrate	Thermo Fisher Scientific	Cat#: 34096
Fetal Bovine Serum, Value, Heat-Inactivated	Thermo Fisher Scientific	Cat#: A5256801
MG-132	Selleck Chemicals	Cat#: S2619
Doxycycline hyclate	Sigma-Aldrich	Cat#: D9891-1G
Pevonedistat (MLN4924)	MedChem Express	Cat#: HY-70062
N-Ethylmaleimide (NEM)	Selleck Chemicals	Cat#: S3692
Palbociclib (hydrochloride)	MedChem Express	Cat#: HY-50767C
Colchicine	Selleck Chemicals	Cat#: S2284
RNAse A from bovine pancreas	Sigma-Aldrich	Cat#: 10109169001
Propidium iodide	Sigma-Aldrich	Cat#: P4170
FuGENE HD Transfection Reagent	Fugent	Cat#: HD-1000
Tris/Glycine/SDS Electrophoresis Buffer, 10X	Bio-Rad	Cat#: 1610772
DMEM/F-12	Thermo Fisher Scientific	Cat#: 11-320-082
IMDM	Thermo Fisher Scientific	Cat#: 12440061
DMEM	Thermo Fisher Scientific	Cat#: 11960069
Gibco Antibiotic-Antimycotic (100X)	Thermo Fisher Scientific	Cat#: 15240062
Glutamax Supplement	Thermo Fisher Scientific	Cat#: 35050061
Cytiva’s Amersham Protran 0.2 NC	Cytiva	Cat#: 10600001
Blotting Paper, 3MM Chr Celulose, 0.34 mm Thick	Cytiva	Cat#: 3030-392
**Software**		
GraphPad Prism		
Image Studio	LICORbio	
Alphafold 3 Server		
Uniprot		
DepMap		
**Other**		
Neon Electroporation System	Thermo Fisher Scientific	
Attune NxT Flow Cytometer	Thermo Fisher Scientific	
Vanquish Neo UHPLC System and Orbitrap Astral Mass Spectrometer	Thermo Fisher Scientific	

### Human cell culture

All cells were cultured in a humidified, 37 °C incubator, with 5% CO_2_ atmosphere. In total, 10% heat inactivated fetal bovine serum (FBS), 10 mM HEPES pH 7.5, and 1× Antibiotic-Antimycotic (Anti-Anti) was added to all media unless otherwise specified. RPE1 cells were cultured in DMEM/F12 media (Thermo Fisher Scientific 11-320-033). LN-18 cells and HEK 293T cells were cultured in DMEM, high glucose, no glutamine (Thermo Fisher Scientific 11960069), with 1X GlutaMAX added. HAP1 cells were cultured in IMDM without added HEPES. DPBS without calcium and magnesium were used to wash cells, Trypsin-EDTA (0.25%) was used to dissociate cells. Cells were maintained in aseptic conditions using standard culture methods, with subculturing every 3 or 4 days at split ratios ranging from 1:10 to 1:20 depending on the growth of the cells. Cells were tested for mycoplasma regularly.

### Establishment of clonal RPE1 FBXO42 and CCDC6 knockout lines

Knockouts were generated through electroporation-based transfection of Cas9-sgRNA ribonucleoprotein particles. The sgRNA sequences (sequence of DNA targeted) were: CCDC6: GCTCTCCAGAAAATTGATGC, FBXO42: AGCATGGGGCCCCAGAACTG. The Neon Electroporation system was assembled in a sterile biosafety cabinet according to manufacturer instructions. Work was all performed under sterile conditions, and the work area, pipettes, and gloves of researchers were cleaned with RNase-Zap. Purified sgRNA were ordered from Thermo Fisher Scientific. sgRNA was resuspended at 100 µM in sterile, RNase-free TE buffer (10 mM Tris-HCl pH 8.0, 0.1 mM EDTA). Purified TrueCut Cas9 Protein (Thermo Fisher), supplied was supplied at 5 µg/µl. For each reaction, 2 µg (0.4 µl) Cas9 was combined with 12 pmol sgRNA, brought to a total volume of 5 µl with Buffer R (Thermo Fisher). We have observed that PBS without Calcium and Magnesium is an acceptable substitute for Buffer R. This mixture for a single reaction, was scaled up to perform six reactions per sgRNA construct. This mixture was incubated for at least 10 min at room temperature to allow for complex formation.

A 12-well plate was prepared with antibiotic-free media, and placed in the 37 °C, 5% CO_2_ incubator to equilibrate. Cells were washed with DPBS, and trypsinized. Trypsin was quenched with media containing 10% FBS. Cells were counted using a hemocytometer or an automated cell counter. Cells were centrifuged (300 ×* g*, 5 min) and washed with DPBS. Cells were centrifuged again, and resuspended in Buffer R at a concentration of 25 million viable cells per ml. Cells were mixed at a 1:1 ratio to the pre-complexed Cas9-sgRNA, and mixed gently, taking care to avoid bubble formation. Electroporation was performed according to manufacturer instructions, with the electroporation conditions of 1300 V, 2 pulses, 20 ms pulse width. Electroporated cells were deposited in a 12-well plate well with pre-warmed, antibiotic-free media, one well per electroporation (approximately 125,000 cells/well).

Two days later, cells were passaged, seeded for single cell, and some were collected for analysis of knockout efficiency. Approximately 100,000 cells were collected, and DNA was extracted using QuickExtract according to manufacturer instructions. Extracted genomic DNA was PCR amplified and sent for Sanger sequencing. ICE analysis (Conant et al, 2022) was used to estimate the fraction of cells with InDels and knockout inducing mutations. This information was used to estimate how many clones should be screened for knockout mutations. A FACS instrument was used to deposit single cells into wells of a 96-well plate to isolate clones. Single cells grew for approximately two weeks until there were enough cells to analyze, and then clones were sequenced using Sanger sequencing or the region flanking the expected CRISPR cut sites. The following primers were used for Sanger sequencing and ICE Analysis. For FBXO42, PCR Forward: GGCCCCTGTAAGTTTTCCCA, PCR Reverse: CCATGTGCTAAGCCAGTGGTA, Sanger Sequencing: CTTTCAAGACCAGACAGGAGAG; CCDC6: PCR Forward: AGCAAAGAATAAAGAGGTGCCC, PCR Reverse: ACTAGTACACCGAACTCCCC, Sanger Sequencing: GGCCACATGTTTGCAGTATTC. Clones with predicted knockouts (e.g. mutations causing a frameshift, see Fig. EV1) were further validated using western blotting.

### Lentivirus production and transduction

All handling of lentivirus was performed with BSL-2+ safety procedures. Lentivirus packaging was performed with 2nd generation lentiviral packaging plasmids psPAX2 (a gift from Didier Trono (Addgene plasmid # 12260; http://n2t.net/addgene:12260; RRID:Addgene_12260)) and pMD2.G (a gift from Didier Trono (Addgene plasmid # 12259; http://n2t.net/addgene:12259; RRID:Addgene_12259)). For lentivirus production, 9.5 ×106 HEK 293 T cells were seeded in one T-75 flask per lentiviral construct, in 16 ml DMEM based culture media. The next day, media was aspirated and replaced with 8 ml lentivirus packaging media (Opti-MEM, 5% FBS, 200 μM sodium pyruvate, no antibiotics, no added HEPES). 800 μl Opti-MEM, 1940 ng pMD2.G, 2656 ng psPAX.2, and 3978 ng of the transfer plasmid containing the gene or shRNA sequence of interest were combined and incubated for 10 min at room temperature with periodic mixing. This mixture was added to the cells. Four hours later, media was changed to 16 ml fresh lentivirus packaging media. Virus containing media was collected 24 and 48 h after transfection and combined. Virus containing media was centrifuged at 150 × *g* for 7 min to pellet any loose HEK 293T cells. The virus-containing supernatant was then filtered with a 0.45-μm syringe filter. Transfections could be scaled up or down, depending on how much virus was required, based on the surface area of the cultureware used. Virus containing media was stored at 4 °C for up to about 2 months, or snap frozen and stored at −80 °C.

For transductions, cells were passaged the day before such that they were at about 20% confluence on the day of transduction. Cell media was replaced with fresh, complete culture media with 8 μg/ml polybrene. Virus containing media was then added at a dilution factor determined by empirical optimization, typically 1:8 or 1:16. Media containing virus was removed the following day and replaced with fresh culture media.

### Plasmids

GFP expressing shRNA Constructs were cloned into a plasmid backbone where EGFP is expressed from a hPGK promoter, and shRNA sequences are expressed from a U6 promoter. shRNA targeting sequences were: FBXO42 1: CAAACAGTGGTATCGACTTAT, FBXO42 2: TAGATGATGCAACTATCTTAA, CCDC6: GCCTACTCCTTCGCAACATTC, Scramble control: CCTAAGGTTAAGTCGCCCTCG.

Plasmids expressing shRNA against PPP4C were purchased from the Sigma-Aldrich MISSION shRNA Collection, and are in the pLKO.1 vector. The following are the TRC IDs of the constructs, followed by the targeted sequence: shPPP4C 1: TRCN0000002761, CGGCTACCTATTTGGCAGTGA; shPPP4C 2: TRCN0000002763, CATCAAGGAGAGCGAAGTCAA; shPPP4C 3: TRCN0000010737, GTGCTTCGAGGGTGAGGACTT. The scramble control, in the same vector, was acquired from Addgene, a gift from Anthony Leung (Addgene plasmid # 136035; http://n2t.net/addgene:136035; RRID:Addgene_136035).

For the plasmid expressing FLAG-tagged ubiquitin, the pLVX-TetOne backbone was used. Backbone was linearized with EcoRI and BamHI restriction enzymes. Insert contains a FLAG tag, the coding sequence for ribosomal protein RPS27A (which includes an N-terminal fusion with ubiquitin, that is cleaved off to form free ubiquitin post-translationally), followed by a “self cleaving” P2A sequence, then EGFP. Inserts were PCR amplified and the whole construct was assembled by Gibson assembly (Gibson et al, 2009). After this construct was transduced into RPE1 cells (see above), GFP expression was induced by doxycycline treatment (1 µg/ml, 24 h) and cells were sorted by FACS to select for GFP expressing cells. FACS selection was repeated three times to select for cells that consistently express the FLAG ubiquitin containing plasmid. Cell GFP expression (following dox induction) was periodically monitored to ensure the cells maintained consistent expression.

To overexpress epitope tagged versions of PP4 subunits, cDNA sequences for PPP4C, PPP4R1, and PPP4R2 in pLenti6.3 were purchased from DNAsu. The V5 tag was replaced by 4× HA tags using a gBlock, PCR amplification of the backbone, and Gibson assembly. Constructs were transduced into RPE1 cells with lentivirus (see above), and cells were selected with 5 μg/ml blasticidin.

To overexpress epitope-tagged versions of CCDC6 and FBXO42, the protein coding sequences were obtained as gBlocks (IDT). They were cloned into a pLVX-TetOne-Puro backbone using Gibson cloning. In both cases, a GGGS linker was added after the C-terminal of the gene before the epitope tag. A 6xHis tag (six histidines) was added to CCDC6, and a Myc tag (EQKLISEED) was added to FBXO42. Linkers and tags were added by PCR using the following primers:

FBXO42-Myc forward:ctgattagcgaagaagatctgTAAGGATCCAGACCACCTC, FBXO42-Myc Reverse: tttctgttcagagcctccaccTCTCTTTGCTCGTACAAAG, CCDC6-6xHis Forward: catcatcaccaccacTAAGGATCCAGACCACCTCCC, CCDC6-6xHis Reverse: atgagagcctccaccAGGCTGGGAGGAGGGGTG.

Constructs were transduced into RPE1 cells with lentivirus (see above), and cells were selected with 5 μg/ml puromycin. We made versions of the plasmids where the Puro selection marker was replaced by TurboRFP. TurboRFP was amplified from pCW57-RFP-2A-MCS (gift from Adam Karpf (Addgene plasmid # 78933; http://n2t.net/addgene:78933; RRID:Addgene_78933)) with these primers. Forward: GTTGATTGTTCCAGACGCGTATGAGCGAGCTGATCAAGGA, Reverse: GGCTTTTGCAAAACGCGACCTTATCTGTGCCCCAGTTTGC. The plasmid backbones containing FBXO42 or CCDC6 coding sequences were amplified with the following primers, which flank the area around the Puro gene. Forward: GCAAACTGGGGCACAGATAAGGTCGCGTTTTGCAAAAGCC, Reverse: TCCTTGATCAGCTCGCTCATACGCGTCTGGAACAATCAACC. PCR reactions were performed using SuperFI Platinum Taq II according to manufacturer instructions. Amplified backbones were digested with DpnI to remove parental plasmids, prior to Gibson assembly. Constructs were transduced into RPE1 cells with lentivirus (see above), and cells were selected with at least three rounds of sorting based on TurboRFP expression.

For constructs to express CCDC6 mutant variants, the pLVX-TetOne-Puro backbone plasmid expressing CCDC6 was used as a starting point. Geneart DNA Strings were ordered from Thermo Fisher that included the 3xFLAG tag and coding regions of CCDC6 with the desired mutations added. Sequences were codon optimized for expression in human cells using the Geneart web interface. The plasmid backbone and DNA strings were PCR amplified and assembled using Gibson assembly (Gibson et al, 2009). The mutations in construct “All A” are: Q102A, E110A, N114A, K118A, E139A, N143A, E203A, E211A, N215A, K219A, E326A, E334A, N338A. The mutants in “1A” are: N114A, N143A, N215A, and N338A. The F423A mutant had only that mutation. Codons were optomized for expression in human cells and for manufacturer using the Geneart software. Lentivirus was used to express these constructs in RPE1 cells with CCDC6 knocked out. Cells were selected with 5 µg/ml puromycin.

### Presto blue viability assay

LN-18 cells were seeded to 96-well plates at 3000 cells per well, with 200 µl culture media per well. For experiments with BI-2536 and rotenone, cells were seeded into 96-well plates in 100 μl media. The following day, compounds were added to wells at 2 times the desired final concentration, in 100 μl per well. For viability analysis, Presto Blue was used according to manufacturer instructions with the following modifications. Presto Blue concentrate was diluted 1 part to 10 parts complete, 37 °C culture media. Culture media was removed from the cells by firmly flicking the plate over a large beaker. Residual media was removed by tapping the plate, inverted, on a lint-free paper wipe (e.g., Kimwipe). In all, 100 µl presto blue in media was added to each well. The plate was incubated at 37 °C for 1 h. Plates were then analyzed with an Agilent BioTek Synergy Neo2 plate reader using fluorescent mode, excitation 560 nm, emission 590 nm. On each plate, wells with no cells were given media and other manipulations and used as blanks. The average blank signal was subtracted from all other well signals during analysis.

### Western blots

Cells were lysed by resuspending pellets in NP-40 based lysis buffer (100 mM Tris pH 8, 150 mM NaCl, 5 mM EDTA, 0.1% NP-40, complete Protease Inhibitor tablet, and PhoSTOP tablet 100 mM PMSF added right before use). Cells were incubated at 4 °C on a rotator for 30 min. Lysates were clarified by centrifuging at 20,000×*g* for 20 min, and supernatants were transferred to new tubes. Protein concentrations were measured using the bicinchoninic acid (BCA) assay (Smith et al, 1985). Up to 30 μg protein was loaded per SDS-PAGE gel lane. Western blotting was performed using standard methods (Burnette, 1981). Proteins were resolved using 4–15% or 4–20% polyacrylamide Bio-Rad Criterion TGX Midi gels, with tris-glycine-SDS running buffer, at 150 V for approximately 90 min. Gels were transferred to nitrocellulose membranes using a “wet transfer” method, with a Bio-Rad Criterion Blotter, and transfer buffer with 20 mM Tris, 150 mM glycine, 0.08% SDS, and 20% methanol, at 100 V for 60 min, at 4 °C. Ponceau stains were performed to verify correct transfer. Blocking was performed using blocking buffer according to the primary antibody manufacturer instructions, usually 5% (w/v) bovine serum albumin (BSA) or non-fat dry milk in PBS with 0.05% tween (PBST). Importantly, we found that when blotting with the FBXO42 antibody, 3% non-fat dry milk in PBS with Tween produced the best results. Primary antibodies were diluted according to manufacturer instructions in blocking buffer, typically at 1:1000. Sodium azide was added to the blocking buffer at 0.02% to prevent microbial growth. Blots were incubated with primary antibody overnight at 4 °C on an orbital shaker, overnight. Primary antibody dilutions could typically be reused multiple times. Following primary antibody incubation, blots were washed 3-4 times with PBST for 5 min each. HRP conjugated secondary antibodies were diluted 1:10,000 in the same blocking buffer as the primary antibody (without sodium azide). Blots were incubated with diluted secondary antibody for 1 h at room temperature, on an orbital shaker. For imaging, Pierce ECL Western Blotting Substrate was used as a substrate for chemiluminescence. For less abundant proteins in immunoprecipitation experiments, membranes were subsequently imaged again with SuperSignal West Femto Maximum Sensitivity Substrate (images marked as “high sensitivity” in figures). Imaging was performed using a LiCor XF.

### Immunoprecipitation

Cells were washed with DPBS and rapidly frozen on dry ice, as dry pellets. Pellets were lysed in lysis buffer (100 mM Tris pH 8, 150 mM NaCl, 5% Glycerol, 5 mM EDTA, 0.1% NP-40, complete Protease Inhibitor tablet, and PhoSTOP tablet) and passed through a 21 g needle ten times. Lysates were rotated at 4 °C for 30 min. Lysates were then cleared at 20,000 × *g* for 10 min at 4 °C. Supernatant was saved and a BCA assay was performed to measure protein concentration. At least 900 μg total protein lysate was used for each immunoprecipitation. Antibody was added at 9 μg per condition, in 300 μl lysis buffer, and lysates were then incubated at 4 °C for 2 h with rotation. Protein A Dynabeads were pre-washed three times in 1 ml of lysis buffer and then resuspended in the original volume with lysis buffer. In all, 300 µl samples were then incubated with 150 µl of Protein A Dynabeads for 1 h at 4 °C with rotation. After the bead incubation, flow through was saved before the beads were washed five times with 1 mL of lysis buffer. At each wash step, beads were mixed by pipetting. To elute, Elution Buffer (50 mM Tris pH 8.0, 0.5% SDS, 12.5 mM EDTA) was added to each sample and samples were incubated at 65 °C for 10 min. The elution was saved and frozen at −80 °C for downstream analysis.

The FBXO42 IP-MS was performed using HAP1 WT cells and HAP1 FBXO42^−/−^ clones, described in (Hundley et al, 2021), in duplicate. Cells were cultured in IMDM media supplemented with 10% FBS and scaled up to six 15-cm dishes. Cells were treated with 10 μM MG132 being added in the last 4 h of treatment. The CCDC6 IP-MS was performed the same way, except using RPE1 cells. For the shPPP4C condition, RPE1 cells were transduced with PPP4C shRNA 1 and 2 (see above) in equal parts, 3 days before harvest. For experiments with overexpressed FBXO42, CCDC6, and PPP4C, expression of FBXO42 and CCDC6 was induced by adding doxycycline to the cell culture media at 2 μg/ml, 2 days before cell collection.

For ubiquitin immunoprecipitation experiments, all cells were treated with 10 μM MG-132 for 4 h before collection. FLAG-ubiquitin expression was induced by addition of doxycycline to the cell culture media at 1–2 μg/ml 24 h before cell collection. MLN4924 was added to cells at 1 μM final concentration 24 h before harvest, where noted. For these experiments, 25 mM N-Ethylmaleamide (NEM) was added to the lysis buffer, as a DUB inhibitor. Samples were denatured to remove protein-protein interactions leaving covalent bonds (including ubiquitin-peptide bonds) using a protocol adapted from published methods (DeCaprio and Kohl, 2020). Cell pellets were resuspended in at least 200 µl denaturing lysis buffer (1% SDS, 50 mM Tris-HCl 50 mM). Samples were heated at 98 °C for 10 min. In total, 1800 µl Regular lysis buffer (described above) with protease inhibitors, phosphatase inhibitors, and NEM (in case any enzymatic activity survived) were then added. This is to dilute the SDS to reduce interference with antibody binding at later steps. Lysates were passed through a 21-gauge syringe 10 times to shear DNA. Lysates were centrifuged at 10,000×*g*, at 4 °C and transferred to a new tube. Lysates were incubated on a rotator at 4 °C for 10 min, then centrifuged again at 10,000×*g*, at 4 °C and transferred to a new tube. The IP was then continued normally. At least 4000 µg and as much as 7000 µg total protein lysate was used. Immunoprecipitation was performed with anti-HA, anti-FLAG, or anti-V5 beads according to manufacturer instructions. Washes were performed using high salt RIPA buffer (50 mM Tris-HCl pH 7.4, 750 mM NaCl, 1% Triton X-100, 0.5% sodium deoxycholate, 0.1% SDS, 1 mM EDTA). For FLAG-Ub IPs, elutions were performed in a by adding 81.25 µl elution buffer (50 mM Tris-HCl pH 8, 0.5% SDS) to beads and incubating beads at 65 °C for 10 min. Elution buffer was removed from beads, and 31.25 µl 4x loading buffer and 12.5 µl 1 M DTT was added. The samples were heated at 98 °C for 5 min before proceeding with western blotting. For V5 IPs, elution was performed by incubating the beads with 1 mg/ml V5 peptide in PBS, at 30 °C, on a rotator for 10 min.

### Mass-spectrometry proteomics analysis

For the experiment with HAP1 cells (Fig. 1A), eluates in 8 M urea and 100 mM Tris-Cl (pH 8.5), obtained after immunoprecipitation, were reduced with 5 mM tris (2-carboxyethyl)phosphine and alkylated with 10 mM iodoacetamide. The reduced and alkylated proteins were then cleaned using the single-pot, solid-phase-enhanced sample preparation (SP3) protocol for protein purification (Hughes et al, 2019). Following cleanup, proteins were digested overnight at 37 °C with the proteases Lys-C and trypsin. The resulting peptides were further purified using an offline SP3-based cleanup protocol, dried, and analyzed by LC-MS/MS. Details of the LC-MS/MS method are described in (Jami-Alahmadi et al, 2021).

Mass spectrometry data were processed using the MaxQuant bioinformatics pipeline (Cox and Mann, 2008), with peptide identification carried out by the Andromeda search engine against the *Homo sapiens* reference proteome (UniProt ID: UP000005640). Key settings included: a maximum of two missed cleavages, a false discovery rate (FDR) of 1% for both peptides and proteins, label-free quantification (LFQ) enabled with a minimum LFQ ratio count of 1, and parent and precursor ion tolerances of 20 and 4.5 ppm, respectively.

Processed MaxQuant output files were further analyzed for differential protein enrichment using the significance analysis of interactome (SAINT) pipeline (Choi et al, 2011).

For the experiments with RPE1 cells (Fig. 2A–C), the eluates obtained after immunoprecipitation or whole cell lysates were reduced and alkylated using 5 mM tris(2-carboxyethyl)phosphine and with 10 mM iodoacetamide, respectively. The reduced and alkylated proteins were purified using the protein aggregation capture (PAC) method (Batth et al, 2019), following which, proteins were digested overnight at 37 °C with Lys-C and trypsin proteases. The resulting peptides were dried and subjected to LC-MS/MS analysis.

Dried tryptic peptides, resuspended in 5% formic acid, were injected into a PepSep C18 reverse-phase column (150 mm × 150 µm, 1.7 µm particle size) maintained at 59 °C and electrosprayed into an Orbitrap Astral mass spectrometer. Chromatographic separation employing a trap and elute workflow was performed using a Vanquish Neo UHPLC system. Water with 0.1% formic acid comprised the mobile phase A, while acetonitrile with 0.1% formic acid comprised mobile phase B. A 15-min gradient was applied as follows: 5% B from 0–1 min (flow rate = 2.45 µL/min), 5%–15% B from 1–5 min (1.75 µL/min), 15%–25% B from 5–12.6 min (1.75 µL/min), 25%–38% B from 12.6–13.6 min (1.75 µL/min), and 38%–80% B from 13.6–13.7 min (2.45 µL/min), followed by holding at 80% B until 15 min (2.45 µL/min).

Data-independent acquisition (DIA) was performed using the Astral mass spectrometer operating in positive electrospray ionization mode. MS1 spectra were acquired at a resolution of 240,000 over an *m/z* range of 380–980, with a normalized AGC target of 500% and a maximum injection time of 3 ms. DIA was conducted using sequential 4 *m/z*-wide isolation windows across the 380–980 *m/z* range. MS2 spectra were collected at a resolution of 80,000, with a normalized higher-energy collisional dissociation (HCD) energy of 25%, a normalized AGC target of 500%, and a maximum injection time of 7 ms.

Thermo RAW files were searched using DIA-NN against an in silico library generated from the Homo sapiens reference proteome (UniProt ID: UP000005640) (Demichev et al, 2020). The resulting DIA-NN output was further analyzed with FragPipe Analyst to identify differentially regulated proteins (Hsiao et al, 2024).

The raw data files were analyzed using FragPipe to obtain protein identifications and their respective label-free quantification values. Of note, the data were normalized based on the assumption that the majority of proteins do not change between the different conditions. Data was first filtered for contaminant proteins. In addition, proteins that have been only identified by a single peptide and proteins not identified/quantified consistently in the same condition have been removed as well. The data was converted to log_2_ scale, samples were grouped by conditions and missing values were imputed using man method, which uses random draws from a left-shifted Gaussian distribution of 1.8 StDev (standard deviation) apart with a width of 0.3. Protein-wise linear models combined with empirical Bayes statistics were used for the differential expression analyses. The limma package from R Bioconductor was used to generate a list of differentially expressed proteins for each pair-wise comparison. A cutoff of the adjusted *P* value of 0.05 (params$fdr_correction method) along with a |log2 fold change| of 1 has been applied to determine differentially expressed proteins in each pairwise comparison.

### Flow cytometry-based competitive growth assay

For flow-cytometry, GFP-based, competitive growth assays, cells were transduced using high-titer lentivirus to express shRNA constructs with GFP markers. The next day, cells were dissociated and analyzed by flow cytometry to ensure high GFP expression. Cells were mixed at an approximately 1 to 1 ratio with untreated, GFP-negative cells. The mixed cells were then analyzed by flow cytometry to establish a time zero GFP expression fraction. Mixed GFP+ and GFP- cells were then seeded to 96-well plates at 3000 cells per well for LN-18 cells and 500 cells per well for RPE1 cells. At various time points, cells were analyzed by flow cytometry using a modified version of established protocol (Spangenberg et al, 2021). Media was removed from plates by flicking (in a biosafety cabinet to contain potentially hazardous aerosols). Cells were washed with DPBS (again removed by flicking). In all, 25 μl of 0.25% trypsin was added to each well, and plates were incubated at 37 °C until cells appeared loosened under the microscope. Then, 200 μl cold FACS Buffer (DPBS with 2% FBS, 1 mM EDTA, 10 mM HEPES and (optionally) 1× Antibiotic-Antimycotic) was added to each well. The cells in each well were mixed by repeated pipetting. Cells were kept at 4 °C or on ice from this point on, until analysis. An Attune NxT flow cytometer was used to analyze cells and establish the percent GFP + . For RPE1 cells, cells were passaged when they reached confluence by dissociating them as described and then transferring one-tenth of the volume of cells to new 96-well plates with fresh media.

For combined shRNA experiments, cells were transduced with GFP marked shRNA constructs (targeting FBXO42, CCDC6, or scramble controls) and puromycin resistance (Puro) marked shRNA constructs (targeting PPP4C or a scramble control) at the same time. The next day, media was removed, and fresh media with 2 μg/ml puromycin was added. After 3 days of puromycin selection (and 4 days post-transduction) cells were dissociated, analyzed, and mixed as described above.

For presto blue analysis, cells were treated and set up in the same way, except that instead of mixing GFP+ and GFP- cells, just 3000 GFP+ cells were seeded to each 96-well plate well.

### Cell cycle analysis

LN-18 cells were transduced with shRNA constructs as described above. Cells were FACS sorted on GFP+ and allowed to grow for 10 days. Additional cells were treated with palbociclib (1 µM) or colchicine (30 nM) to create controls for G1 or G2/M arrest, respectively. Cells were then trypsinized and FACS sorted again on GFP+ cells. FACS sorting was also used to count cells to ensure equal number of cells were used for propidium iodide staining for each condition. Cells were washed with DPBS and pellet by centrifugation. In total, 500 µl ice-cold ethanol was added dropwise to cell pellets, while vortexing to avoid clumps. Cells were incubated at −20 °C overnight. Cells were then centrifuged at 1500 × *g* for 5 min for all steps post-fixation. Cells were washed twice with DPBS. Propidium iodide (PI) solution was prepared, DPBS with 0.1% Triton X-100, 20 µg/ml, 200 µg/ml RNase A from bovine pancreas (heat-inactivated at 98 °C for 5 min to ablate any DNase activity). Cells were resuspended in PI staining solution and incubated at 37 °C for 30 min to allow for RNA digestion. Cells were analyzed using an Attune NxT flow cytometer. Data was analyzed using FlowJo, and cell cycle was modeled using the Dean-Jett-Fox model.

### Phosphoproteomics

Lysates containing 200 µg of protein were prepared using the Suspension Trapping (S-Trap) columns. Lysates were mixed 1:1 with 2× lysis buffer containing 10% SDS and 100 mM triethylammonium bicarbonate (TEAB), pH 8.5, followed by reduction with 5 mM TCEP and alkylation with 10 mM iodoacetamide. Samples were acidified with 12% phosphoric acid, then diluted with binding/wash buffer (100 mM TEAB, pH 7.55 in 90% methanol) at 6× the sample volume and mixed. The resulting suspension was loaded onto S-Trap columns and centrifuged at 4000×* g* for 30 s. Columns were washed three times with binding/wash buffer and centrifuged again at 4000 × *g* for 1 min.

Proteins were digested on-column overnight at 37 °C with trypsin and lysC at enzyme-to-protein ratios of 1:25 and 1:50, respectively, in 50 mM TEAB, pH 8.5. Peptides were sequentially eluted with 50 mM TEAB (pH 8.5), 0.2% formic acid, and 50% acetonitrile, each followed by centrifugation at 4000× *g* for 1 min. Eluates were pooled, dried, desalted, and dried again.

Dried peptides were resuspended in phosphopeptide binding buffer (80% acetonitrile, 5% TFA, 0.1 M glycolic acid) and enriched for phosphopeptides using Ti-IMAC magnetic beads (Resyn Bio) as described in (Bekker-Jensen et al, 2020). Briefly, the beads were equilibrated three times in binding buffer and added to peptides at a protein-to-beads ratio of 1:4. The mixture was incubated with mixing for 20 min, followed by magnetic separation of beads and supernatant. Beads were washed once with binding buffer, once with wash buffer 1 (80% acetonitrile, 1% TFA), and once with wash buffer 2 (10% acetonitrile, 0.2% TFA). Phosphopeptides were eluted with 1% ammonium hydroxide for 20 min and immediately acidified with 10% formic acid. Eluates were dried and analyzed by LC-MS/MS as described above.

Raw data generated from phosphopeptide enriched samples were processed with DIA-NN built-in workflow for detecting phosphorylation modification using an in-silico library generated using DIA-NN spectral library generation feature, from UniProt human proteome database with following specifications: 2 missed cleavages, 3 maximum number of variable modifications and precursor charge 2–5 (Demichev et al, 2020). The DIANN outputs were analyzed as described previously.

### Statistical analysis and normalization

GraphPad Prism was used for statistical analysis. Western blots were quantified using Image Studio. For normalization of ubiquitin IPs, the signal associated with the ubiquitin smear was normalized by dividing the ubiquitin signal by the V5 (PPP4C) input signal (for FLAG-ubiquitin IPs) or the V5 elution signal (PPP4C-V5 IPs) and the FLAG-ubiquitin elution (FLAG-ubiquitin) or input signals (PPP4C-V5 IPs). One-way ANOVA with repeated measures was used, with each replicate blot treated as one measure. Values displayed in graphs are normalized to the WT sample. ANOVA was performed on log_2_ transformed values and on values prior to normalization to the WT sample to avoid losing variability. All one-way ANOVAs used Dunnett’s multiple hypothesis test for pairwise comparisons. Blinding was used for proteomics experiments, but not for most other experiments.

### Alphafold predictions

Performed with Alphafold 3 Server. Amino acid sequences for CCDC6 (Q16204) and PPP4C (P60510) were retrieved from UniProt.

## Supplementary information

Peer Review File

Dataset EV1

Source data Fig. 1

Source data Fig. 2

Source data Fig. 3

Source data Fig. 4

Source data Fig. 5

Expanded View Figures

## Data Availability

Raw data for all proteomics experiments has been deposited at MassIVE. ID: MSV000100795. The source data of this paper are collected in the following database record: biostudies:S-SCDT-10_1038-S44319-026-00828-y.

## References

[CR1] Abramson J, Adler J, Dunger J, Evans R, Green T, Pritzel A, Ronneberger O, Willmore L, Ballard AJ, Bambrick J et al (2024) Accurate structure prediction of biomolecular interactions with AlphaFold 3. Nature 630:493–500 38718835 10.1038/s41586-024-07487-w PMC11168924

[CR2] Baek K, Krist DT, Prabu JR, Hill S, Klügel M, Neumaier L-M, von Gronau S, Kleiger G, Schulman BA (2020) NEDD8 nucleates a multivalent cullin–RING–UBE2D ubiquitin ligation assembly. Nature 578:461–466 32051583 10.1038/s41586-020-2000-y PMC7050210

[CR3] Bai C, Sen P, Hofmann K, Ma L, Goebl M, Harper JW, Elledge SJ (1996) SKP1 connects cell cycle regulators to the ubiquitin proteolysis machinery through a novel motif, the F-Box. Cell 86:263–274 8706131 10.1016/s0092-8674(00)80098-7

[CR4] Barbosa P, Zhaunova L, Debilio S, Steccanella V, Kelly V, Ly T, Ohkura H (2020) SCF-Fbxo42 promotes synaptonemal complex assembly by downregulating PP2A-B56. J Cell Biol 220:e202009167 10.1083/jcb.202009167 PMC7780726 33382409

[CR5] Batth TS, Tollenaere MX, Rüther P, Gonzalez-Franquesa A, Prabhakar BS, Bekker-Jensen S, Deshmukh AS, Olsen JV (2019) Protein aggregation capture on microparticles enables multipurpose proteomics sample preparation. Mol Cell Proteom 18:1027–1035 10.1074/mcp.TIR118.001270 PMC6495262 30833379

[CR6] Bekker-Jensen DB, Bernhardt OM, Hogrebe A, Martinez-Val A, Verbeke L, Gandhi T, Kelstrup CD, Reiter L, Olsen JV (2020) Rapid and site-specific deep phosphoproteome profiling by data-independent acquisition without the need for spectral libraries. Nat Commun 11:787 32034161 10.1038/s41467-020-14609-1 PMC7005859

[CR7] Bekker-Jensen DB, Kelstrup CD, Batth TS, Larsen SC, Haldrup C, Bramsen JB, Sørensen KD, Høyer S, Ørntoft TF, Andersen CL et al (2017) An optimized shotgun strategy for the rapid generation of comprehensive human proteomes. Cell Syst 4:587–599.e584 28601559 10.1016/j.cels.2017.05.009 PMC5493283

[CR8] Bennett EJ, Rush J, Gygi SP, Harper JW (2010) Dynamics of cullin-RING ubiquitin ligase network revealed by systematic quantitative proteomics. Cell 143:951–965 21145461 10.1016/j.cell.2010.11.017 PMC3008586

[CR9] Brownell JE, Sintchak MD, Gavin JM, Liao H, Bruzzese FJ, Bump NJ, Soucy TA, Milhollen MA, Yang X, Burkhardt AL et al (2010) Substrate-assisted inhibition of ubiquitin-like protein-activating enzymes: the NEDD8 E1 inhibitor MLN4924 forms a NEDD8-AMP mimetic in situ. Mol Cell 37:102–111 20129059 10.1016/j.molcel.2009.12.024

[CR10] Burnette WN (1981) “Western blotting”: electrophoretic transfer of proteins from sodium dodecyl sulfate-polyacrylamide gels to unmodified nitrocellulose and radiographic detection with antibody and radioiodinated protein A. Anal Biochem 112:195–203 6266278 10.1016/0003-2697(81)90281-5

[CR11] Celetti A, Cerrato A, Merolla F, Vitagliano D, Vecchio G, Grieco M (2004) H4(D10S170), a gene frequently rearranged with RET in papillary thyroid carcinomas: functional characterization. Oncogene 23:109–121 14712216 10.1038/sj.onc.1206981

[CR12] Cerrato A, Merolla F, Morra F, Celetti A (2018) CCDC6: the identity of a protein known to be partner in fusion. Int J Cancer 142:1300–1308 29044514 10.1002/ijc.31106

[CR13] Chen MJ, Dixon JE, Manning G (2017) Genomics and evolution of protein phosphatases. Sci Signal 10:eaag1796 28400531 10.1126/scisignal.aag1796

[CR14] Choi H, Larsen B, Lin Z-Y, Breitkreutz A, Mellacheruvu D, Fermin D, Qin ZS, Tyers M, Gingras A-C, Nesvizhskii AI (2011) SAINT: probabilistic scoring of affinity purification–mass spectrometry data. Nat Methods 8:70–73 21131968 10.1038/nmeth.1541 PMC3064265

[CR15] Conant D, Hsiau T, Rossi N, Oki J, Maures T, Waite K, Yang J, Joshi S, Kelso R, Holden K et al (2022) Inference of CRISPR edits from Sanger trace data. CRISPR J 5:123–130 35119294 10.1089/crispr.2021.0113

[CR16] Consortium, T.U. (2024) UniProt: the Universal Protein Knowledgebase in 2025. Nucleic Acids Res 53:D609–D617 10.1093/nar/gkae1010 PMC11701636 39552041

[CR17] Cox J, Mann M (2008) MaxQuant enables high peptide identification rates, individualized p.p.b.-range mass accuracies and proteome-wide protein quantification. Nat Biotechnol 26:1367–1372 19029910 10.1038/nbt.1511

[CR18] Craig KL, Tyers M (1999) The F-box: a new motif for ubiquitin dependent proteolysis in cell cycle regulation and signal transduction. Prog Biophys Mol Biol 72:299–328 10581972 10.1016/s0079-6107(99)00010-3

[CR19] DeCaprio J, Kohl TO (2020) Denaturing lysis of cells for immunoprecipitation. Cold Spring Harb Protoc 2020:098616 32015005 10.1101/pdb.prot098616

[CR20] Demichev V, Messner CB, Vernardis SI, Lilley KS, Ralser M (2020) DIA-NN: neural networks and interference correction enable deep proteome coverage in high throughput. Nat Methods 17:41–44 31768060 10.1038/s41592-019-0638-x PMC6949130

[CR21] Deshaies RJ, Joazeiro CAP (2009) RING domain E3 ubiquitin ligases. Annu Rev Biochem 78:399–434 19489725 10.1146/annurev.biochem.78.101807.093809

[CR22] Fischer ES, Böhm K, Lydeard JR, Yang H, Stadler MB, Cavadini S, Nagel J, Serluca F, Acker V, Lingaraju GM et al (2014) Structure of the DDB1–CRBN E3 ubiquitin ligase in complex with thalidomide. Nature 512:49–53 25043012 10.1038/nature13527 PMC4423819

[CR23] Frescas D, Pagano M (2008) Deregulated proteolysis by the F-box proteins SKP2 and beta-TrCP: tipping the scales of cancer. Nat Rev Cancer 8:438–449 18500245 10.1038/nrc2396 PMC2711846

[CR24] Gibson DG, Young L, Chuang R-Y, Venter JC, Hutchison CA, Smith HO (2009) Enzymatic assembly of DNA molecules up to several hundred kilobases. Nat Methods 6:343–345 19363495 10.1038/nmeth.1318

[CR25] Grim JE, Gustafson MP, Hirata RK, Hagar AC, Swanger J, Welcker M, Hwang HC, Ericsson J, Russell DW, Clurman BE (2008) Isoform- and cell cycle-dependent substrate degradation by the Fbw7 ubiquitin ligase. J Cell Biol 181:913–920 18559665 10.1083/jcb.200802076 PMC2426948

[CR26] Hoellerbauer P, Kufeld M, Arora S, Mitchell K, Girard EJ, Herman JA, Olson JM, Paddison PJ (2024) FBXO42 activity is required to prevent mitotic arrest, spindle assembly checkpoint activation and lethality in glioblastoma and other cancers. NAR Cancer 6:zcae021 38774470 10.1093/narcan/zcae021 PMC11106029

[CR27] Hsiao Y, Zhang H, Li GX, Deng Y, Yu F, Valipour Kahrood H, Steele JR, Schittenhelm RB, Nesvizhskii AI (2024) Analysis and visualization of quantitative proteomics data using FragPipe-Analyst. J Proteome Res 23:4303–4315 39254081 10.1021/acs.jproteome.4c00294 PMC13142904

[CR28] Huang X, Liu J, Shen T, Meng X, Dou L, Lin Y, Li J (2016) Protein phosphatase 4 plays dual roles during cell proliferation. Cell Prolif 49:219–235 27041735 10.1111/cpr.12249 PMC6496217

[CR29] Hughes CS, Moggridge S, Müller T, Sorensen PH, Morin GB, Krijgsveld J (2019) Single-pot, solid-phase-enhanced sample preparation for proteomics experiments. Nat Protoc 14:68–85 30464214 10.1038/s41596-018-0082-x

[CR30] Hundley FV, Sanvisens Delgado N, Marin HC, Carr KL, Tian R, Toczyski DP (2021) A comprehensive phenotypic CRISPR-Cas9 screen of the ubiquitin pathway uncovers roles of ubiquitin ligases in mitosis. Mol Cell 81:1319–1336.e1319 33539788 10.1016/j.molcel.2021.01.014

[CR31] Huttlin EL, Bruckner RJ, Navarrete-Perea J, Cannon JR, Baltier K, Gebreab F, Gygi MP, Thornock A, Zarraga G, Tam S et al (2021) Dual proteome-scale networks reveal cell-specific remodeling of the human interactome. Cell 184:3022–3040.e3028 33961781 10.1016/j.cell.2021.04.011 PMC8165030

[CR32] Huttlin EL, Bruckner RJ, Paulo JA, Cannon JR, Ting L, Baltier K, Colby G, Gebreab F, Gygi MP, Parzen H et al (2017) Architecture of the human interactome defines protein communities and disease networks. Nature 545:505–509 28514442 10.1038/nature22366 PMC5531611

[CR33] Jami-Alahmadi Y, Pandey V, Mayank AK, Wohlschlegel JA (2021) A robust method for packing high resolution C18 RP-nano-HPLC columns. J Vis Exp 10:10–3791 10.3791/62380 34057454

[CR34] Jiang H, Bian W, Sui Y, Li H, Zhao H, Wang W, Li X (2022) FBXO42 facilitates Notch signaling activation and global chromatin relaxation by promoting K63-linked polyubiquitination of RBPJ. Sci Adv 8:eabq4831 36129980 10.1126/sciadv.abq4831 PMC9491713

[CR35] Jin J, Cardozo T, Lovering RC, Elledge SJ, Pagano M, Harper JW (2004) Systematic analysis and nomenclature of mammalian F-box proteins. Genes Dev 18:2573–2580 15520277 10.1101/gad.1255304 PMC525538

[CR36] Jumper J, Evans R, Pritzel A, Green T, Figurnov M, Ronneberger O, Tunyasuvunakool K, Bates R, Žídek A, Potapenko A et al (2021) Highly accurate protein structure prediction with AlphaFold. Nature 596:583–589 34265844 10.1038/s41586-021-03819-2 PMC8371605

[CR37] Kipreos ET, Pagano M (2000) The F-box protein family. Genome Biol 1:Reviews3002 11178263 10.1186/gb-2000-1-5-reviews3002 PMC138887

[CR38] Li AY, McCusker MG, Russo A, Scilla KA, Gittens A, Arensmeyer K, Mehra R, Adamo V, Rolfo C (2019) RET fusions in solid tumors. Cancer Treat Rev 81:101911 31715421 10.1016/j.ctrv.2019.101911

[CR39] Liwocha J, Li J, Purser N, Rattanasopa C, Maiwald S, Krist DT, Scott DC, Steigenberger B, Prabu JR, Schulman BA et al (2024) Mechanism of millisecond Lys48-linked poly-ubiquitin chain formation by cullin-RING ligases. Nat Struct Mol Biol 31:378–389 38326650 10.1038/s41594-023-01206-1 PMC10873206

[CR40] Lü Y, Cho T, Mukherjee S, Suarez CF, Gonzalez-Foutel NS, Malik A, Martinez S, Dervovic D, Oh RH, Langille E et al (2024) Genome-wide CRISPR screens identify novel regulators of wild-type and mutant p53 stability. Mol Syst Biol 20:719–740 38580884 10.1038/s44320-024-00032-x PMC11148184

[CR41] Martin-Granados C, Philp A, Oxenham SK, Prescott AR, Cohen PTW (2008) Depletion of protein phosphatase 4 in human cells reveals essential roles in centrosome maturation, cell migration and the regulation of Rho GTPases. Int J Biochem Cell Biol 40:2315–2332 18487071 10.1016/j.biocel.2008.03.021

[CR42] Mathieson T, Franken H, Kosinski J, Kurzawa N, Zinn N, Sweetman G, Poeckel D, Ratnu VS, Schramm M, Becher I et al (2018) Systematic analysis of protein turnover in primary cells. Nat Commun 9:689 29449567 10.1038/s41467-018-03106-1 PMC5814408

[CR43] Merolla F, Luise C, Muller MT, Pacelli R, Fusco A, Celetti A (2012) Loss of CCDC6, the first identified RET partner gene, affects pH2AX S139 levels and accelerates mitotic entry upon DNA damage. PLoS ONE 7:e36177 22655027 10.1371/journal.pone.0036177 PMC3360053

[CR44] Merolla F, Pentimalli F, Pacelli R, Vecchio G, Fusco A, Grieco M, Celetti A (2007) Involvement of H4(D10S170) protein in ATM-dependent response to DNA damage. Oncogene 26:6167–6175 17420723 10.1038/sj.onc.1210446

[CR45] Morra F, Merolla F, Damia G, Ricci F, Varricchio S, Ilardi G, Arenare L, Califano D, Napolitano V, Fruscio R et al (2022) The disruption of the CCDC6 – PP4 axis induces a BRCAness like phenotype and sensitivity to PARP inhibitors in high-grade serous ovarian carcinoma. J Exp Clin Cancer Res 41:245 35964058 10.1186/s13046-022-02459-2 PMC9375931

[CR46] Nagler A, Vredevoogd DW, Alon M, Cheng PF, Trabish S, Kalaora S, Arafeh R, Goldin V, Levesque MP, Peeper DS et al (2020) A genome-wide CRISPR screen identifies FBXO42 involvement in resistance toward MEK inhibition in NRAS-mutant melanoma. Pigment Cell Melanoma Res 33:334–344 31549767 10.1111/pcmr.12825 PMC7383499

[CR47] Oughtred R, Rust J, Chang C, Breitkreutz BJ, Stark C, Willems A, Boucher L, Leung G, Kolas N, Zhang F et al (2021) The BioGRID database: a comprehensive biomedical resource of curated protein, genetic, and chemical interactions. Protein Sci 30:187–200 33070389 10.1002/pro.3978 PMC7737760

[CR48] Park J, Lee DH (2020) Functional roles of protein phosphatase 4 in multiple aspects of cellular physiology: a friend and a foe. BMB Rep 53:181–190 32192570 10.5483/BMBRep.2020.53.4.019 PMC7196183

[CR49] Rape M, Reddy SK, Kirschner MW (2006) The processivity of multiubiquitination by the APC determines the order of substrate degradation. Cell 124:89–103 16413484 10.1016/j.cell.2005.10.032

[CR50] Santos CC, Schweizer N, Cairrão F, Ramirez J, Osinalde N, Yang M, Gaspar CJ, Rasheva VI, Trigo ML, Hensel Z et al (2025) Fbxo42 promotes the degradation of Ataxin-2 granules to trigger terminal Xbp1 signaling. Nat Commun 16:7523 40804044 10.1038/s41467-025-62417-2 PMC12350939

[CR51] Senft D, Qi J, Ronai ZEA (2018) Ubiquitin ligases in oncogenic transformation and cancer therapy. Nat Rev Cancer 18:69–88 29242641 10.1038/nrc.2017.105 PMC6054770

[CR52] Shaltiel IA, Aprelia M, Saurin AT, Chowdhury D, Kops GJPL, Voest EE, Medema RH (2014) Distinct phosphatases antagonize the p53 response in different phases of the cell cycle. Proc Natl Acad Sci USA 111:7313–7318 24711418 10.1073/pnas.1322021111 PMC4034242

[CR53] Skaar JR, D’Angiolella V, Pagan JK, Pagano M (2009) SnapShot: F box proteins II. Cell 137:1358.1358.e1351 19563764 10.1016/j.cell.2009.05.040

[CR54] Skowyra D, Craig KL, Tyers M, Elledge SJ, Harper JW (1997) F-Box proteins are receptors that recruit phosphorylated substrates to the SCF ubiquitin–ligase complex. Cell 91:209–219 9346238 10.1016/s0092-8674(00)80403-1

[CR55] Smith PK, Krohn RI, Hermanson GT, Mallia AK, Gartner FH, Provenzano MD, Fujimoto EK, Goeke NM, Olson BJ, Klenk DC (1985) Measurement of protein using bicinchoninic acid. Anal Biochem 150:76–85 3843705 10.1016/0003-2697(85)90442-7

[CR56] Soucy TA, Smith PG, Milhollen MA, Berger AJ, Gavin JM, Adhikari S, Brownell JE, Burke KE, Cardin DP, Critchley S et al (2009) An inhibitor of NEDD8-activating enzyme as a new approach to treat cancer. Nature 458:732–736 19360080 10.1038/nature07884

[CR57] Spangenberg SH, Zavareh RB, Lairson LL (2021) Protocol for high-throughput compound screening using flow cytometry in THP-1 cells. STAR Protoc 2:100400 33778785 10.1016/j.xpro.2021.100400 PMC7985391

[CR58] Thul PJ, Åkesson L, Wiking M, Mahdessian D, Geladaki A, Ait Blal H, Alm T, Asplund A, Björk L, Breckels LM et al (2017) A subcellular map of the human proteome. Science 356:eaal3321 28495876 10.1126/science.aal3321

[CR59] Toledo CM, Ding Y, Hoellerbauer P, Davis RJ, Basom R, Girard EJ, Lee E, Corrin P, Hart T, Bolouri H et al (2015) Genome-wide CRISPR-Cas9 screens reveal loss of redundancy between PKMYT1 and WEE1 in glioblastoma stem-like cells. Cell Rep 13:2425–2439 26673326 10.1016/j.celrep.2015.11.021 PMC4691575

[CR60] Toyo-oka K, Mori D, Yano Y, Shiota M, Iwao H, Goto H, Inagaki M, Hiraiwa N, Muramatsu M, Wynshaw-Boris A et al (2008) Protein phosphatase 4 catalytic subunit regulates Cdk1 activity and microtubule organization via NDEL1 dephosphorylation. J Cell Biol 180:1133–1147 18347064 10.1083/jcb.200705148 PMC2290842

[CR61] Tsherniak A, Vazquez F, Montgomery PG, Weir BA, Kryukov G, Cowley GS, Gill S, Harrington WF, Pantel S, Krill-Burger JM et al (2017) Defining a cancer dependency map. Cell 170:564–576.e516 28753430 10.1016/j.cell.2017.06.010 PMC5667678

[CR62] Tyers M, Willems AR (1999) One ring to rule a superfamily of E3 ubiquitin ligases. Science 284:601–604 10328744 10.1126/science.284.5414.601

[CR63] Ueki Y, Kruse T, Weisser MB, Sundell GN, Larsen MSY, Mendez BL, Jenkins NP, Garvanska DH, Cressey L, Zhang G et al (2019) A consensus binding motif for the PP4 protein phosphatase. Mol Cell 76:953–964.e956 31585692 10.1016/j.molcel.2019.08.029 PMC6981294

[CR64] Uhlén M, Fagerberg L, Hallström BM, Lindskog C, Oksvold P, Mardinoglu A, Sivertsson Å, Kampf C, Sjöstedt E, Asplund A et al (2015) Proteomics. Tissue-based map of the human proteome. Science 347:1260419 25613900 10.1126/science.1260419

[CR65] Voss M, Campbell K, Saranzewa N, Campbell DG, Hastie CJ, Peggie MW, Martin-Granados C, Prescott AR, Cohen PT (2013) Protein phosphatase 4 is phosphorylated and inactivated by Cdk in response to spindle toxins and interacts with γ-tubulin. Cell Cycle 12:2876–2887 23966160 10.4161/cc.25919 PMC3899200

[CR66] Wu T, Jiang X, Xu B, Zhong Q, Zheng J, Zhang X, Wang Y (2022) High expression of CCDC6 in relation to unfavorable outcome and immune cells infiltration in hepatobiliary carcinoma. J Cancer 13:3378–3395 36186907 10.7150/jca.76050 PMC9516016

[CR67] Yang H, Smith P, Ma Y, Southworth E, Gopala Krishna V, Salerno B, Rowland J, Loftus AEP, Grieco D, Vendrell I et al (2026) Pervasive phenotypic effects of FBXO42 are promoted by regulation of PP4 phosphatase. EMBO J 45:1332 10.1038/s44318-025-00675-y PMC12909836 41484364

[CR68] Zheng N, Schulman BA, Song L, Miller JJ, Jeffrey PD, Wang P, Chu C, Koepp DM, Elledge SJ, Pagano M et al (2002) Structure of the Cul1–Rbx1–Skp1–F boxSkp2 SCF ubiquitin ligase complex. Nature 416:703–709 11961546 10.1038/416703a

[CR69] Zhou J, Li Q, Deng X, Peng L, Sun J, Zhang Y, Du Y (2024) Comprehensive analysis identifies ubiquitin ligase FBXO42 as a tumor-promoting factor in neuroblastoma. Sci Rep 14:18697 39134694 10.1038/s41598-024-69760-2 PMC11319589

