# Supplementary material for: The regulation of protein phosphatase 4 by FBXO42 is required for cancer cell survival
Source: EMBO Rep. 2026 Jul 14;27(15):4211–37. doi: 10.1038/s44319-026-00828-y (PMC13458699; doi:10.1038/s44319-026-00828-y)
Supplement: Supplementary file 1 [file 44319_2026_828_MOESM1_ESM.pdf]

EMBO reports

The regulation of Protein Phosphatase 4 by FBXO42 is required for cancer cell survival.

Stephan Spangenberg, Nicholas Garaffo, Enya Alcindor, Ben Lusk, Vijaya Pandey, Adam Longhurst, Emma Bolech, Becky Xu Hua Fu, Luke Gilbert, James Wohlschlegel, and David Toczyski

Corresponding author(s): David Toczyski (David.Toczyski@ucsf.edu)

Review Timeline:

Transfer Date:	4th Nov 25
Editorial Decision:	15th Dec 25
Revision Received:	23rd Feb 26
Editorial Decision:	16th Apr 26
Revision Received:	7th May 26
Accepted:	28th May 26

Editor: Martina Rembold

Transaction Report: This manuscript was transferred to EMBO Reports following peer review at The EMBO Journal.

(Note: With the exception of the correction of typographical or spelling errors that could be a source of ambiguity, letters and reports are not edited. Depending on transfer agreements, referee reports obtained elsewhere may or may not be included in this compilation. Referee reports are anonymous unless the Referee chooses to sign their reports.)

Referee #1:

This work builds on previous work from the lab that identified FBXO42 as a possible regulator of cell division. FBXO42 is an adaptor protein for the SCF ubiquitin ligase that is poorly characterised but importantly is required for cell fitness in 15% of cancers. In DepMap there are clear genetic links between FBXO42 and p53 and recent work has suggested that FBXO42 might regulate stability of certain p53 mutants. FBXO42 also shows strong genetic links with CCDC6 in DepMap and these proteins appear to make a complex that regulate p53 in an unknown manner. From the literature and prior work there is thus clearly unexplored and relevant biological pathways to explore.
To characterise these further the authors first investigate genes that are contributing stronger to cellular fitness in the absence of FBXO42 by conducting a genome wide CRISPR KO screen in HAP1 cells. This uncovers that knockout of components of the gamma-tubulin complex is synthetic lethal with FBXO42 loss while knockout of the centriole regulator TUBE1 becomes less essential in FBXO42 null cells. As it has been reported that TUBE1 loss normally trigger p53 dependent cell death this makes the authors speculate that FBXO42 might be required for p53 activation upon centrosome defects. To investigate this they generate FBXO42 and CCDC6 KO RPE1 cell lines and look at p53 activation following addition of Plk4i and observe reduced p53 activation in both cell lines. To get insight into how FBXO42 and CCDC6 might activate p53 they conduct interactome studies of FBXO42 and identify components of the PP4 phosphatase complex (PP4C, PPP4R1, PPP4R2) and provide evidence that FBXO42 might ubiquitinate PP4C to control the activity of this phosphatase. They further show that CCDC6 and FBXO42 bind independently to PP4 but that PP4 activity is required for CCDC6-FBXO42 complex formation. Finally, they explore if the negative regulation of PP4 by FBXO42 and CCDC6 is responsible for their regulation of cell growth using a glioblastoma cell line for this in which depletion of FBXO42 and CCDC6 caused a decrease in cell growth. Indeed, co-depletion of PP4C seems to rescue cell growth consistent with this idea.

My overall impression of this work is that the experiments are well conducted, and interesting biology is uncovered but the work is somewhat premature and lacks mechanistic insight and experiments tying the different observations together. It seems at present that the four figures more or less represent independent experiments with limited connections. In its present state I do not feel that EMBO J is an appropriate fit but if more work is conducted it might be.

I have below highlighted some of the major issues I find with this work which maybe can help the authors:

1) There is limited connection between the role of FBXO42 in activating p53 in response to centrosome defects and the regulation of FBXO42 in regulating PP4. If the authors could show that p53 activation by FBXO42 is dependent on its negative regulation of PP4 this would make connections between the first part of the paper and the second part. Without this connection it could be two separate things they are looking at. This could be done similar to the experiments in 2E-F but depleting PP4C to see if this rescues p53 activation. This should be complemented with better characterisation of the RPE1 KO cell lines and mitotic/centrosome defects - is for instance the mitotic phenotype in FBXO42 rescued by PP4 depletion (this relates to figure 4 where they show that this is the case for growth).
2) Validation of the hits from HAP1 screen in figure 1 in the RPE1 cells used in figure 2. As they shift cell lines it would be important to show this to couple these two figures. This should be complemented with a more careful characterisation of centrosomes by microscopy in the RPE1 cell lines generated preferentially in p53 positive and negative RPE1 cells.
3) The effects on PP4C ubiquitination and protein levels seems subtle and would need quantification to be convincing. In figure 3D why are PP4C levels reduced in FBXO42 -/-? Would be good to check if PP4C is indeed more active in FBXO42 -/- and CCDC6 -/- cells by doing in vitro phosphatase assays on the PP4C IPs.
4) AF models are premature and not supported by experiments. It is pretty clear that AF will try and make models and without any validation this should not go into a paper.

Referee #2:

In this manuscript, the authors investigate genetic factors that influence sensitivity to FBXO42 depletion. They perform genome-wide CRISPR-Cas9 screens in the presence and absence of mitotic inhibitors-specifically, the PLK1 inhibitor BI-2536 and the microtubule poison colchicine. Their screens identify the mitotic genes MZT1 and MZT2B, which are essential for microtubule nucleation at the centrosome, as synthetic lethal with FBXO42 deletion. In contrast, loss of TUBE1, which is involved in centriole formation, appears to rescue the FBXO42-deficient phenotype.
The authors further examine the roles of FBXO42 and CCDC6 in p53 activation following centrosome depletion, proposing that both are required to trigger a p53 response in the absence of centrosomes. They then identify protein phosphatase 4 (PP4) components as FBXO42 interactors and show that one subunit (PPP4C) is ubiquitinated in an FBXO42-

dependent manner. They report that CCDC6 also interacts with PP4 but is dispensable for PPP4C ubiquitination. Finally, they show that depletion of PPP4C rescues the sensitivity of LN-18 glioblastoma cells to FBXO42 deletion.

This manuscript presents a series of observations regarding genetic interactions involving FBXO42, centrosomal components, and the p53 pathway. While the study includes genome-wide CRISPR screens and follow-up biochemical analyses, the work remains largely descriptive and lacks the mechanistic depth required to support the central claims. Key findings are not sufficiently validated, many conclusions are speculative or inferred without direct evidence, and several critical experiments lack appropriate controls or quantification

Overall, the manuscript does not meet the standards for publication in its current form. Significant additional experimentation is required to establish clear mechanistic insight, ensure reproducibility, and validate the biological relevance of the reported interactions. The authors are encouraged to rigorously address the concerns detailed below to strengthen the scientific foundation of their study.

Major Comments:

1. Phenotypic Characterization of FBXO42 KO

a) The authors must show the phenotype of FBXO42 knockout in their model cell line (HAP1) under the same conditions used in the screen.

b) The cellular consequences of colchicine and BI-2536 treatment in both wild-type and FBXO42 KO cells should be clearly demonstrated-ideally through live-cell imaging to observe mitotic progression, arrest, or defects.

2. Follow-up on Genetic Interactions:

a) The cell biological phenotypes of MZT1/MZT2B and TUBE1 knockout, both alone and in combination with FBXO42 knockout, should be analyzed. Live-cell imaging should reveal whether synthetic lethality correlates with exacerbated mitotic defects.

b) The authors suggest that TUBE1 knockout rescues the FBXO42 phenotype via effects on p53. If so, they must show that TUBE1 KO induces prolonged mitosis and p53 activation, and that this is suppressed in the FBXO42/TUBE1 double knockout.

3. Interpretation of Screen Results:

a) The authors should explain why other known centrosomal or centriolar genes were not identified in the screen.

b) They must also reconcile why TUBE1 was found specifically in the PLK1 inhibitor screen, given that PLK1 is required for p53 activation in response to prolonged mitosis.

4. Inconsistencies with Published Literature:
The claim that FBXO42 and CCDC6 are essential for p53 activation following centrosome loss contrasts with findings from prior genome-wide screens (e.g., Fong et al., 2016; Lambrus et al., 2016; Meitinger et al., 2016), which identified canonical factors like USP28, 53BP1, p53, and p21, but not FBXO42 or CCDC6. The authors must address this discrepancy. To confirm that FBXO42 and CCDC6 are in the same pathway as USP28 and 53BP1, the authors could perform long term proliferation assays in the presence of Centrinone B. RPE1 cells usually arrest after 4 days, while USP28 or 53BP1 knockout allows cells to proliferate for at least 12 days.

5. Use of PLK4 Inhibitor Centrinone B:
a) The conditions used for centrosome depletion via PLK4 inhibition are not clearly validated. The authors should confirm, via live-cell imaging, that their Centrinone B treatment leads to gradual centrosome loss, prolonged mitosis, and p53 activation. Without this, the biological context of their experiments remains ambiguous.

b) Immunoblotting for p21 and p53 must be repeated under well-controlled conditions, quantified, and replicated (minimum of three biological replicates), as current data are unconvincing.

6. Unclear Biochemical Experiments:
a) The presentation of co-immunoprecipitation experiments (e.g., Figure 3B) is insufficient. Tag information, antibody specificity, and lane identities must be clearly indicated.

b) The functional relevance of the interaction between FBXO42, CCDC6, and PP4 components remains unclear. How this ties into p53 activation or centrosome integrity is not addressed.

7. Unexplained Rescue by PPP4C Depletion:
a) The rescue of FBXO42 deletion by PPP4C knockdown in LN-18 cells is intriguing but unexplored. There is no characterization of the FBXO42 KO phenotype in LN-18 cells, nor any mechanistic insight into why PPP4C loss compensates for it.

b) The fact that PPP4C was not identified in the initial CRISPR screens also raises concerns. Are the authors observing distinct mechanisms in different cell lines?

8. Speculative Alphafold Predictions:
The manuscript includes a paragraph discussing AlphaFold3-predicted interactions without any experimental validation. Relying solely on predictive models is not appropriate in this context and weakens the credibility of the mechanistic claims. The predicted interaction sites should be tested experimentally.

Referee #3:

In the manuscript entitled "Regulation of Protein Phosphatase 4 by FBXO42 Is Essential for Cancer Cell Survival," the authors investigated how mitosis and cancer cell survival are regulated by the FBXO42-CCDC6-PPP4C complex. In a genome-wide CRISPR screen, they identified FBXO42 as a critical regulator of centrosome formation, and data from DepMap further support that FBXO42 is functionally linked to CCDC6. Mass spectrometry analysis showed a physical interaction between FBXO42 and PPP4C. The authors claimed that PPP4C is a ubiquitin substrate of the SCF(FBXO42) E3 ligase complex. Notably, depletion of FBXO42 or CCDC6 suppresses glioblastoma cell viability, whereas PPP4C depletion enhances it.

While the study attempts to clarify the underlying mechanisms suggested by previous literature, additional mechanistic insights, biological relevance, and clinical implications are necessary to confirm the findings and support the conclusions.

Major comments:

Figure 2: It would be beneficial to provide cell cycle profiling data using FBXO42- and CCDC6-depleted cells to demonstrate potential mitotic defects.

Figure 3 - Ubiquitination assay: The assay conducted using a standard IP with the target protein and blotting for ubiquitin may not accurately evaluate the ubiquitination status. The authors should also perform the assay by pulling down ubiquitinated proteins under denaturing conditions, followed by blotting for PPP4C.

Figure 3 - Ubiquitination assay: The authors should evaluate whether FBXO42-mediated PPP4C ubiquitination involves specific ubiquitin linkages by using ubiquitin expression constructs mutated at various lysine residues (e.g., K48, K63).

Figure 3 - Ubiquitination assay: An in vitro ubiquitination assay would be beneficial to directly demonstrate the modification of PPP4C by FBXO42.

The authors need to demonstrate changes in PPP4C protein half-life in cells with FBXO42 overexpression or depletion using cycloheximide chase assays or pulse-chase labeling.

Figures S3B, S3C, and S3D: It would be beneficial to test whether mutating the predicted PPP4C binding residues (Gln102, Glu110, Asn114, Lys118, Glu203, Glu211, Asn215, and Lys219) abolishes the interaction.

The specific role of CCDC6 in the complex remains unclear. Does CCDC6 function as an intrinsic inhibitor of PPP4C activity, or does it act as a scaffold protein that facilitates PPP4C ubiquitination? What are the underlying mechanisms behind the high co-dependency correlation between FBXO42 and CCDC6 observed in DepMap? The authors should provide experimental evidence or a mechanistic rationale explaining the significance of this complex in efficiently inhibiting PPP4C activity and function.

Figure S4B: Given the importance of the data, this panel should be moved to the main Figure 4 for emphasis and clarity.

Cell viability data using cultured cells is insufficient to demonstrate biological significance. An in vivo model, such as a xenograft assay, is required to validate the oncogenic effects of dysregulated FBXO42-CCDC6-PPP4C signaling using the genetically manipulated cell lines, such as those presented in Figure S4B.

It would be beneficial to show the clinical relevance of the FBXO42-CCDC6-PPP4C signaling complex in cancer. Is there any correlation in mRNA or protein levels among these components in patient clinical samples? Publicly available datasets could be utilized for correlation analysis across patient cohorts.

We would like to thank the reviewers for their constructive feedback. In response to this feedback and in consultation with the editors, we have made several revisions which we feel makes for a more-focused, complete manuscript, and better compliments the co-submitted manuscript by the D'Angiolella laboratory. We agree with the point raised by multiple reviewers and the editors that there was a lack of cohesion between the parts of the manuscript dealing with centrosome function and p53, and the parts evaluating the links between FBXO42, CCDC6, and PP4. To address this issue, we have removed the CRISPR screen showing genetic interactions with FBXO42 and the data related to p53 activation following centrosome depletion. While we feel that the data within these figures was important, significant additional work is needed to properly characterize the potential effects of FBXO42 on centrosome integrity and p53 signaling. The revised manuscript has been refocused and now provides a more in-depth investigation of the independent regulation of PP4 by FBXO42 and CCDC6. The reviewers also pointed out that Alphafold predictions of PPP4C-CCDC6 included in the previous version were unvalidated. We have subsequently generated mutant constructs and tested them is cells, and confirmed that binding sites on CCDC6 predicted by Alphafold are required for PPP4C-CCDC6 binding. In consideration of the manuscript's more narrow focus, we present it as a short report.

Referee #1:

This work builds on previous work from the lab that identified FBXO42 as a possible regulator of cell division. FBXO42 is an adaptor protein for the SCF ubiquitin ligase that is poorly characterised but importantly is required for cell fitness in 15% of cancers. In DepMap there are clear genetic links between FBXO42 and p53 and recent work has suggested that FBXO42 might regulate stability of certain p53 mutants. FBXO42 also shows strong genetic links with CCDC6 in DepMap and these proteins appear to make a complex that regulate p53 in an unknown manner. From the literature and prior work there is thus clearly unexplored and relevant biological pathways to explore.
To characterise these further the authors first investigate genes that are contributing stronger to cellular fitness in the absence of FBXO42 by conducting a genome wide CRISPR KO screen in HAP1 cells. This uncovers that knockout of components of the gamma-tubulin complex is synthetic lethal with FBXO42 loss while knockout of the centriole regulator TUBE1 becomes less essential in FBXO42 null cells. As it has been reported that TUBE1 loss normally trigger p53 dependent cell death this makes the authors speculate that FBXO42 might be required for p53 activation upon centrosome defects. To investigate this they generate FBXO42 and CCDC6 KO RPE1 cell lines and look at p53 activation following addition of Plk4i and observe reduced p53 activation in both cell lines. To get

insight into how FBXO42 and CCDC6 might activate p53 they conduct interactome studies of FBXO42 and identify components of the PP4 phosphatase complex (PP4C, PPP4R1, PPP4R2) and provide evidence that FBXO42 might ubiquitinate PP4C to control the activity of this phosphatase. They further show that CCDC6 and FBXO42 bind independently to PP4 but that PP4 activity is required for CCDC6-FBXO42 complex formation. Finally, they explore if the negative regulation of PP4 by FBXO42 and CCDC6 is responsible for their regulation of cell growth using a glioblastoma cell line for this in which depletion of FBXO42 and CCDC6 caused a decrease in cell growth. Indeed, co-depletion of PP4C seems to rescue cell growth consistent with this idea.

My overall impression of this work is that the experiments are well conducted, and interesting biology is uncovered but the work is somewhat premature and lacks mechanistic insight and experiments tying the different observations together. It seems at present that the four figures more or less represent independent experiments with limited connections. In its present state I do not feel that EMBO J is an appropriate fit but if more work is conducted it might be.

I have below highlighted some of the major issues I find with this work which maybe can help the authors:

1) There is limited connection between the role of FBXO42 in activating p53 in response to centrosome defects and the regulation of FBXO42 in regulating PP4. If the authors could show that p53 activation by FBXO42 is dependent on its negative regulation of PP4 this would make connections between the first part of the paper and the second part. Without this connection it could be two separate things they are looking at. This could be done similar to the experiments in 2E-F but depleting PP4C to see if this rescues p53 activation. This should be complemented with better characterisation of the RPE1 KO cell lines and mitotic/centrosome defects - is for instance the mitotic phenotype in FBXO42 rescued by PP4 depletion (this relates to figure 4 where they show that this is the case for growth).

We thank the reviewer for this insightful comment. We agree that significant further work is needed to elucidate the connections between FBXO42, p53, and PP4. As such, we have narrowed the scope of the manuscript to focus on the relationship between FBXO42 and CCDC6 and PP4 and present these data in a short report format. We have removed the data and discussion of p53 regulation. The exact details of the impact of FBXO42 and PP4 on p53 is left as a direction for future studies.

2) Validation of the hits from HAP1 screen in figure 1 in the RPE1 cells used in figure 2. As they shift cell lines it would be important to show this to couple these two figures. This should be complemented with a more careful characterisation of centrosomes by microscopy in the RPE1 cell lines generated preferentially in p53 positive and negative RPE1 cells.

We agree that characterization of the effect of the genes from the screen on centrosome dynamics deserves further study. On a similar note to point 1, we feel that full characterization of these effects will require more time and study than we can accomplish in this revision. As such, we have re-focused the scope of this manuscript and excluded discussion of this screen and follow-up from it. Also, we have removed discussions of possible regulation of centrosome assembly by FBXO42. Instead, we have focused exclusively on FBXO42 and CCDC6's effects on PP4 regulation (Figure 3A, 4C, 4D 5B, 5C and new Figures 2E, 2F, 3B-F, 5D, S2B-C).

3) The effects on PP4C ubiquitination and protein levels seems subtle and would need quantification to be convincing. In figure 3D why are PP4C levels reduced in FBXO42 -/-? Would be good to check if PP4C is indeed more active in FBXO42 -/- and CCDC6 -/- cells by doing in vitro phosphatase assays on the PP4C IPs.

We thank the reviewer for this feedback. We have performed additional experiments to characterize ubiquitination of PPP4C. In particular, we have performed Ubiquitin IPs, blotting for V5-tagged PPP4C (new Figure 3B). We have now included quantification of replicates from this experiment (new Figure 3C). We also performed additional replicates immunoprecipitating PPP4C and blotting for ubiquitin (new Figures 3D, S2B). We thank the reviewer for pointing out the decreased levels of PPP4C in FBXO42$^{-/-}$ cells. We now examined effects of FBXO42 and CCDC6 depletion in both RPE1 (new Figure 3E,3F) and LN-18 cells (Figure 4C, 4D, 5A), both acutely using shRNA knockdown and in knockouts. Across these different contexts, we find that endogenously expressed PPP4C levels are consistently increased, by about 1.5-fold, in FBXO42 depleted/mutated cells (This is now quantified in new Figures 3F and 4D). However, exogenously overexpressed PPP4C levels vary and are frequently decreased in FBXO42$^{-/-}$ cells. This may be because FBXO42$^{-/-}$ cells are sensitive to high levels of PP4 and therefore cells with high expression are selectively lost from the population. In Figure 2D and 3B in the PPP4C blots, the bands for endogenously expressed and overexpressed PPP4C are both visible, and can be compared. While we were not able to perform in vitro phosphatase assays on IP'd PPP4C, we now provide data showing that phosphorylation levels of a strongly correlating set of proteins is

affected in both CCDC6 and FBXO42. We also provided data indicating that PPP4C is ubiquitinated with K48-linked ubiquitin, typically a degradation signal, and that ubiquitination is increased by proteosome inhibition (new Figures S2C and S2D).

4) AF models are premature and not supported by experiments. It is pretty clear that AF will try and make models and without any validation this should not go into a paper.

We thank the reviewer for raising this important point. We have performed additional experiments in cells, validating that the residues of CCDC6 highlighted in AlphaFold predictions are required for the CCDC6-PPP4C interaction (new Figure 5D).

Referee #2:

In this manuscript, the authors investigate genetic factors that influence sensitivity to FBXO42 depletion. They perform genome-wide CRISPR-Cas9 screens in the presence and absence of mitotic inhibitors-specifically, the PLK1 inhibitor BI-2536 and the microtubule poison colchicine. Their screens identify the mitotic genes MZT1 and MZT2B, which are essential for microtubule nucleation at the centrosome, as synthetic lethal with FBXO42 deletion. In contrast, loss of TUBE1, which is involved in centriole formation, appears to rescue the FBXO42-deficient phenotype.
The authors further examine the roles of FBXO42 and CCDC6 in p53 activation following centrosome depletion, proposing that both are required to trigger a p53 response in the absence of centrosomes. They then identify protein phosphatase 4 (PP4) components as FBXO42 interactors and show that one subunit (PPP4C) is ubiquitinated in an FBXO42-dependent manner. They report that CCDC6 also interacts with PP4 but is dispensable for PPP4C ubiquitination. Finally, they show that depletion of PPP4C rescues the sensitivity of LN-18 glioblastoma cells to FBXO42 deletion.

This manuscript presents a series of observations regarding genetic interactions involving FBXO42, centrosomal components, and the p53 pathway. While the study includes genome-wide CRISPR screens and follow-up biochemical analyses, the work remains largely descriptive and lacks the mechanistic depth required to support the central claims. Key findings are not sufficiently validated, many conclusions are speculative or inferred without direct evidence, and several critical experiments lack appropriate controls or quantification

Overall, the manuscript does not meet the standards for publication in its current form. Significant additional experimentation is required to establish clear mechanistic insight, ensure reproducibility, and validate the biological relevance of the reported interactions. The authors are encouraged to rigorously address the concerns detailed below to strengthen the scientific foundation of their study.

Major Comments:

1. Phenotypic Characterization of FBXO42 KO

a) The authors must show the phenotype of FBXO42 knockout in their model cell line (HAP1) under the same conditions used in the screen.

b) The cellular consequences of colchicine and BI-2536 treatment in both wild-type and FBXO42 KO cells should be clearly demonstrated-ideally through live-cell imaging to observe mitotic progression, arrest, or defects.

See below

2. Follow-up on Genetic Interactions:

a) The cell biological phenotypes of MZT1/MZT2B and TUBE1 knockout, both alone and in combination with FBXO42 knockout, should be analyzed. Live-cell imaging should reveal whether synthetic lethality correlates with exacerbated mitotic defects.

b) The authors suggest that TUBE1 knockout rescues the FBXO42 phenotype via effects on p53. If so, they must show that TUBE1 KO induces prolonged mitosis and p53 activation, and that this is suppressed in the FBXO42/TUBE1 double knockout.

See below

3. Interpretation of Screen Results:

a) The authors should explain why other known centrosomal or centriolar genes were not identified in the screen.

b) They must also reconcile why TUBE1 was found specifically in the PLK1 inhibitor screen, given that PLK1 is required for p53 activation in response to prolonged mitosis.

Regarding points 1-3. Both the reviewers and the editors felt that the manuscript lacked focus. We agree that significant further investigation is needed to elucidate the exact dynamics of FBXO42 regulation of p53, mitosis, and centrosome integrity. As such, we have now reduced the manuscript to a short report and narrowed the scope to focus on the parallel targeting of PP4 by FBXO42 and CCDC6.

4. Inconsistencies with Published Literature:
The claim that FBXO42 and CCDC6 are essential for p53 activation following centrosome loss contrasts with findings from prior genome-wide screens (e.g., Fong et al., 2016; Lambrus et al., 2016; Meitinger et al., 2016), which identified canonical factors like USP28, 53BP1, p53, and p21, but not FBXO42 or CCDC6. The authors must address this discrepancy. To confirm that FBXO42 and CCDC6 are in the same pathway as USP28 and 53BP1, the authors could perform long term proliferation assays in the presence of Centrinone B. RPE1 cells usually arrest after 4 days, while USP28 or 53BP1 knockout allows cells to proliferate for at least 12 days.

We thank the reviewer for addressing the relationship of our results with previous literature. We have decided to remove discussion of FBXO42 regulation of p53 and centrosomes until this pathway can be more thoroughly characterized. However, we should note that Fong et. Al., 2016 did find FBXO42 in their screen as the 7th strongest hit, and identified it in three independent screens (their Table 1) after p53, p53BP1, TRIM37, USP2, p21 and CHD8. Similarly, Tkach et. al., 2022, showed that FBXO42 depleted cells were more resistant to arrest following centrosome depletion with centrinone B alongside TP53, TP53BP1, USP28, and CDKN1A.

5. Use of PLK4 Inhibitor Centrinone B:
a) The conditions used for centrosome depletion via PLK4 inhibition are not clearly validated. The authors should confirm, via live-cell imaging, that their Centrinone B treatment leads to gradual centrosome loss, prolonged mitosis, and p53 activation. Without this, the biological context of their experiments remains ambiguous.

b) Immunoblotting for p21 and p53 must be repeated under well-controlled conditions, quantified, and replicated (minimum of three biological replicates), as current data are unconvincing.

We thank the reviewer for highlighting this important issue. As mentioned above, we have decided to remove all discussion of PLK4 inhibition from the manuscript. These questions will be addressed in future work.

6. Unclear Biochemical Experiments:
a) The presentation of co-immunoprecipitation experiments (e.g., Figure 3B) is insufficient. Tag information, antibody specificity, and lane identities must be clearly indicated.

We thank the reviewer for this comment. Additional labels have been added to figures and the relevant information has been added to figure legends, particularly for Figures 1E and 2D.

b) The functional relevance of the interaction between FBXO42, CCDC6, and PP4 components remains unclear. How this ties into p53 activation or centrosome integrity is not addressed.

We thank the reviewer for this comment. As mentioned above we have decided to exclude discussion of the possible role of FBXO42 and CCDC6 in p53 regulation. We have now included additional data on the interaction of FBXO42, CCDC6, and PPP4C. We have found that FBXO42 and CCDC6 have non-redundant roles in cell growth or survival through PP4 (Figure 2E, 2F). We have shown that depletion of FBXO42, but not CCDC6, reduces ubiquitination of PPP4C and found that this ubiquitination is largely K48 linked (new Figures 3B, 3C, 3D, S2B. S2C). We have also now identified a motif on CCDC6 involved in PPP4C interaction (new Figure 5D) and found that CCDC6 appears to interact specifically with the PPP4C-PPP4R1 heterodimer (Figure 2A, new Figure 5D). How FBXO42, CCDC6, and PP4 regulate p53 and centrosome integrity will be addressed in future work.

7. Unexplained Rescue by PPP4C Depletion:
a) The rescue of FBXO42 deletion by PPP4C knockdown in LN-18 cells is intriguing but unexplored. There is no characterization of the FBXO42 KO phenotype in LN-18 cells, nor any mechanistic insight into why PPP4C loss compensates for it.

We thank the reviewer for this comment. We have now shown that depletion of FBXO42 or CCDC6 cause LN-18 cells to increasingly be in phase G2/M, which is consistent with stalled or delayed mitosis (new Figure S2E). However, the exact substrate(s) of PPP4C responsible for this phenotype will be explored in future work.

b) The fact that PPP4C was not identified in the initial CRISPR screens also raises concerns. Are the authors observing distinct mechanisms in different cell lines?

We thank the reviewer for raising this important point. It could be that PPP4C's status as an essential gene prevented it from being identified in the CRISPR screen, which used CRISPR cleavage to generate null alleles. This is in contrast with the shRNA based approach we used in Figures 5A-C which reduced but did not completely abolish PPP4C expression. However, as mentioned above, we have removed the CRISPR screen until more extensive follow-up can be performed.

8. Speculative Alphafold Predictions:
The manuscript includes a paragraph discussing AlphaFold3-predicted interactions without any experimental validation. Relying solely on predictive models is not appropriate in this context and weakens the credibility of the mechanistic claims. The predicted interaction sites should be tested experimentally.

We thank the reviewer for raising this important point. We have performed additional experiment validating, in cells, that the residues of CCDC6 highlighted in Alphafold predictions are required for CCDC6-PPP4C interaction (new Figure 5D).

Referee #3:

In the manuscript entitled "Regulation of Protein Phosphatase 4 by FBXO42 Is Essential for Cancer Cell Survival," the authors investigated how mitosis and cancer cell survival are regulated by the FBXO42-CCDC6-PPP4C complex. In a genome-wide CRISPR screen, they identified FBXO42 as a critical regulator of centrosome formation, and data from DepMap further support that FBXO42 is functionally linked to CCDC6. Mass spectrometry analysis showed a physical interaction between FBXO42 and PPP4C. The authors claimed that PPP4C is a ubiquitin substrate of the SCF(FBXO42) E3 ligase complex. Notably, depletion of FBXO42 or CCDC6 suppresses glioblastoma cell viability, whereas PPP4C depletion enhances it.

While the study attempts to clarify the underlying mechanisms suggested by previous literature, additional mechanistic insights, biological relevance, and clinical implications are necessary to confirm the findings and support the conclusions.

Major comments:

Figure 2: It would be beneficial to provide cell cycle profiling data using FBXO42- and CCDC6-depleted cells to demonstrate potential mitotic defects.

We thank the reviewer for this recommendation. We have now performed cell cycle profiling of FBXO42 and CCDC6 depleted LN-18 cells. We find that depletion of FBXO42 or CCDC6 cause an increase in G2/M phase cells, consistent with mitotic defects (new Figure S2E).

Figure 3 - Ubiquitination assay: The assay conducted using a standard IP with the target protein and blotting for ubiquitin may not accurately evaluate the ubiquitination status. The authors should also perform the assay by pulling down ubiquitinated proteins under denaturing conditions, followed by blotting for PPP4C.

We thank the reviewer for this helpful suggestion. We have performed additional pull downs of both ubiquitinated proteins and of PPP4C under denaturing conditions (new Figures 3B, 3D). We have also provided quantification of replicate experiments (new Figures 3C, S2B).

Figure 3 - Ubiquitination assay: The authors should evaluate whether FBXO42-mediated PPP4C ubiquitination involves specific ubiquitin linkages by using ubiquitin expression constructs mutated at various lysine residues (e.g., K48, K63).

We thank the reviewer for this comment. We investigated K48 linkages in ubiquitinated PPP4C using antibodies specific for that linkage. We found that PPP4C appears to be ubiquitinated with K48 linked ubiquitin, consistent with enrichment of the ubiquitin smear on PPPC by MG132 (new Figures S2C, S2D, 3D). However, the additional presence of other ubiquitin linkages cannot be ruled out.

Figure 3 - Ubiquitination assay: An in vitro ubiquitination assay would be beneficial to directly demonstrate the modification of PPP4C by FBXO42.

We thank the reviewer for this recommendation. While we were not able to perform in vitro ubiquitination assays within a reasonable time frame for revisions, we have now shown that: endogenous PPP4C levels increase in FBXO42 mutants (new Figures 3E, 3F), that PPP4C is ubiquitinated with K48 linked chains in a FBXO42-dependent way (new Figure S2D), that this does not represent non-specific association as it can be seen in denaturing pulldowns (new Figures 3B, 3D), that loss of PPP4C rescues reduced proliferation in FBXO42 depleted cell (old Figures 5B, 5C), that phosphorylation levels are altered similarly

in FBXO42 mutants and CCDC6 mutants (new Figure S3A), and that FBXO42 binds PPP4C (old Figures 1A, 1E).

The authors need to demonstrate changes in PPP4C protein half-life in cells with FBXO42 overexpression or depletion using cycloheximide chase assays or pulse-chase labeling.

PPP4C has been reported to have a long half-life. As such we were unable to evaluate the half-life of PPP4C using typical cycloheximide chase experiments because the time with cycloheximide required to achieve significant depletion of bulk PPP4 levels exceeded the time cells could survive with translation inhibited. However, we have quantified increased PPP4C expression in FBXO42 depleted cells across multiple replicates and in different contexts (RPE1 cells and LN-18 cells, depletion by genetic knockout and by shRNA) in new Figures 3E, 3F and old figures 4C, and 4D. We also show that ubiquitinated PPP4C accumulates upon proteasome inhibition (new Figure S2C). We hypothesize that only a specific subset of PPP4C is degraded by FBXO42, making bulk half-life assays less informative. Characterizing exactly which fraction of cellular PPP4C is ubiquitinated by FBXO42, and its exact effects on PPP4C will be addressed in future work.

Figures S3B, S3C, and S3D: It would be beneficial to test whether mutating the predicted PPP4C binding residues (Gln102, Glu110, Asn114, Lys118, Glu203, Glu211, Asn215, and Lys219) abolishes the interaction.

We thank the reviewer for this comment. We have performed experiments in cells demonstrating that mutation of these residues abolishes PPP4C-CCDC6 binding (new Figure 5D).

The specific role of CCDC6 in the complex remains unclear. Does CCDC6 function as an intrinsic inhibitor of PPP4C activity, or does it act as a scaffold protein that facilitates PPP4C ubiquitination? What are the underlying mechanisms behind the high co-dependency correlation between FBXO42 and CCDC6 observed in DepMap? The authors should provide experimental evidence or a mechanistic rationale explaining the significance of this complex in efficiently inhibiting PPP4C activity and function.

We thank the reviewer for this comment. We now provide additional data to address these questions. Our current model is that FBXO42 and CCDC6 both bind PPP4C, but that they do so independently of each other (Figure 1E, 2A-D). FBXO42 ubiquitinates PPP4C (new Figures 3B, 3D) leading to its degradation (indicated by K48 ubiquitination (new Figure S2D) and accumulation of ubiquitinated PPP4C with proteasome inhibition (new Figure S2C))

and decreased protein levels (old Figures 4C, 4D, 5A, new Figures 3E, 3F). Conversely, CCDC6 is not required for ubiquitination of PPP4C (new Figures 3B, 3D). Considering that knockdown of PPP4C functionally compensates for depletion of CCDC6 (Figure 5B, 5C), we hypothesize that CCDC6's direct binding to PPP4C inhibits its activity. We identified a specific motif present four times in CCDC6 that is required for PPP4C binding. This model is described in our updated Results and Discussion section. We attempted to use peptides with the CCDC6 binding site for PPP4C in binding experiments, however these peptides appeared too small to form the proper structure for PPP4C binding. Therefore, the exact mechanism of how CCDC6 inhibits PPP4C activity will need to be further characterized in later work.

Figure S4B: Given the importance of the data, this panel should be moved to the main Figure 4 for emphasis and clarity.

We thank the reviewer for this comment, and we have made this change, data is now figure 5C.

Cell viability data using cultured cells is insufficient to demonstrate biological significance. An in vivo model, such as a xenograft assay, is required to validate the oncogenic effects of dysregulated FBXO42-CCDC6-PPP4C signaling using the genetically manipulated cell lines, such as those presented in Figure S4B.

It would be beneficial to show the clinical relevance of the FBXO42-CCDC6-PPP4C signaling complex in cancer. Is there any correlation in mRNA or protein levels among these components in patient clinical samples? Publicly available datasets could be utilized for correlation analysis across patient cohorts.

We thank the reviewer for this comment. We feel that additional, future work is needed to understand FBXO42-CCDC-PPP4C's role in cancer cell survival in cell lines is needed before hypotheses about clinical implications can be developed. As such we feel this is outside the scope of the present manuscript.

Dear Dave,

Thank you for the submission of your revised manuscript EMBOR-2025-63099-T, 'The regulation of Protein Phosphatase 4 by FBXO42 is required for cancer cell survival' to EMBO reports.

We have meanwhile received a complete set of reviews from all referees, which I already forwarded to you, but let me repeat my summary here.

As you know all three referees consider the revised manuscript significantly strengthened but all three have remaining concerns. Most of them can be addressed by an extended discussion and by providing clarification. The referees however also ask for additional experiments. Referee #2 is concerned about the inconsistent levels of V5-IPed PPP4C and recommends repeating this experiment. This concerns is shared by Referee #3.

Referee #3 is not convinced that the data are sufficiently strong yet to fully support the proposed model of FBXO42 and CCDC6-mediated regulation of PPP4C stability and activity.
I think many of the concerns can be addressed in the discussion and by toning down conclusions but some of the experiments should be repeated and quantified (point 3 & 5).
Regarding Ref #3 point 8: we do not need a full mechanistic understanding at EMBO reports, but an assay for PP4 activity in a FBXL42 and CCDC6-dependent manner could be performed.

Overall, the proposed experiments seem feasible in a reasonable timeframe and you have already indicated in a separate e-mail that you can address these points.

We realize that it is difficult to revise to a specific deadline. In the interest of protecting the conceptual advance provided by the work, we recommend a revision within 3 months (15th Mar 2026). Please discuss the revision progress ahead of this time with the editor if you require more time to complete the revisions.

You will find the general formatting guidelines below, but I list here a few specific points that I noticed. Once your revised manuscript is in, we will perform a number of quality checks (Data availability, data description in figure legends, figure integrity screen, source data), so please make sure that this information is provided.

1) Statistical analysis may only be applied if the data are derived from at least three

biological repeats or independent experiments, which appears not to apply to Figure 2F, 5B, C.
2) Some figure legends refer to 'each point is a replicate well'. If these wells are from the same experiment, i.e., technical replicates, these should not be used for statistical analysis, as the number of independent experiments would be 1 (with three technical replicates).
Figure 3F lacks information on 'n'
3) Figure S# is an incorrect naming. Supplementary figures are either called 'Figure EV#' or bundled to an Appendix PDF (Appendix Fig. S#).
4) The mass spectrometry data need to be deposited in a public repository and the access information be specified in a dedicated 'Data Availability' section (see point 7 below). Please provide an URL that resolves directly to the dataset.
5) Please provide 5 keywords on the title page.
6) Please make sure that the funding information in the Acknowledgment section is congruent with the information in the online submission system. The latter counts and will be transferred to PubMed.

GENERAL FORMATTING GUIDELINES:

When submitting your revised manuscript, please review the submission guidelines (https://link.springer.com/journal/44319/submission-guidelines#cms-Revised-submissions) and carefully review the instructions that follow below. Failure to include requested items will delay the evaluation of your revision.

1) a .docx formatted version of the manuscript text (including legends for main figures, EV figures and tables). Please make sure that the changes are highlighted to be clearly visible.

2) individual production quality figure files as .eps, .tif, .jpg (one file per figure). See hhttps://media.springernature.com/original/springer-cms/rest/v1/content/27825798/data/v1 for more info on how to prepare your figures.

3) We replaced Supplementary Information with Expanded View (EV) Figures and Tables that are collapsible/expandable online. A maximum of 5 EV Figures can be typeset. EV Figures should be cited as 'Figure EV1, Figure EV2" etc... in the text and their respective legends should be included in the main text after the legends of regular figures.

- For the figures that you do NOT wish to display as Expanded View figures, they should be

bundled together with their legends in a single PDF file called *Appendix*, which should start with a short Table of Content. Appendix figures should be referred to in the main text as: "Appendix Figure S1, Appendix Figure S2" etc. See 'Expanded View' section of the submission guidelines.

- Additional Tables/Datasets should be labeled and referred to as Table EV1, Dataset EV1, etc. Legends have to be provided in a separate tab in case of .xls files. Alternatively, the legend can be supplied as a separate text file (README) and zipped together with the Table/Dataset file.

4) a .docx formatted letter INCLUDING the reviewers' reports and your detailed point-by-point responses to their comments. As part of the EMBO Press transparent editorial process, the point-by-point response is part of the Review Process File (RPF), which will be published alongside your paper.

5) a complete author checklist, which you can download from our author guidelines. Please insert information in the checklist that is also reflected in the manuscript. The completed author checklist will also be part of the RPF.

6) Please note that all corresponding authors are required to supply an ORCID ID for their name upon submission of a revised manuscript (<https://orcid.org/>), which can be linked to your profile in the manuscript submission system.

7) Before submitting your revision, primary datasets produced in this study need to be deposited in an appropriate public database. Please remember to provide a reviewer password if the datasets are not yet public. The accession numbers and database should be listed in a formal "Data Availability" section placed after Methods. Please note that the Data Availability Section is restricted to new primary data that are part of this study. * Note - All links should resolve to a page where the data can be accessed. *

If your study has not produced novel datasets, please mention this fact in the Data Availability Section.

8) At EMBO Press we ask authors to provide source data for the main manuscript figures. You will receive a separate email with instructions for providing source data with your revised manuscript, including how to upload and organize the files.

9) Our journal also encourages inclusion of *data citations in the reference list* to directly

cite datasets that were re-used and obtained from public databases. Data citations in the article text are distinct from normal bibliographical citations and should directly link to the database records from which the data can be accessed. In the main text, data citations are formatted as follows: "Data ref: Smith et al, 2001" or "Data ref: NCBI Sequence Read Archive PRJNA342805, 2017". In the Reference list, data citations must be labeled with "[DATASET]". A data reference must provide the database name, accession number/identifiers and a resolvable link to the landing page from which the data can be accessed at the end of the reference.

10) Regarding data quantification, the following points must be specified in each figure legend:

- the name of the statistical test used to generate error bars and P values,

- the number (n) of independent experiments (please specify technical or biological replicates) underlying each data point,

- the nature of the bars and error bars (s.d., s.e.m.),

- If the data are obtained from n {less than or equal to} 5, show the individual data points in addition to the SD or SEM.
- If the data are obtained from n {less than or equal to} 2, use scatter blots showing the individual data points.

Discussion of statistical methodology can be reported in the materials and methods section, but figure legends should contain a basic description of n, P and the test applied.

- Please also include scale bars in all microscopy images.

11) The journal requires a statement specifying whether or not authors have competing interests (defined as all potential or actual interests that could be perceived to influence the presentation or interpretation of an article). In case of competing interests, this must be specified in your disclosure statement.

12) All Materials and Methods need to be described in the main text using our 'Structured Methods' format, which is required for all research articles. According to this format, the Methods section includes a Reagents and Tools Table (listing key reagents, experimental

models, software and relevant equipment and including their sources and relevant identifiers) followed by a Methods and Protocols section describing the methods using a step-by-step protocol format. The aim is to facilitate adoption of the methodologies across labs. More information on how to adhere to this format as well as a downloadable template (.docx) for the Reagents and Tools Table can be found in our author guidelines.

We would also welcome the submission of cover suggestions, or motifs to be used by our Graphics Illustrator in designing a cover.

As part of the EMBO publication's Transparent Editorial Process, EMBO reports publishes online a Review Process File (RPF) to accompany accepted manuscripts. This File will be published in conjunction with your paper and will include the referee reports, your point-by-point response and all pertinent correspondence relating to the manuscript.

You are able to opt out of this by letting the editorial office know (emboreports@embo.org). If you do opt out, the Review Process File link will point to the following statement: "No Review Process File is available with this article, as the authors have chosen not to make the review process public in this case."

I look forward to seeing a revised form of your manuscript when it is ready.

Kind regards,

Martina

Martina Rembold, PhD
Senior Editor
EMBO reports

==================

Referee #1:

The authors have done a good job in focusing and revising the manuscript and it now has a very clear story. A few points that should be added to discussion:

1. The reported protein numbers (from HeLa cells) suggest 100 fold more CCDC6 than FBXO42. I think this adds some perspective on the occupancy of complexes:
Protein copy numbers from https://pubmed.ncbi.nlm.nih.gov/28601559/

CCDC6: 3,9 E+05
FBXO42: 4,8 E+03
PPP4R1: 7,9 E+04
PPP4R2: 1,7 E+05
PPP4C: 3,1 E+05
PPP4R4: 2,1 E+03
SMEK1/PPP4R3A: 9,4 E+04
SMEK2/PPP4R3B: 1,5 E+04

2. Although not thoroughly characterised PPP4R1 appears largely cytoplasmic (consistent with the reported localisation of the related PPP4R4) while PPP4R2 complexes are largely nuclear. Given the opposite localisation pattern the authors describe for CCDC6 and FBXO42 it is likely to speculate that CCDC6 largely acts on the PPP4R1-PP4C complex while FBXO42 might work on the nuclear complexes.

Referee #2:

The manuscript demonstrates that FBXO42 and CCDC6 form a complex with PPP4C independently, suppressing its activity. This dual inhibitory mechanism is crucial for cancer cell survival, as the loss of either FBXO42 or CCDC6 results in decreased viability or mitotic arrest, possibly through enhanced PPP4C activity. Although the manuscript has been further simplified and refined during revision, several shortcomings remain that warrant attention.

Comment:

In Figure 3D, the label "IP: FLAG" is incorrect and should be replaced with "IP: V5." The levels of V5-immunoprecipitated PPP4C protein are inconsistent, making it difficult to accurately assess the extent of PPP4C ubiquitination. This experiment should be repeated under optimized conditions, either by modifying the duration or concentration of MG132 treatment or by adjusting the amount of transfected PPP4C expression plasmid.

In Figures 4C and 4D, the data should be presented with an uncropped image to ensure

that the analysis was conducted on a single membrane.

There remains some uncertainty about the molecular basis of the co-dependency between FBXO42 and CCDC6, and the detailed regulatory mechanism by which CCDC6 inhibits PPP4C activity is still not fully defined. The data suggest that CCDC6 inhibits PPP4C through a mechanism that is distinct from FBXO42-mediated ubiquitination. While the authors confirm the critical residues and binding interface involved in the CCDC6-PPP4C interaction, the mechanism by which this binding attenuates PP4 activity remains to be elucidated. Furthermore, in CCDC6-knockout cells, a marked increase in PPP4C K48-linked ubiquitination was observed, potentially mediated by FBXO42 and/or other unidentified E3 ligases, while PPP4C protein levels remained largely unchanged in CCDC6-depleted (knockout and knockdown) cell lines.

Referee #3:

In this revised manuscript, the authors focus more explicitly on the functional relationship between CCDC6, FBXO42, and PPP4C. The stated motivation is to understand why FBXO42 is essential in certain cancer types. The authors identify PP4 complex subunits as major interaction partners of FBXO42 and now incorporate CCDC6 into the model, proposing that PPP4C interacts independently with both FBXO42 and CCDC6. They further argue that only loss of FBXO42 stabilizes PPP4C, whereas CCDC6 does not. Based on this, they conclude that increased PPP4C activity underlies the lethal effect associated with loss of both FBXO42 and CCDC6. While this explanation appears plausible for FBXO42, it is less convincing for CCDC6, as CCDC6 does not control PPP4C stability. The proliferation and competitive growth assays support a dependency on CCDC6, but for FBXO42 the evidence is weaker, partly because of variability in knockout efficiency.

Overall, the manuscript clarifies several aspects of the interaction network. However, it does not convincingly explain why FBXO42 is essential in a subset of cancer cells. The central claim that FBXO42 essentiality arises from PPP4C stabilization remains insufficiently supported. Although the data more convincingly show that CCDC6 essentiality depends on PPP4C, CCDC6 does not appear to regulate PPP4C stability, complicating the proposed model. A major missing experiment is a direct assay for PP4 activity in a CCDC6- and FBXO42-dependent manner; without such a readout, the mechanistic claims remain weak.

Detailed Comments

1. I do not understand the relevance of the observation that CCDC6 and FBXO42 knockout clones are more sensitive to PLK1 inhibition (Fig. 1D).
2. To improve figure readability, it would be helpful to indicate the exact positions of molecular weight markers on all immunoblots.
3. The claim "We observed a reduction in the polyubiquitin smear associated with PPP4C, but not PPP4R1 or PPP4R2, in MLN4924-treated cells (Figure 3A)" is not sufficiently supported by the data. This experiment should be repeated at least twice more, quantified, and statistically evaluated. Alternatively, the authors should tone down the claim.
4. The statement "Interestingly, CCDC6-/- cells had increased PPP4C ubiquitination, through a still unknown mechanism" is not supported by the quantification in Figure 3B-C. The authors should moderate this claim.
5. In Figure 3D, the FLAG IP signal appears markedly stronger in CCDC6 KO cells and highly variable across cell lines, in contrast to the more consistent IP shown in Figure 3B. It is also unclear whether the experiment is a FLAG IP (as indicated in the figure) or a V5 IP (as written in the text). This discrepancy should be resolved. After clarification, the experiment should be repeated and quantified, as in Figure 3B/C.
6. The shRNA constructs used in Figure 4C are not clearly indicated. Figures 4A and 4B show two constructs per gene plus a control, yet Figure 4C contains four lanes. The authors should specify which constructs were used.
7. FBXO42 knockdown efficiency appears reduced when combined with PPP4C knockdown. For FBXO42, this reduced knockdown efficiency could itself explain the improved proliferation. This limitation should be discussed.
8. The authors claim that the essentiality of CCDC6 results from hyperactivation of PP4, but they do not provide a plausible mechanism for how CCDC6 knockdown would cause increased PP4 activity. A direct biochemical or functional assay measuring PP4 activity in the different genetic backgrounds would substantially strengthen the manuscript.

Raw data for all proteomics experiments has been deposited at MassIVE. ID: **MSV000100795**. Password for pre-publication access: Unavailable

Referee #1:

The authors have done a good job in focusing and revising the manuscript and it now has a very clear story. A few points that should be added to discussion:
1. The reported protein numbers (from HeLa cells) suggest 100 fold more CCDC6 than FBXO42. I think this adds some perspective on the occupancy of complexes:
Protein copy numbers from https://pubmed.ncbi.nlm.nih.gov/28601559/

CCDC6: 3,9 E+05
FBXO42: 4,8 E+03
PPP4R1: 7,9 E+04
PPP4R2: 1,7 E+05
PPP4C: 3,1 E+05
PPP4R4: 2,1 E+03
SMEK1/PPP4R3A: 9,4 E+04
SMEK2/PPP4R3B: 1,5 E+04

We thank the reviewer for this insightful observation. We have added the following text to the Results and Discussion section (changes underlined):

> "In summary, we present data showing that CCDC6 and FBXO42 inhibit PPP4C in different ways: FBXO42 by targeting PPP4C for turnover, and CCDC6 by an as of yet not understood, but previously characterized, mechanism (Morra et al., 2022) (Figure 5F). <u>It has been reported that CCDC6 directly binds PPP4C and reduces its catalytic activity, but the exact means by which it does this is not understood (Merolla et al., 2012). Notably, according to proteomics studies in HeLa cells, CCDC6 and PPP4C are present in the cell in similar copy numbers, consistent with direct inhibition, whereas FBXO42 is about 100-fold less abundant, as expected for a catalytic role (Bekker-Jensen et al., 2017). It will be important for future studies to determine what portion of cellular PPP4C interacts with CCDC6 at a given point in time, what regulates this interaction, and with which PP4 holoenzymes CCDC6 interacts</u>."

2. Although not thoroughly characterised PPP4R1 appears largely cytoplasmic

(consistent with the reported localisation of the related PPP4R4) while PPP4R2 complexes are largely nuclear. Given the opposite localisation pattern the authors describe for CCDC6 and FBXO42 it is likely to speculate that CCDC6 largely acts on the PPP4R1-PP4C complex while FBXO42 might work on the nuclear complexes.

We thank the reviewer for this helpful recommendation. We have updated the discussion with the following text to reflect these points:

> "This observation also highlights that, while there is significant overlap in FBXO42 and CCDC6 function, they are not redundant and may be regulated through different mechanisms. For example, they could function in different cellular compartments, as reportedly FBXO42 is mostly nuclear, CCDC6 is cytoplasmic but travels to the nucleus, and PP4 localizes more to the nucleus but is clearly detected in the cytosol (Ueki et al., 2019; Uhlén et al., 2015; Wu et al., 2022). It is also possible that they bind different PP4 holoenzymes. We observed FBXO42 immunoprecipitating PPP4R2 and PPP4R1 (Figure 1A), but CCDC6 only immunoprecipitating PPP4R1 (Figure 2A, 5E). PPP4R2 is reported to be nuclear whereas PPP4R1 was shown to be cytoplasmic (Thul et al., 2017). Conversely, other studies have found CCDC6 interacting with PPP4R2, PPP4R3A, and PPP4R3B (Huttlin et al., 2021; Huttlin et al., 2017; Lü et al., 2024; Oughtred et al., 2021). It has been reported that CCDC6 translocates to the nucleus in response to stimuli such as DNA damage or serum starvation (Celetti et al., 2004; Merolla et al., 2007). Since these stimuli were not applied to the RPE1 cells we used to study CCDC6 interactions, it is possible that CCDC6 was largely cytoplasmic at the time and as a result associated more with PPP4C-PPP4R1.

We have also added new data showing that CCDC6 immunoprecipitates PPPR1, but not PPP4R2, PPP4R3A, or PPP4R3B in the conditions used in our study, see revised Figure 5E.

Referee #2:

The manuscript demonstrates that FBXO42 and CCDC6 form a complex with PPP4C independently, suppressing its activity. This dual inhibitory mechanism is crucial for cancer cell survival, as the loss of either FBXO42 or CCDC6 results in decreased viability or mitotic arrest, possibly through enhanced PPP4C activity. Although the manuscript has been further simplified and refined during revision, several shortcomings remain that warrant attention.

Comment:

In Figure 3D, the label "IP: FLAG" is incorrect and should be replaced with "IP: V5." The levels of V5-immunoprecipitated PPP4C protein are inconsistent, making it difficult to accurately assess the extent of PPP4C ubiquitination. This experiment should be repeated under optimized conditions, either by modifying the duration or concentration of MG132 treatment or by adjusting the amount of transfected PPP4C expression plasmid.

We thank the reviewer for this comment. We have corrected this labeling mistake. We also repeated the experiment with more consistent levels of PPP4C-V5 immunoprecipitated (revised figure 3D, 3E). Multiple repeats are quantified. Ubiquitination levels are normalized to the amounts of PPP4C-V5 that were immunoprecipitated and to levels of Flag-Ubiquitin expression to reduce to impact of these sources of error.

In Figures 4C and 4D, the data should be presented with an uncropped image to ensure that the analysis was conducted on a single membrane.

We thank the reviewer for this recommendation. To remove any chance of ambiguity we have repeated the blots, and provide new, uncropped images (revised Figure 4C and 4D). Accompanying quantification is also provided. The new blots also include additional control samples.

There remains some uncertainty about the molecular basis of the co-dependency between FBXO42 and CCDC6, and the detailed regulatory mechanism by which CCDC6 inhibits PPP4C activity is still not fully defined. The data suggest that CCDC6 inhibits PPP4C through a mechanism that is distinct from FBXO42-mediated ubiquitination. While the authors confirm the critical residues and binding interface involved in the CCDC6-PPP4C interaction, the mechanism by which this binding attenuates PP4 activity remains to be elucidated. Furthermore, in CCDC6-knockout cells, a marked increase in PPP4C K48-linked ubiquitination was observed, potentially mediated by FBXO42 and/or other unidentified E3 ligases, while PPP4C protein levels remained largely unchanged in CCDC6-depleted (knockout and knockdown) cell lines.

We thank the reviewer for these insightful comments. A previous study (Merolla et al., 2012) found that CCDC6 inhibits PP4 activity through direct interaction. This current manuscript does not determine how CCDC6 interaction with PPP4C attenuates PP4

activity, but we feel that determining the details of this mechanism should be investigated in future studies. We have added text to the Results and Discussion section to highlight these limitations and propose areas for future work.

"We hypothesized that FBXO42 and CCDC6 negatively regulate the activity of PP4, and that the loss of this regulation is responsible for their essentiality in some cell lines, such as the glioblastoma line LN-18. CCDC6 has been previously reported to directly interact with PP4 and inhibit its activity in direct, in-vitro phosphatase assays (Merolla et al., 2012). We analyzed the viability and growth of LN-18 cells transduced with shRNA against FBXO42 or CCDC6 using a flow-cytometry-based competitive growth assay."

. . .

"Cell lines such as LN-18 may be selectively dependent upon FBXO42 and CCDC6 because they have intrinsic mitotic defects or increased reliance on a kinase opposed by PP4. Interestingly, knockdown of PPP4C was observed to slightly increase FBXO42 expression (Figure EV3A). The possibility that partial rescue of FBXO42 expression contributes to rescued viability in cells with both FBXO42 and PPP4C knocked down (Figure 5A, 5B) cannot be excluded. These findings mirror those from a recent study that found that depletion of PP4 rescued another FBXO42 depletion associated phenotype, in this case sensitivity to DNA damage (Yang et al., 2026). This study was performed in cells where FBXO42 was depleted at the genetic level, making rescue of the phenotype by increased FBXO42 expression unlikely."

. . .

""In summary, we present data showing that CCDC6 and FBXO42 inhibit PPP4C in different ways: FBXO42 by targeting PPP4C for turnover, and CCDC6 by an as of yet not understood, but previously characterized, mechanism (Morra et al., 2022) (Figure 5F). It has been reported that CCDC6 directly binds PPP4C and reduces its catalytic activity, but the exact means by which it does this is not understood (Merolla et al., 2012). Notably, according to proteomics studies in HeLa cells, CCDC6 and PPP4C are present in the cell in similar copy numbers, consistent with direct inhibition, whereas FBXO42 is about 100-fold less abundant, as expected for a catalytic role (Bekker-Jensen et al., 2017). It will be important for future studies to determine what portion of cellular PPP4C interacts with CCDC6 at a given point in time, what regulates this interaction, and with which PP4 holoenzymes CCDC6 interacts."

Significantly, a parallel study published concurrently with this one found that FBXO42 binds to and negatively regulates PPP4C (Yang et al., 2026) . The authors of that study also found that FBXO42 ubiquitinates PPP4C, and that PPP4C levels are increased in FBXO42 depleted cells, but, in contrast to our conclusions, the authors conclude that ubiquitination of PPP4C is primarily non-degradative and serves to modulate PP4 catalytic activity (Yang et al., 2026)."

Having performed additional replicates of V5 IPs testing PPP4C-V5 ubiquitination in $FBXO42^{-/-}$ and $CCDC6^{-/-}$ cells, we see that the small increase observed in PPP4C ubiquitination in $CCDC6^{-/-}$ cells was variable and not consistently higher than in wildtype cells, as confirmed by new quantitation (see revised Figure 3E). While some experiments showed a decrease in PPP4C levels in $CCDC6^{-/-}$ cells (see Figure 4D), we did not see this decrease with the same consistency that we saw the increase in PPP4C expression in $FBXO42^{-/-}$ cells, which we observed across multiple experiments performed different ways (with different cell lines, with genetic knockout and shRNA knockdown). Therefore, we feel that further investigation is needed to determine if CCDC6 meaningfully affects PPP4C ubiquitination, and that this can be explored in future publications.

Referee #3:

In this revised manuscript, the authors focus more explicitly on the functional relationship between CCDC6, FBXO42, and PPP4C. The stated motivation is to understand why FBXO42 is essential in certain cancer types. The authors identify PP4 complex subunits as major interaction partners of FBXO42 and now incorporate CCDC6 into the model, proposing that PPP4C interacts independently with both FBXO42 and CCDC6. They further argue that only loss of FBXO42 stabilizes PPP4C, whereas CCDC6 does not. Based on this, they conclude that increased PPP4C activity underlies the lethal effect associated with loss of both FBXO42 and CCDC6. While this explanation appears plausible for FBXO42, it is less convincing for CCDC6, as CCDC6 does not control PPP4C stability. The proliferation and competitive growth assays support a dependency on CCDC6, but for FBXO42 the evidence is weaker, partly because of variability in knockout efficiency.

Overall, the manuscript clarifies several aspects of the interaction network. However, it does not convincingly explain why FBXO42 is essential in a subset of cancer cells. The central claim that FBXO42 essentiality arises from PPP4C stabilization remains

insufficiently supported. Although the data more convincingly show that CCDC6 essentiality depends on PPP4C, CCDC6 does not appear to regulate PPP4C stability, complicating the proposed model. A major missing experiment is a direct assay for PP4 activity in a CCDC6- and FBXO42-dependent manner; without such a readout, the mechanistic claims remain weak.

We thank the reviewer for these important comments, and apologize for not spelling out published in vitro data more explicitly. It has been previously reported that CCDC6 inhibits PP4 activity through direct interaction in vitro (Merolla et al, 2012). These experiments included in-vitro phosphatase assays. While the exact mechanism by which CCDC6 inhibits PP4 activity remains to be elucidated, we feel that our model is sufficiently well-supported for publication, based on the strength of our genetic data, the direct binding of CCDC6 and PPP4C, our phospho-proteomics showing a strong correlation between FBXO42 and CCDC6, the additivity of their elimination on cell growth and since it is consistent with previous literature. We believe that in light of the previous report that CCDC6 directly reduces PP4 activity, we do not feel that the fact that CCDC6 does not regulate PPP4C stability compromises our model. We have added the following text to reflect this:

> "We hypothesized that FBXO42 and CCDC6 negatively regulate the activity of PP4, and that the loss of this regulation is responsible for their essentiality in some cell lines, such as the glioblastoma line LN-18. CCDC6 has been previously reported to directly interact with PP4 and inhibit its activity, including in direct, in-vitro phosphatase assays (Merolla et al., 2012). We analyzed the viability and growth of LN-18 cells transduced with shRNA against FBXO42 or CCDC6 using a flow-cytometry-based competitive growth assay."

While knockdown of PPP4C had a more dramatic effect in rescuing viability in the context of CCDC6 knockdown than FBXO42 knockdown, we feel that since we consistently observed rescue of FBXO42 related cell death/arrest, the results are robust. Moreover, there is no a priori reason to believe that the FBXO42 and CCDC6 contribute equally to reducing PP4 activity. Data from additional replicate experiments showing rescue of FBXO42 knockdown by PP4C have been added as Extended View 3C and 3D.

Detailed Comments

1. I do not understand the relevance of the observation that CCDC6 and FBXO42 knockout clones are more sensitive to PLK1 inhibition (Fig. 1D).

We thank the reviewer for this comment. We feel that this finding is relevant because it provides additional evidence that FBXO42 and CCDC6 have similar functions within the

cell, as suggested by their close correlation in DepMap dependencies. In turn, this similarity justifies examining the similarities and differences in FBXO42 and CCDC6 interactions and functions that follow in the manuscript. Moreover, our hypothesis that loss of FBXO42 or CCDC6 causes death by loss of an essential PP4-targeted phosphorylation, combined with mitotic defects we and others have previously reported upon loss of FBXO42, make the shared sensitivity to a mitotic kinase important. We have added some text to make this rationale clearer to the reader. (New text underlined)

> "CCDC6 exhibits a high co-dependency correlation with FBXO42 in terms of cell line essentiality (Figure 1B), which is commonly observed for genes which serve a similar cellular function or are in the same pathway. Both FBXO42$^{-/-}$ and CCDC6$^{-/-}$ RPE1 cells (Figure 1C, S1) had increased sensitivity to the PLK1 inhibitor BI-2536 compared to WT cells (Figure 1D), consistent with previous results for FBXO42 in HAP1 cells (Hundley et al., 2021). In contrast, they did not differ from wild-type cells in sensitivity to rotenone, which kills cells via an unrelated mechanism. The increased sensitivity of CCDC6$^{-/-}$ cells is consistent with the hypothesis that FBXO42 and CCDC6 play similar functional roles in cells."

2. To improve figure readability, it would be helpful to indicate the exact positions of molecular weight markers on all immunoblots.

We thank the reviewer for this recommendation. Immunoblots have been updated with clearer molecular weight markers.

3. The claim "We observed a reduction in the polyubiquitin smear associated with PPP4C, but not PPP4R1 or PPP4R2, in MLN4924-treated cells (Figure 3A)" is not sufficiently supported by the data. This experiment should be repeated at least twice more, quantified, and statistically evaluated. Alternatively, the authors should tone down the claim.

We thank the reviewer for this insightful comment. We have changed the text to tone down the claim and make it clear that these data alone should not be used to make conclusions about ubiquitination of PP4 subunits:

> "While significant polyubiquitination smears were observed for all three proteins, it appeared that the smear associated with PPP4C was slightly reduced with MLN4924 treatment (Figure 3A). Since this suggested that PPP4C is

ubiquitinated by a cullin ring ligase (CRL), we focused our investigation on the role of FBXO42 in PPP4C ubiquitination. However, further investigation is warranted into ubiquitination of PPP4R1 and PPP4R2, by CRLs or other E3 ligases."

4. The statement "Interestingly, CCDC6-/- cells had increased PPP4C ubiquitination, through a still unknown mechanism" is not supported by the quantification in Figure 3B-C. The authors should moderate this claim.

We thank the reviewer for this comment. We agree that increased ubiquitination of PPP4C in CCDC6$^{-/-}$ cells is not well supported, especially after we performed additional replicates of these experiments (see revised Figure 3D and 3E). For reasons we do not entirely understand, PP4C ubiquitination levels are highly variable in CCDC6 mutants. We have removed this claim.

5. In Figure 3D, the FLAG IP signal appears markedly stronger in CCDC6 KO cells and highly variable across cell lines, in contrast to the more consistent IP shown in Figure 3B. It is also unclear whether the experiment is a FLAG IP (as indicated in the figure) or a V5 IP (as written in the text). This discrepancy should be resolved. After clarification, the experiment should be repeated and quantified, as in Figure 3B/C.

We thank the reviewer for this insightful comment. We have corrected mistakes in the labeling of the figures. We also have performed additional repeats of the V5 IP where expression levels of PPP4C-V5 were more consistent. We have also quantified the repeats. See revised Figure 3D and 3E.

6. The shRNA constructs used in Figure 4C are not clearly indicated. Figures 4A and 4B show two constructs per gene plus a control, yet Figure 4C contains four lanes. The authors should specify which constructs were used.

We thank the reviewer for this comment. We have clarified figure labelling to make it clear which construct was used in which experiments. See revisions to Figure 4C, 4D, and to the corresponding figure legends.

7. FBXO42 knockdown efficiency appears reduced when combined with PPP4C knockdown. For FBXO42, this reduced knockdown efficiency could itself explain the improved proliferation. This limitation should be discussed.

We thank the reviewer for this salient observation. We have added text addressing this limitation. We also contextualize the data in this manuscript with a related experiment in Yang *et al,* 2026.

> "Cell lines such as LN-18 may be selectively dependent upon FBXO42 and CCDC6 because they have intrinsic mitotic defects or increased reliance on a kinase opposed by PP4. Interestingly, under some conditions, knockdown of PPP4C was observed to slightly increase FBXO42 expression (Figure 5A). The possibility that partial rescue of FBXO42 expression contributes to rescued viability in cells with both FBXO42 and PPP4C knocked down (Figure 5B, 5C) cannot be excluded. These findings mirror those from a recent study that found that depletion of PP4 rescued another FBXO42 depletion associated phenotype, in this case sensitivity to DNA damage (Yang et al., 2026). This study was performed in cells where FBXO42 was depleted at the genetic level, making rescue of the phenotype by increased FBXO42 expression unlikely.

8. The authors claim that the essentiality of CCDC6 results from hyperactivation of PP4, but they do not provide a plausible mechanism for how CCDC6 knockdown would cause increased PP4 activity. A direct biochemical or functional assay measuring PP4 activity in the different genetic backgrounds would substantially strengthen the manuscript.

We thank the reviewer for this insightful comment and apologize for not clearly noting that this has been performed previously. A previous study of CCDC6 (Merolla et al. 2012) showed that CCDC6 directly interacts with PP4 and reduces its activity. While further study of the way different mutations to CCDC6 affect PP4 activity would be a valuable direction for future research, we believe that referencing the previously established ability of CCDC6 to inhibit PP4 is sufficient for this current manuscript. We have added the following text to reflect this:

> "We hypothesized that FBXO42 and CCDC6 negatively regulate the activity of PP4, and that the loss of this regulation is responsible for their essentiality in some cell lines, such as the glioblastoma line LN-18. CCDC6 has been previously reported to directly interact with PP4 and inhibit its activity, including in direct, in-vitro phosphatase assays (Merolla et al., 2012). We analyzed the viability and growth of LN-18 cells transduced with shRNA against FBXO42 or CCDC6 using a flow-cytometry-based competitive growth assay."

1st Revision - Editorial Decision 16th Apr 2026

Dear Dr. Toczyski

Thank you for the submission of your revised manuscript to EMBO reports. We have now received feedback from former referee #2 and #3 (copied below). As you will see, both referees recommend publication.

Before I can accept the manuscript, I need you to address some minor points below:

- Please clearly label the corresponding author and provide the e-mail address on the title page. Please note that all corresponding authors must have an insitutional e-mail address. The private e-mail address can only be listed as secondary address.

- All corresponding authors must provide an ORCID ID, which should be linked to the profile in the manuscript submission system. This is currently missing.

- Affiliation #7, Acrobat Genomics, San Carlos, CA, USA, appears to refer to a biotech company. If so, please disclose the affiliation to a for-profit company in the Disclosure and Competing Interests statement.

- Regarding the Author Contributions, we now use CRediT to specify the contributions of each author in the journal submission system. CRediT replaces the author contribution section, which therefore needs to be removed from the manuscript text. You can use the free text box in our system if you wish to provide more detailed descriptions. See also guide to authors https://www.embopress.org/page/journal/14693178/authorguide#authorshipguidelines.

- The information on funding provided in the manuscript and the online submission system must be congruent. Here we note that the UCSF Program for Breakthrough Biomedical Research (funded in part by the Sandler Foundation) is acknowledged in the manuscript but not entered in the system.

- The manuscript sections should be in the following order: Title page - Abstract & Keywords - Introduction - Results - Discussion - Methods - Data Availability - Acknowledgments - Disclosure Statement & Competing Interests - References - Figure Legends - (Main Tables with legends if applicable) - Expanded View Figure Legends.

- Materials and Methods should be Methods

- Data availability section: Please add the specific URL for MSV000100795 dataset and please remove the following statement as this section should only refer to datasets deposited at public repositories: "Cell lines and DNA constructs generated in this study are available from the authors by request."

- Please remove the Reagents and Tools table from the manuscript. The uploaded copy of the R&T table will be used for typesetting.

- Author Checklist: please add the manuscript ID in C6.

- There is a callout to Figure S2 in the methods section. Should this be a reference to Figure EV2 instead?

- Appendix Table S1 is a dataset and needs to be updated to Dataset EV1 in all places. Please also provide a legend in a separate tab of the .xls file. I could not find a reference to the table in the manuscript text. Please make sure to add a callout.

- Please define the error bars in the legends of figures 4A, B

- I have introduced a few minor changes in the abstract (copied below) that I kindly ask you to review.

- Finally, EMBO Reports papers are accompanied online by
A) a short (1-2 sentences) summary of the findings and their significance,
B) 2-3 bullet points highlighting key results and
C) a schematic summary figure that provides a sketch of the major findings (not a data image).
Please provide the summary figure as a separate file in PNG or JPG format at a size of 550x300 pixels (width x height). Please note that the size is rather small and that text needs to be readable at the final size. Please send us this information along with the revised manuscript.

We look forward to seeing a final version of your manuscript as soon as possible.

With kind regards,

Martina

Martina Rembold, PhD
Senior Editor
EMBO reports

=================

Referee #2:

After multiple rounds of revision, the manuscript presents a clear and convincing story. While additional mechanistic evidence could further strengthen the proposed model, the current data are sufficient to support the conclusions. The manuscript is suitable for publication in EMBO Reports.

Referee #3:

The authors have substantially improved the manuscript, comprehensively addressed all of my comments, and clarified the previously unclear points.

===============

Abstract

FBXO42 is a poorly characterized F-box protein essential in 15% of cancer cell lines from diverse lineages. High-throughput approaches indicate that FBXO42 function correlates with that of coiled-coil protein CCDC6, and that the two proteins interact physically, but the relationship between them is not understood. We show that FBXO42 ubiquitinates PPP4C (protein phosphatase 4 catalytic subunit) and negatively regulates its expression. FBXO42 binds PPP4C independently of CCDC6. Similarly, we show that CCDC6 physically interacts with PPP4C independently of FBXO42, and identify a PPP4C binding site within CCDC6. However, mutation of CCDC6 does not reduce PPP4C ubiquitination, suggesting that FBXO42 and CCDC6 bind and regulate PPP4C through separate mechanisms. Viability of an FBXO42- and CCDC6-dependent glioblastoma cell line is rescued when PPP4C is knocked down along with FBXO42 or CCDC6, suggesting that aberrant activation of PPP4C is a driver of cell death when FBXO42 or CCDC6 activity is compromised. These findings tie together previous data suggesting that FBXO42 functions with CCDC6 by providing mechanistic insight into their independent regulation of PP4.

We thank the reviewers and editors for their time and effort in reviewing this manuscript. We now resubmit the manuscript with the minor changes described below completed.

Dear Dr. Toczyski

Thank you for the submission of your revised manuscript to EMBO reports. We have now received feedback from former referee #2 and #3 (copied below). As you will see, both referees recommend publication.

Before I can accept the manuscript, I need you to address some minor points below:

- Please clearly label the corresponding author and provide the e-mail address on the title page. Please note that all corresponding authors must have an insitutional e-mail address. The private e-mail address can only be listed as secondary address.

The corresponding author has been labeled, and the institutional email address has been added to the title page.

- All corresponding authors must provide an ORCID ID, which should be linked to the profile in the manuscript submission system. This is currently missing.

We will address this in the submission system.

- Affiliation #7, Acrobat Genomics, San Carlos, CA, USA, appears to refer to a biotech company. If so, please disclose the affiliation to a for-profit company in the Disclosure and Competing Interests statement.

We apologize for the omission and have added this information to the Disclosure and Competing Interest statement. The author who now works at Acrobat Genomics confirms that they have no competing interests beyond the fact that they are employed by a for-profit company.

- Regarding the Author Contributions, we now use CRediT to specify the contributions of each author in the journal submission system. CRediT replaces the author contribution section, which therefore needs to be removed from the manuscript text. You can use the free text box in our system if you wish to provide more detailed descriptions. See also guide to authors https://www.embopress.org/page/journal/14693178/authorguide#authorshipguideline

s.
The section has been removed form the manuscript text.

- The information on funding provided in the manuscript and the online submission system must be congruent. Here we note that the UCSF Program for Breakthrough Biomedical Research (funded in part by the Sandler Foundation) is acknowledged in the manuscript but not entered in the system.

We thank the editor for spotting this oversight, and will correct it in the submission system.

- The manuscript sections should be in the following order: Title page - Abstract & Keywords - Introduction - Results - Discussion - Methods - Data Availability - Acknowledgments - Disclosure Statement & Competing Interests - References - Figure Legends - (Main Tables with legends if applicable) - Expanded View Figure Legends.

The order has bene corrected.

- Materials and Methods should be Methods

Done

- Data availability section: Please add the specific URL for MSV000100795 dataset and please remove the following statement as this section should only refer to datasets deposited at public repositories: "Cell lines and DNA constructs generated in this study are available from the authors by request."

We have updated the section with the URL and removed the described text.

- Please remove the Reagents and Tools table from the manuscript. The uploaded copy of the R&T table will be used for typesetting.

Done.

- Author Checklist: please add the manuscript ID in C6.

Done.

- There is a callout to Figure S2 in the methods section. Should this be a reference to Figure EV2 instead?

It was meant to be Figure EV1 in the final draft. We thank the editor for identifying this error and we have corrected it.

- Appendix Table S1 is a dataset and needs to be updated to Dataset EV1 in all places. Please also provide a legend in a separate tab of the .xls file. I could not find a reference to the table in the manuscript text. Please make sure to add a callout.

We have renamed the table to Dataset EV1 and added a callout to it in the relevant location in the text. We have also added a legend in a separate tab in the .xls file. A legend is also provided in the text at the end of the EV Figure legends but that can be removed if it is not needed.

- Please define the error bars in the legends of figures 4A, B

We have added additional text to define these bars.

- I have introduced a few minor changes in the abstract (copied below) that I kindly ask you to review.

We approve of the changes to the abstract and have updated it in the text.

- Finally, EMBO Reports papers are accompanied online by
A) a short (1-2 sentences) summary of the findings and their significance,
B) 2-3 bullet points highlighting key results and
C) a schematic summary figure that provides a sketch of the major findings (not a data image). Please provide the summary figure as a separate file in PNG or JPG format at a size of 550x300 pixels (width x height). Please note that the size is rather small and that text needs to be readable at the final size. Please send us this information along with the revised manuscript.

Summary sentence: The E3 ubiquitin ligase FBXO42 and the coiled-coiled domain-containing protein CCDC6 both negatively regulate PPP4C, and this target is critical for their essential role in some tumor cell lines. Despite this common function, FBXO42 and CCDC6 bind and regulate PPP4C through separate mechanisms.

Bullet Point Key Results:

- FBXO42 ubiquitinates PPP4C, negatively regulating its expression. CCDC6 binds to and negatively regulates PPP4C independently of FBXO42.
- In a cancer cell line where FBXO42 and CCDC6 are essential, aberrant PPP4C activity contribute to cell death following FBXO42 or CCDC6 depletion.

- CCDC6 binds to PPP4C through a repeated EXXXN motif.

The summary figure will be submitted through the manuscript submission system.

Dr. David Toczyski
University of California, San Francisco
Department of Biochemistry and Biophysics
San Francisco, CA
United States

Dear Dave,

Thank you for implementing the final minor edits. I am very pleased to accept your manuscript for publication in the next available issue of EMBO reports. Thank you for your contribution to our journal.

Your manuscript will be processed for publication by EMBO Press. It will be copy edited and you will receive page proofs prior to publication. Please note that you will be contacted by Springer Nature Author Services to complete licensing and payment information.

You may qualify for financial assistance for your publication charges - either via a Springer Nature fully open access agreement or an EMBO initiative. Check your eligibility: https://link.springer.com/journal/44319/how-to-publish-with-us

Should you be planning a Press Release on your article, please notify the production team via this form in order to coordinate publication and release dates: https://forms.cloud.microsoft/e/eH4A8JVP6g

If you have any questions, please do not hesitate to contact the Editorial Office. Thank you for your contribution to EMBO Reports.

Kind regards,

Martina

Martina Rembold, PhD
Senior Editor
EMBO reports

>>> Please note that it is EMBO Reports policy for the transcript of the editorial process (containing referee reports and your response letter) to be published as an online supplement to each paper. If you do NOT want this, you will need to inform the Editorial Office via email immediately. More information is available here: https://link.springer.com/partners/embo-press/editorial-policies#Peer%20review

EMBO Press Author Checklist

Corresponding Author Name: Stephan H Spangenberg	
Journal Submitted to: EMBO Reports	
Manuscript Number: EMBOR-2025-63099V3	

USEFUL LINKS FOR COMPLETING THIS FORM
The EMBO Journal - Author Guidelines
EMBO Reports - Author Guidelines
Molecular Systems Biology - Author Guidelines
EMBO Molecular Medicine - Author Guidelines

Reporting Checklist for Life Science Articles (updated January
This checklist is adapted from Materials Design Analysis Reporting (MDAR) Checklist for Authors. MDAR establishes a minimum set of requirements in transparent reporting in the life sciences (see Statement of Task: 10.31222/osf.io/9sm4x). Please follow the journal's guidelines in preparing your
Please note that a copy of this checklist will be published alongside your article.

Abridged guidelines for figures
1. Data
The data shown in figures should satisfy the following conditions:
- the data were obtained and processed according to the field's best practice and are presented to reflect the results of the experiments in an accurate and unbiased manner.
- ideally, figure panels should include only measurements that are directly comparable to each other and obtained with the same assay.
- plots include clearly labeled error bars for independent experiments and sample sizes. Unless justified, error bars should not be shown for technical
- if n<5, the individual data points from each experiment should be plotted. Any statistical test employed should be justified.
- Source Data should be included to report the data underlying figures according to the guidelines set out in the authorship guidelines on Data

2. Captions
Each figure caption should contain the following information, for each panel where they are relevant:
- a specification of the experimental system investigated (eg cell line, species name).
- the assay(s) and method(s) used to carry out the reported observations and measurements.
- an explicit mention of the biological and chemical entity(ies) that are being measured.
- an explicit mention of the biological and chemical entity(ies) that are altered/varied/perturbed in a controlled manner.
- the exact sample size (n) for each experimental group/condition, given as a number, not a range;
- a description of the sample collection allowing the reader to understand whether the samples represent technical or biological replicates (including how many animals, litters, cultures, etc.).
- a statement of how many times the experiment shown was independently replicated in the laboratory.
- definitions of statistical methods and measures:
  - common tests, such as t-test (please specify whether paired vs. unpaired), simple χ2 tests, Wilcoxon and Mann-Whitney tests, can be unambiguously identified by name only, but more complex techniques should be described in the methods section;
  - are tests one-sided or two-sided?
  - are there adjustments for multiple comparisons?
  - exact statistical test results, e.g., P values = x but not P values < x;
  - definition of 'center values' as median or average;
  - definition of error bars as s.d. or s.e.m.

Please complete ALL of the questions below.
Select "Not Applicable" only when the requested information is not relevant for your study.

Materials

Newly Created Materials	Information included in the manuscript?	In which section is the information available? (Reagents and Tools Table, Materials and Methods, Figures, Data Availability Section)
New materials and reagents need to be available; do any restrictions apply?	Yes	Data Availability

Antibodies	Information included in the manuscript?	In which section is the information available? (Reagents and Tools Table, Materials and Methods, Figures, Data Availability Section)
For **antibodies** provide the following information:<br>- Commercial antibodies: RRID (if possible) or supplier name, catalogue number and or/clone number<br>- Non-commercial: RRID or citation	Yes	Reagents and Tools Table

DNA and RNA sequences	Information included in the manuscript?	In which section is the information available? (Reagents and Tools Table, Materials and Methods, Figures, Data Availability Section)
**Short novel DNA or RNA including primers, probes**: provide the sequences.	Yes	Reagents and Tools Table, and Methods

Cell materials	Information included in the manuscript?	In which section is the information available? (Reagents and Tools Table, Materials and Methods, Figures, Data Availability Section)
**Cell lines:** Provide species information, strain. Provide accession number in repository **OR** supplier name, catalog number, clone number, and/**OR** RRID.	Yes	Reagents and Tools Table, and Materials and Methods
**Primary cultures:** Provide species, strain, sex of origin, genetic modification status.	Not Applicable	
Report if the cell lines were recently **authenticated** (e.g., by STR profiling) and tested for mycoplasma contamination.	Yes	Materials and Methods

Experimental animals	Information included in the manuscript?	In which section is the information available? (Reagents and Tools Table, Materials and Methods, Figures, Data Availability Section)
**Laboratory animals or Model organisms:** Provide species, strain, sex, age, genetic modification status. Provide accession number in repository **OR** supplier name, catalog number, clone number, **OR** RRID.	Not Applicable	
**Animal observed in or captured from the field:** Provide species, sex, and age where possible.	Not Applicable	
Please detail **housing and husbandry conditions**.	Not Applicable	

Plants and microbes	Information included in the manuscript?	In which section is the information available? (Reagents and Tools Table, Materials and Methods, Figures, Data Availability Section)
**Plants:** provide species and strain, ecotype and cultivar where relevant, unique accession number if available, and source (including location for collected wild specimens).	Not Applicable	
**Microbes:** provide species and strain, unique accession number if available, and source.	Not Applicable	

Human research participants	Information included in the manuscript?	In which section is the information available? (Reagents and Tools Table, Materials and Methods, Figures, Data Availability Section)
If collected and within the bounds of privacy constraints report on age, sex and gender or ethnicity for all study participants.	Not Applicable	

Core facilities	Information included in the manuscript?	In which section is the information available? (Reagents and Tools Table, Materials and Methods, Figures, Data Availability Section)
If your work benefited from core facilities, was their service mentioned in the acknowledgments section?	Yes	Acknowledgements

Design

Study protocol	Information included in the manuscript?	In which section is the information available? (Reagents and Tools Table, Materials and Methods, Figures, Data Availability Section)
If study protocol has been **pre-registered, provide DOI in the manuscript**. For clinical trials, provide the trial registration number **OR** cite DOI.	Not Applicable	
Report the **clinical trial registration number** (at ClinicalTrials.gov or equivalent), where applicable.	Not Applicable	

Laboratory protocol	Information included in the manuscript?	In which section is the information available? (Reagents and Tools Table, Materials and Methods, Figures, Data Availability Section)
Provide DOI OR other citation details if **external detailed step-by-step protocols** are available.	Not Applicable	

Experimental study design and statistics	Information included in the manuscript?	In which section is the information available? (Reagents and Tools Table, Materials and Methods, Figures, Data Availability Section)
Include a statement about **sample size** estimate even if no statistical methods were used.	Yes	Figure Captions
Were any steps taken to minimize the effects of subjective bias when allocating animals/samples to treatment (e.g. **randomization procedure**)? If yes, have they been described?	Not Applicable	
Include a statement about **blinding** even if no blinding was done.	Yes	Materials and Methods
Describe **inclusion/exclusion criteria** if samples or animals were excluded from the analysis. Were the criteria pre-established? If sample or data points were omitted from analysis, report if this was due to attrition or intentional exclusion and provide justification.	Not Applicable	
For every figure, are **statistical tests** justified as appropriate? Do the data meet the assumptions of the tests (e.g., normal distribution)? Describe any methods used to assess it. Is there an estimate of variation within each group of data? Is the variance similar between the groups that are being statistically compared?	Yes	Figure Captions, Materials and Methods

Sample definition and in-laboratory replication	Information included in the manuscript?	In which section is the information available? (Reagents and Tools Table, Materials and Methods, Figures, Data Availability Section)
In the figure legends: state number of times the experiment was **replicated** in laboratory.	Yes	Figure Caption
In the figure legends: define whether data describe **technical or biological replicates**.	Yes	Figure Caption

Ethics

Ethics	Information included in the manuscript?	In which section is the information available? (Reagents and Tools Table, Materials and Methods, Figures, Data Availability Section)
Studies involving **human participants**: State details of **authority granting ethics approval** (IRB or equivalent committee(s), provide reference number for approval.	Not Applicable	
Studies involving **human participants**: Include a statement confirming that **informed consent** was obtained from all subjects and that the experiments conformed to the principles set out in the WMA Declaration of Helsinki and the Department of Health and Human Services Belmont Report.	Not Applicable	
Studies involving **human participants:** For publication of **patient photos**, include a statement confirming that consent to publish was obtained.	Not Applicable	
Studies involving experimental **animals**: State details of **authority granting ethics approval** (IRB or equivalent committee(s), provide reference number for approval. Include a statement of compliance with ethical regulations.	Not Applicable	
Studies involving **specimen and field samples:** State if relevant **permits** obtained, provide details of authority approving study; if none were required, explain why.	Not Applicable	

Dual Use Research of Concern (DURC)	Information included in the manuscript?	In which section is the information available? (Reagents and Tools Table, Materials and Methods, Figures, Data Availability Section)
Could your study fall under dual use research restrictions? Please check biosecurity documents and list of **select agents and toxins** (CDC): https://www.selectagents.gov/sat/list.htm	Not Applicable	
If you used a select agent, is the security level of the lab appropriate and reported in the manuscript?	Not Applicable	
If a study is subject to dual use research of concern regulations, is the name of the **authority granting approval and reference number** for the regulatory approval provided in the manuscript?	Not Applicable	

Reporting

The MDAR framework recommends adoption of discipline-specific guidelines, established and endorsed through community initiatives. Journals have their own policy about requiring specific guidelines and recommendations to complement MDAR.

Adherence to community standards	Information included in the manuscript?	In which section is the information available? (Reagents and Tools Table, Materials and Methods, Figures, Data Availability Section)
State if relevant guidelines or checklists (e.g., **ICMJE, MIBBI, ARRIVE, PRISMA**) have been followed or provided.	Yes	MDAR checklist was used
For **tumor marker prognostic studies,** we recommend that you follow the **REMARK** reporting guidelines (see link list at top right). See author guidelines, under 'Reporting Guidelines'. Please confirm you have followed these guidelines.	Not Applicable	
For **phase II and III randomized controlled trials**, please refer to the **CONSORT** flow diagram (see link list at top right) and submit the CONSORT checklist (see link list at top right) with your submission. See author guidelines, under 'Reporting Guidelines'. Please confirm you have submitted this list.	Not Applicable	

Data Availability

Data availability	Information included in the manuscript?	In which section is the information available? (Reagents and Tools Table, Materials and Methods, Figures, Data Availability Section)
Have **primary datasets** been deposited according to the journal's guidelines (see 'Data Deposition' section) and the respective accession numbers provided in the Data Availability Section?	Yes	Data Availability
Were **human clinical and genomic datasets** deposited in a public access-controlled repository in accordance to ethical obligations to the patients and to the applicable consent agreement?	Not Applicable	
Are **computational models** that are central and integral to a study available without restrictions in a machine-readable form? Were the relevant accession numbers or links provided?	Not Applicable	
If publicly available data were reused, provide the respective **data citations in the reference list**.	Not Applicable	
