# Supplementary material for: The regulation of protein phosphatase 4 by FBXO42 is required for cancer cell survival
Source: EMBO Rep. 2026 Jul 14;27(15):4211–37. doi: 10.1038/s44319-026-00828-y (PMC13458699; doi:10.1038/s44319-026-00828-y)
Supplement: Supplementary file 2 [file 44319_2026_828_MOESM8_ESM.pdf]

Expanded View Figures

Figure EV1. Sequences of FBXO42 and CCDC6 knockout clones.

Sanger sequencing results are shown for (**A**). FBXO42 and (**B**). CCDC6 knockout clones, RPE1 cells. Consensus coding sequences (CCDS) are shown for each gene, as is the expected binding site of the sgRNA used to generate knockouts. WT and mutated sequences are aligned with amino acid translations. Each knockout cell line carries a mutation generating a frame shift.

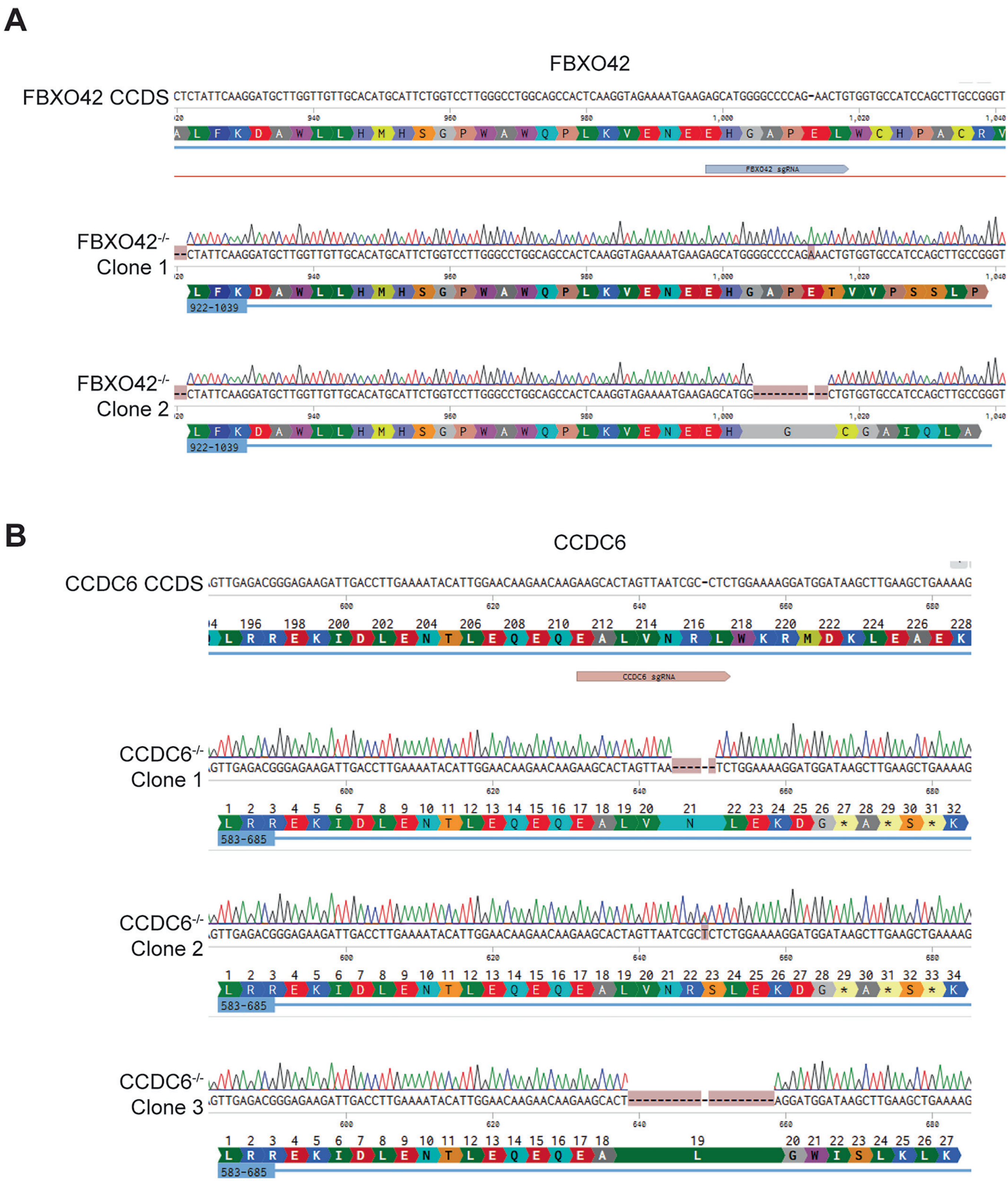
A
FBXO42
FBXO42 CCDS
FBXO42 sgRNA
FBXO42-/- Clone 1
922-1039
FBXO42-/- Clone 2
922-1039
B
CCDC6
CCDC6 CCDS
CCDC6 sgRNA
CCDC6-/- Clone 1
583-685
CCDC6-/- Clone 2
583-685
CCDC6-/- Clone 3
583-685

 © The Author(s)

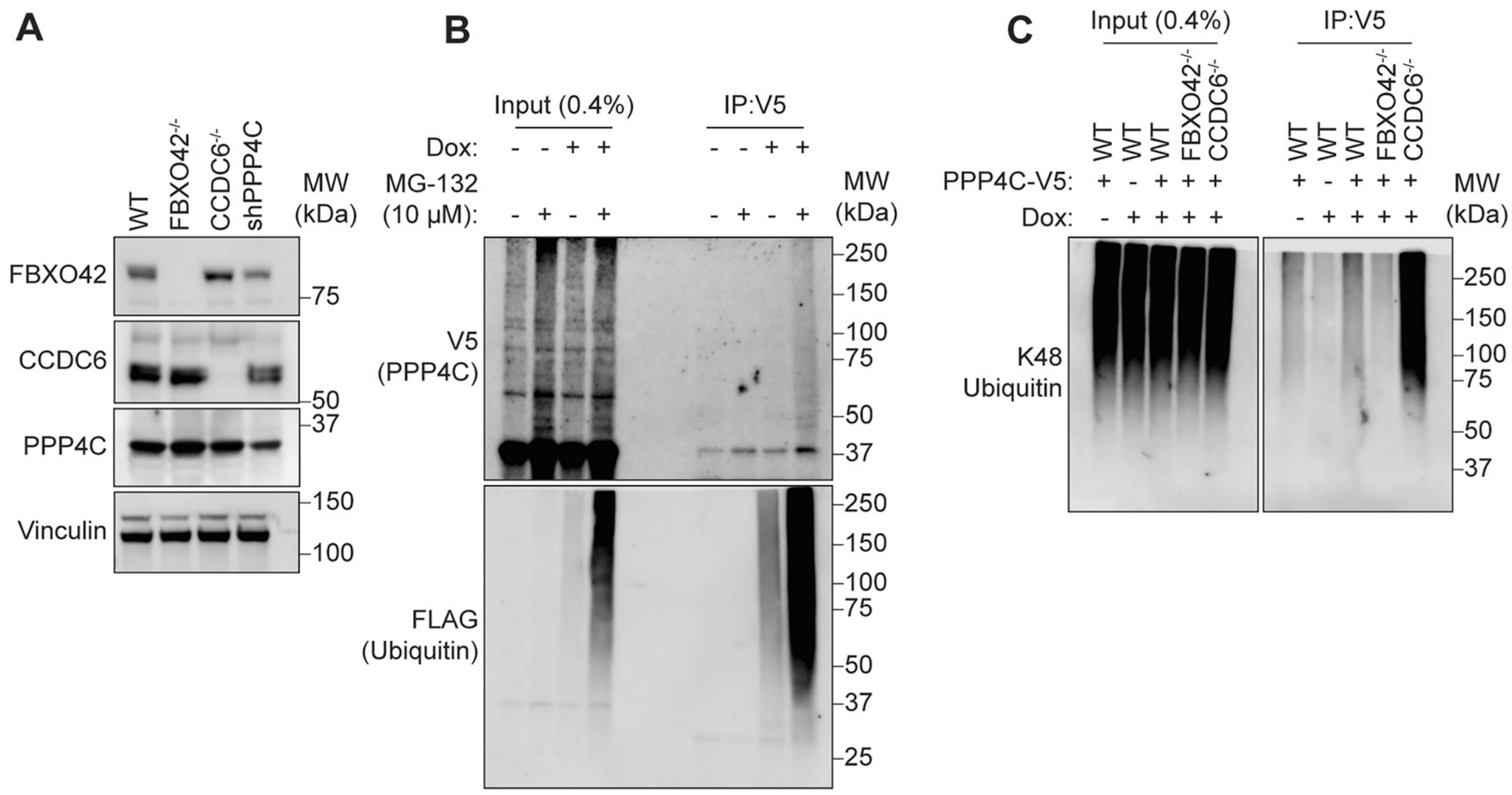

Figure EV2. Interactions of FBXO42, CCDC6, and PPP4C continued.

(**A**) Western blot showing expression of FBXO42, CCDC6, PPP4C, or vinculin (loading control) in the RPE1 cell lines used in Fig. 2A–C. (**B**) Denatured immunoprecipitation western blot performed as in Fig. 3B. FLAG-Ubiquitin expression was induced by treatment with 2 μg/ml doxycycline (Dox) for 24 h where indicated. Cells were treated with MG-132 at 10 μM for 4 h pre-harvest where indicated. (**C**) Western blot with an antibody specific for K48 linked ubiquitin. Same experiment as Fig. 3D.

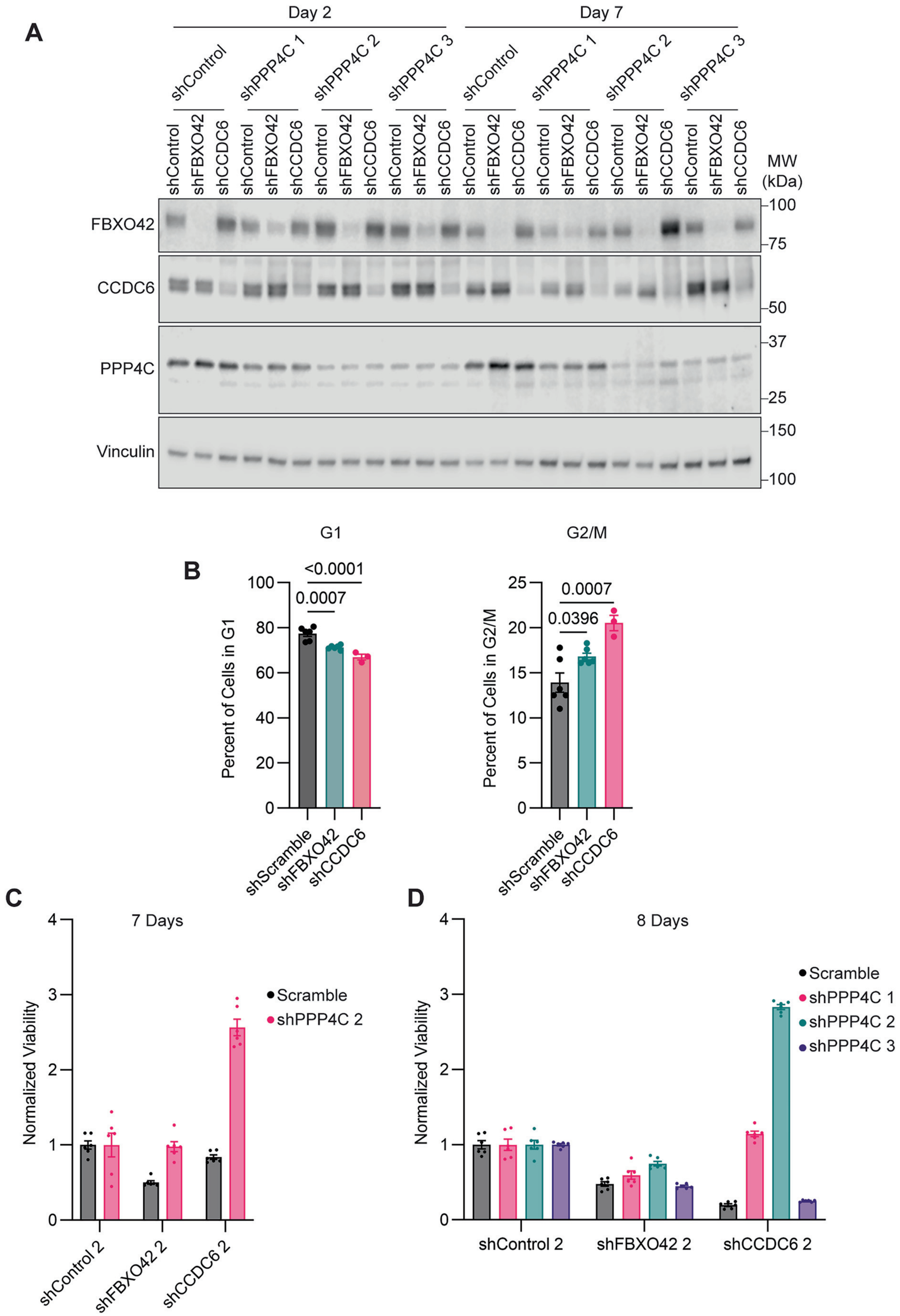
A
Day 2
Day 7
shControl
shPPP4C 1
shPPP4C 2
shPPP4C 3
shControl
shFBXO42
shCCDC6
MW (kDa)
FBXO42
CCDC6
PPP4C
Vinculin
100
75
50
37
25
150
100
B
G1
G2/M
Percent of Cells in G1
Percent of Cells in G2/M
<0.0001
0.0007
0.0396
shScramble
shFBXO42
shCCDC6
C
7 Days
Normalized Viability
Scramble
shPPP4C 2
shControl 2
shFBXO42 2
shCCDC6 2
D
8 Days
Scramble
shPPP4C 1
shPPP4C 2
shPPP4C 3

 © The Author(s)

Figure EV3. The effects of FBXO42 and CCDC6 on PPP4C cell cycle and proliferation in LN-18 cells.

(**A**) Western blot analysis of lysates from LN-18 cells transduced with shRNA against PPP4C or a scramble control. Cells were also, simultaneously, transduced with shRNA targeting FBXO42, CCDC6, or a scramble control. Days post transduction are indicated. (**B**) Flow cytometry-based cell cycle analysis of LN-18 cells transduced with shRNA constructs targeting FBXO42, CCDC6, or a scramble control. Cells were transduced, selected by FACS sorting on GFP, and analyzed 10 days later. Propidium iodine staining was used to assess DNA content. Each point indicates replicate, parallel cell cultures. $N = 6$, bar represents mean ± SEM. P values from two-way ANOVA with Dunnett's multiple hypothesis test. (**C**) A replicate experiment performed similarly to that in Fig. 5A, except that only one anti-PPP4C shRNA ("shPPP4C.2") was used and cells were analyzed at 7 days post transduction, not six. Each point indicates wells of replicate, parallel cell cultures. $N = 6$, bar represents mean ± SEM. (**D**) A replicate experiment performed similarly to that in Fig. 5A, except that cells were analyzed at 8 days post transduction instead of six. Each point indicates wells of replicate, parallel cell cultures. $N = 6$, bar represents mean ± SEM.

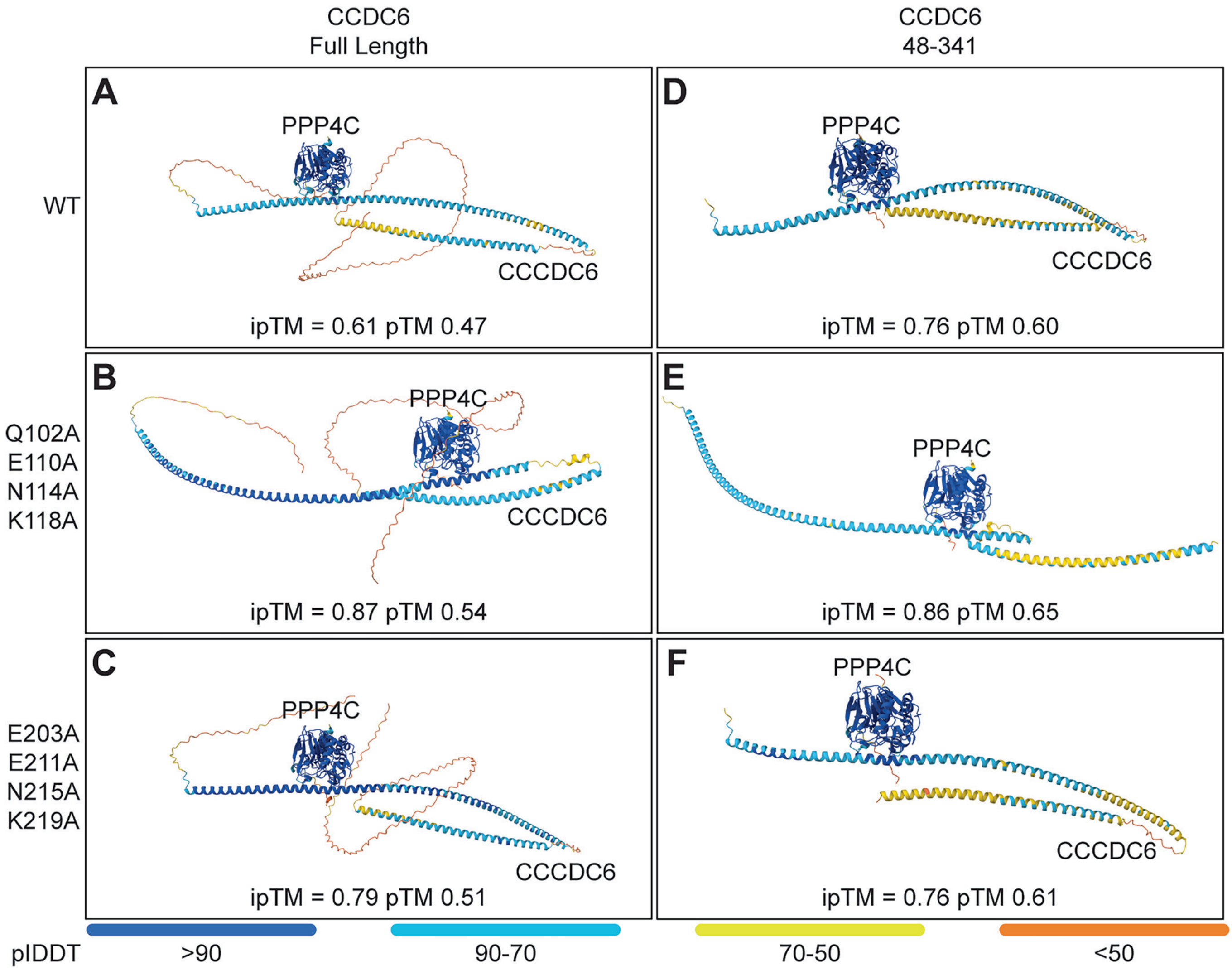
CCDC6
Full Length
CCDC6
48-341
A
PPP4C
WT
CCCDC6
ipTM = 0.61 pTM 0.47
D
PPP4C
CCCDC6
ipTM = 0.76 pTM 0.60
B
Q102A
E110A
N114A
K118A
PPP4C
CCCDC6
ipTM = 0.87 pTM 0.54
E
PPP4C
ipTM = 0.86 pTM 0.65
C
E203A
E211A
N215A
K219A
PPP4C
CCCDC6
ipTM = 0.79 pTM 0.51
F
PPP4C
CCCDC6
ipTM = 0.76 pTM 0.61
pIDDT
>90
90-70
70-50
<50

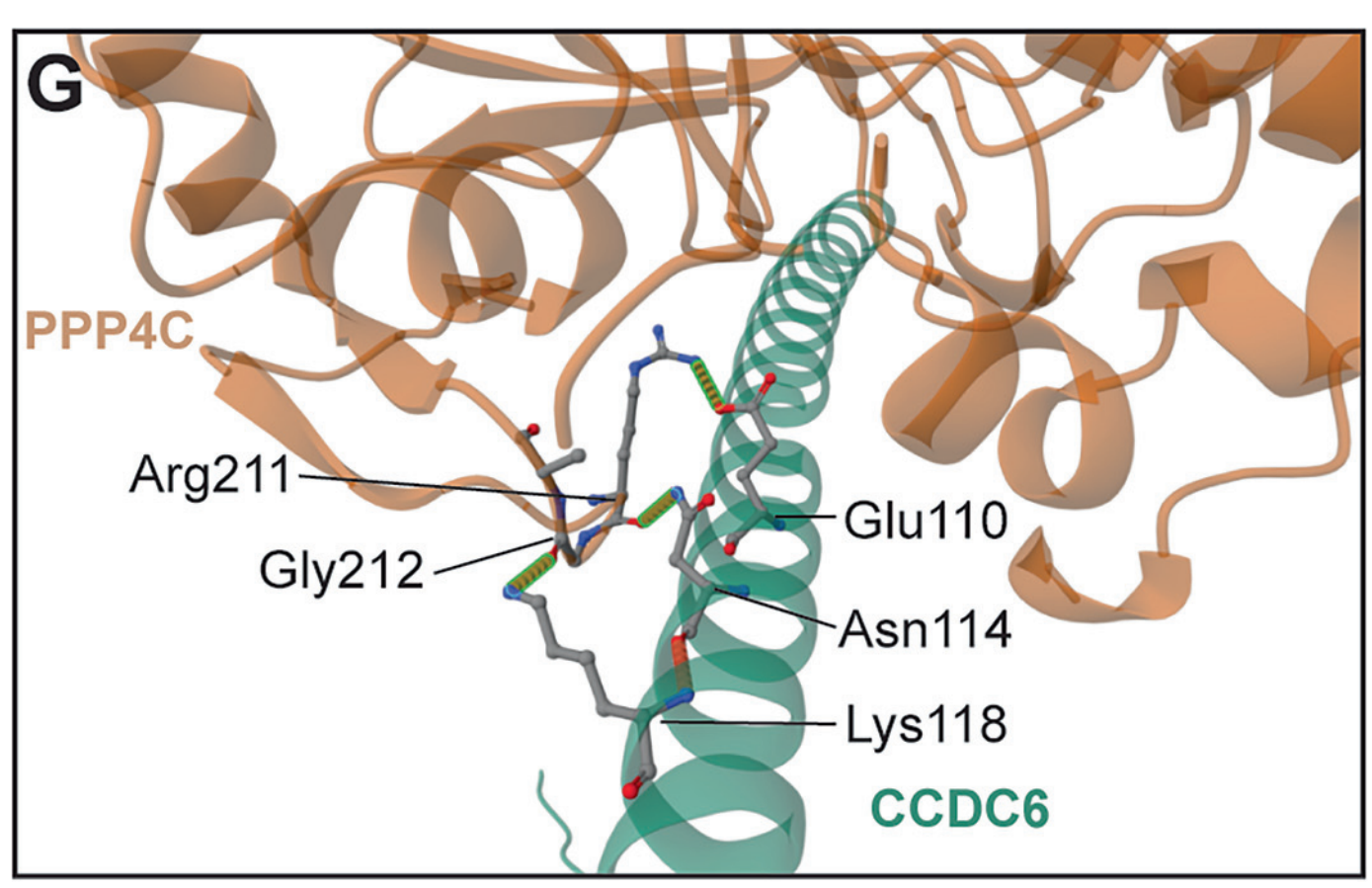
G
PPP4C
Arg211
Gly212
Glu110
Asn114
Lys118
CCDC6

Figure EV4. Alphafold predictions of possible CCDC6-PPP4C interactions.

(**A–F**) Alphafold 3 predictions of CCDC6-PPP4C interactions. ipTM and pTM values shown. Protein representations colored by pIDDT values. (**A–C**) Uses full length CCDC6, (**D–F**) uses CCDC6 with its terminal disordered regions truncated, residues 48–341. (**A**, **D**) Use WT CCDC6. In (**B**, **E**, **C**, **F**), the indicated residues on CDC6 are mutated to alanine to ablate the two different predicted binding sites. Alphafold predictions are theoretical and should be interpreted carefully. (**G**) Alphafold prediction of PPP4C-CCDC6 binding. PPP4C is in orange, CCDC6 is in blue. Hydrogen bonds between the two peptides are highlighted. Amino acids predicted to participate in hydrogen bonding are indicated. Alphafold predictions are theoretical and should be interpreted carefully.

© The Author(s)
